# mRNA structural elements immediately upstream of the start codon dictate dependence upon eIF4A helicase activity

**DOI:** 10.1186/s13059-019-1901-2

**Published:** 2019-12-30

**Authors:** Joseph A. Waldron, David C. Tack, Laura E. Ritchey, Sarah L. Gillen, Ania Wilczynska, Ernest Turro, Philip C. Bevilacqua, Sarah M. Assmann, Martin Bushell, John Le Quesne

**Affiliations:** 1 Cancer Research UK Beatson Institute, Garscube Estate, Switchback Road, Glasgow, G61 1BD UK; 2 Department of Biology, Pennsylvania State University, University Park, PA 16802 USA; 3 Department of Chemistry, Pennsylvania State University, University Park, PA 16802 USA; 4 Present Address: Spectrum Health Office of Research, 100 Michigan Street NE, Mail Code 038, Grand Rapids, MI 49503 USA; 5 Center for RNA Molecular Biology, Pennsylvania State University, University Park, PA 16802 USA; 6 Present Address: Department of Chemistry, University of Pittsburgh at Johnstown, Johnstown, PA 15904 USA; 7 Department of Haematology, University of Cambridge, Cambridge, UK; 8 Medical Research Council Biostatistics Unit, Cambridge Institute of Public Health, Cambridge, UK; 9 National Health Service Blood and Transplant, Cambridge, UK; 10 National Institute for Health Research BioResource, Cambridge University Hospitals, Cambridge, UK; 11 Department of Biochemistry & Molecular Biology, Pennsylvania State University, University Park, PA 16802 USA; 12 Institute of Cancer Sciences, University of Glasgow, Garscube Estate, Switchback Road, Glasgow, G61 1QH UK; 13 Medical Research Council Toxicology Unit, University of Cambridge, Hodgkin Building, Lancaster Road, Leicester, LE1 7HB UK; 14 Leicester Cancer Research Centre, University of Leicester, Leicester, UK

**Keywords:** eIF4A, RNA structure, Hippuristanol, Translation, Translation initiation, Cancer, Structure-seq, DMS, Polysome profiling, G-quadruplexes

## Abstract

**Background:**

The RNA helicase eIF4A1 is a key component of the translation initiation machinery and is required for the translation of many pro-oncogenic mRNAs. There is increasing interest in targeting eIF4A1 therapeutically in cancer, thus understanding how this protein leads to the selective re-programming of the translational landscape is critical. While it is known that eIF4A1-dependent mRNAs frequently have long GC-rich 5′UTRs, the details of how 5′UTR structure is resculptured by eIF4A1 to enhance the translation of specific mRNAs are unknown.

**Results:**

Using Structure-seq2 and polysome profiling, we assess global mRNA structure and translational efficiency in MCF7 cells, with and without eIF4A inhibition with hippuristanol. We find that eIF4A inhibition does not lead to global increases in 5′UTR structure, but rather it leads to 5′UTR remodeling, with localized gains and losses of structure. The degree of these localized structural changes is associated with 5′UTR length, meaning that eIF4A-dependent mRNAs have greater localized gains of structure due to their increased 5′UTR length. However, it is not solely increased localized structure that causes eIF4A-dependency but the position of the structured regions, as these structured elements are located predominantly at the 3′ end of the 5′UTR.

**Conclusions:**

By measuring changes in RNA structure following eIF4A inhibition, we show that eIF4A remodels local 5′UTR structures. The location of these structural elements ultimately determines the dependency on eIF4A, with increased structure just upstream of the CDS being the major limiting factor in translation, which is overcome by eIF4A activity.

## Background

Translational dysregulation is a hallmark of cancer [1–3], and increased activity of the DEAD box RNA helicase, eukaryotic initiation factor 4A1 (eIF4A1), is associated with poor survival in human malignancy [4]. As such, eIF4A1 is an attractive candidate for cancer therapeutics [5–7], with eIF4A specific inhibitors showing promising results in cancer cell lines [8, 9] and mouse models [10–12]. Despite this, it remains unclear how increased eIF4A1 activity can drive the malignant phenotype.

eIF4A1 is thought to function primarily as part of the eIF4F complex, along with the scaffold protein eIF4G and the cap binding protein eIF4E, where it unwinds secondary structure in the 5′UTR of mRNAs [13, 14]. However, the helicase activity of eIF4A is relatively weak compared with other RNA helicases [15], and it may have additional ATPase-dependent but helicase-independent roles, such as remodeling of protein/RNA complexes. Indeed, both human eIF4A1 and yeast eIF4A have been shown to enhance ribosome recruitment onto RNAs lacking secondary structure, implicating a helicase-independent role for eIF4A during translation initiation [16, 17]. Furthermore, while it is clear that eIF4A acts as part of the eIF4F complex, where its helicase activity is dramatically stimulated through its interaction with eIF4B or eIF4H [15], in HeLa cells, levels of eIF4A1 are more than tenfold higher than those of the other core components of the eIF4F complex [18]. Whether excess eIF4A acts as part of the translational machinery or as “free” eIF4A1 is not known, and as such, the consequence of increased levels of eIF4A1 protein, as seen in tumor cells [4], is not clear.

Recent studies have demonstrated that the requirement for eIF4A1 activity is not equal among cellular mRNAs and that those mRNAs that are most translationally repressed following eIF4A inhibition are enriched in transcripts that encode proteins with oncogenic function [4, 11, 19]. As these mRNAs generally possess longer 5′UTRs with increased GC content, it has been presumed that the increased propensity for 5′UTR secondary structures is driving the dependence on eIF4A1. However, predicting secondary structures of mRNAs from sequence alone is highly unreliable, particularly in living cells, as recent studies have shown that in vivo structures can greatly differ from those determined in vitro [20]. For example, the enrichment of a (GGC)_4_ motif in the 5′UTRs of eIF4A1-dependent mRNAs was interpreted as evidence that mRNAs that possess potential 5′UTR G-quadruplex sequences require increased levels of eIF4A1 activity for their translation [11]. However, the prevalence of folded G-quadruplexes within cells remains controversial [21–25]; therefore, the structural determinants of eIF4A dependency remain unclear.

To test the hypothesis that eIF4A-dependent mRNAs have 5′UTR structural features which require increased eIF4A activity for their unwinding, and determine how these mRNAs differ from less sensitive mRNAs, we measured structural changes in RNA in vivo and transcriptome-wide, following eIF4A inhibition with hippuristanol, in a similar approach to that used to study other DEAD-box helicases [26–28]. We used Structure-seq2 [29] to measure the single-strandedness of RNA by specific and rapid methylation of single-stranded adenosines and cytosines with dimethyl sulphate (DMS). Essentially, the more reactive each nucleotide is to DMS, the more confident we can be that it is single-stranded. It should be noted that although single-strandedness can be confidently inferred by DMS reactivity, it is not currently possible to rule out that highly protected regions at least in part arise from protein protection, although protection from eIF4A should be minimal as eIF4A binds the RNA backbone [30], and DMS methylates the Watson-Crick face of adenines and cytosines [31]. We coupled our Structure-seq2 data with polysome profiling so that we could correlate changes in RNA structure with translation. Hippuristanol was used to inhibit eIF4A, as this causes a loss of both its RNA binding and its ATPase activity, by locking the protein in its closed confirmation [32], thereby achieving a loss of function. This is preferable to alternative eIF4A inhibitors, which act in a gain of function manner on a subset of mRNAs, by stimulating the RNA binding and ATPase activity of eIF4A at polypurine rich sequences [33].

Our data show that upon eIF4A inhibition, 5′UTRs are remodeled, with certain regions becoming more structured, while adjacent segments lose structure. eIF4A-dependent mRNAs have greater localized gains of structure, and crucially, these highly structured elements are located predominantly at the 3′ end of 5′UTRs. We propose a model in which increased structure potential just upstream of the coding sequence is the key determinant of preferential expression upon the translational reprogramming which occurs following increased eIF4A levels in malignancy.

## Results

### Measuring eIF4A mediated changes in RNA structure

**Fig. 1:**
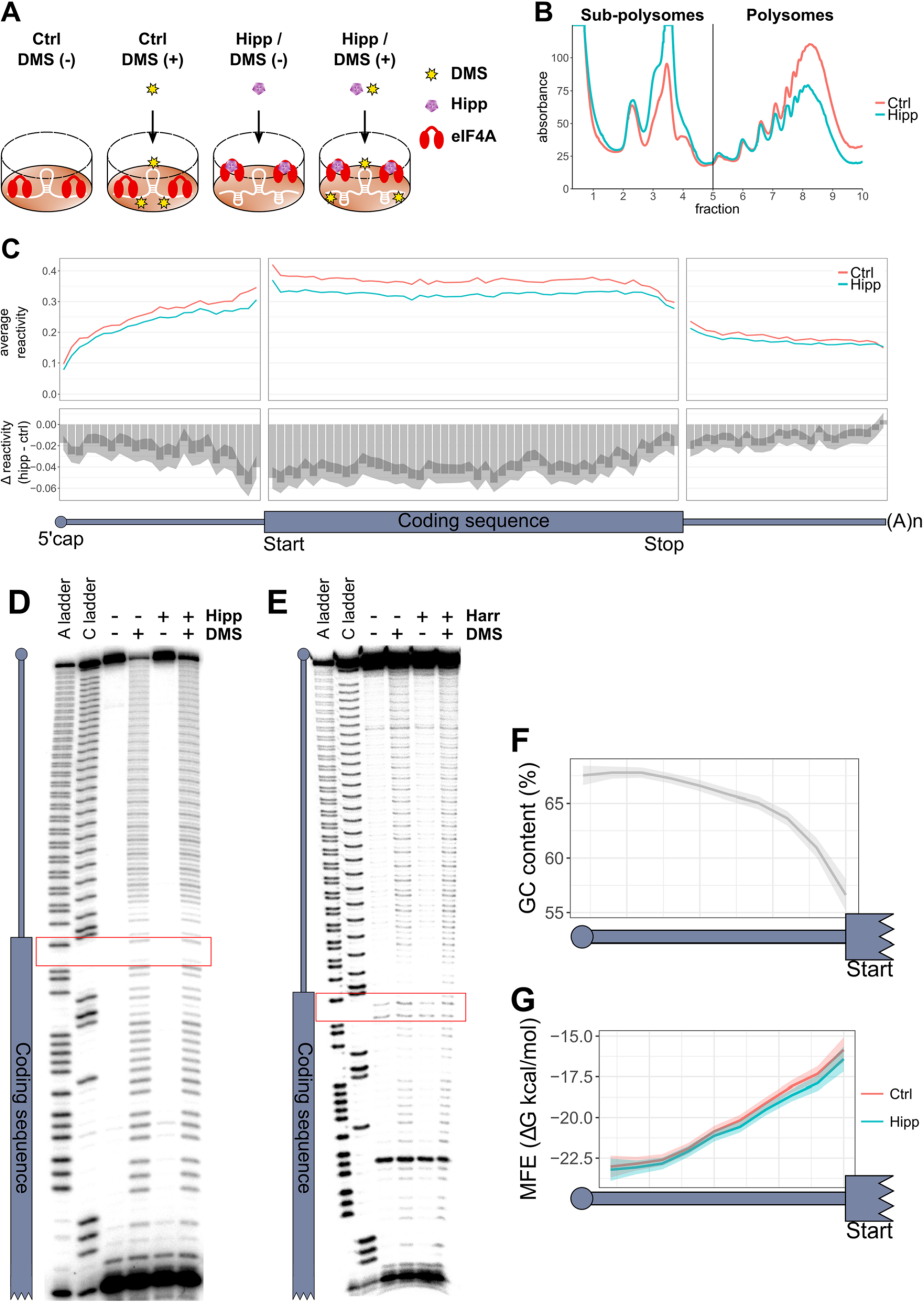
5′UTRs are innately more structured at their 5′ ends. **a** A diagrammatic representation of the experimental design. MCF7 cells were treated for 1 h with or without 150 nM hippuristanol (Hipp), followed by 10-min treatment with or without 50 mM DMS. **b** A representative polysome trace from three biological replicates for control (Ctrl) and hippuristanol (Hipp)-treated cells. See Additional file 1: Figure S1B-C for additional two replicates. **c** The top panel plots the binned average reactivity for control (Ctrl) and hippuristanol (Hipp) samples across the length of the UTRs (25 bins) and coding sequence (50 bins). The bottom panel plots the Δ reactivity, which is calculated by subtracting control from hippuristanol. Therefore, a negative value indicates decreased reactivity and therefore increased structure following hippuristanol treatment, whereas a positive value indicates less structure following hippuristanol treatment. Shaded area represents 95% confidence limits for the difference in means between control and hippuristanol mRNAs within each bin, calculated by a paired two-sided *t* test. All 1266 mRNAs included in the analysis have a 5′UTR, CDS, and 3′UTR at least 100 nt in length; have sufficient coverage and 5′ end coverage; and are the most abundant transcript per gene. **d**, **e** Sequencing gels showing the DMS reactivity of a reporter with an unstructured (CAA)_24_ 5′UTR (see the “Methods” section) with and without either 1 μM hippuristanol (Hipp) or 20 μg/ml harringtonine (Harr) in nuclease untreated rabbit reticulocyte lysate. The gels show the cDNA following reverse transcription using a primer which binds within the coding sequence of the reporter (strong band at the bottom of the gel). Full length product is the band at the top of the gel, and the position of all aborted products is denoted by the diagram of the transcript to the left, with the red boxes highlighting the position of the start codon. **A** and **C** ladders were created exactly as the sample lanes, but with the addition of ddTTP and ddGTP respectively. **f** Mean binned GC content of all 50-nt windows, with a step of 10 nt, within the 5′UTR of all transcripts included in panel **c**. Shaded area represents 95% confidence intervals of the mean. **g** Mean binned minimum free energy (MFE) of all 50-nt windows, with a step of 10 nt, after folding with restraints derived from DMS reactivities under control or hippuristanol conditions, within the 5′UTRs of all transcripts included in panel **c**. Shaded area represents 95% confidence intervals of the mean

To determine the effect of eIF4A activity on RNA secondary structure in vivo, we measured the reactivity of cellular RNA to dimethyl sulphate (DMS) following eIF4A inhibition with hippuristanol (hipp) in MCF7 cells (Fig. 1a). In order to primarily inhibit the translation of eIF4A-dependent mRNAs, rather than to completely ablate global translation, MCF7 cells were treated with hipp for 1 h at the IC_50_, as determined by ^35^S protein labeling (Additional file 1: Figure S1A). This causes a large increase in sub-polysomal RNA and a marked reduction in polysomal RNA (Fig. 1b and Additional file 1: Figure S1B-C), consistent with an inhibition of translation initiation.

As DMS methylates un-paired adenosine and cytosine residues, the accessibility of these nucleotides to DMS can be interpreted as the extent to which they are single-stranded within the cell. After treatment with DMS, under single-hit kinetics (Additional file 1: Figure S1D), RNA is extracted and the sites of DMS modification are identified using reverse transcription with random primers on poly(A) selected mRNA (Additional file 1: Figure S1E). As the sites of DMS methylation are on the Watson-Crick face of adenosine and cytosine residues [31], the reverse transcriptase enzyme stops at these positions. Subsequent library preparation steps using Structure-seq2 methodologies (Additional file 1: Figure S1E) (see the “Methods” section) allow these reverse transcriptase stop sites to be quantified following Illumina next-generation sequencing. DMS untreated samples were prepared in parallel to allow subtraction of non-DMS derived reverse transcriptase stops. The StructureFold2 bioinformatic pipeline [34] was used to calculate DMS reactivity transcriptome-wide (see the “Methods” section). To assess the quality of our libraries, the percentage of each nucleotide responsible for each reverse transcriptase stop was calculated. In DMS (+) samples, this was over 85% adenines and cytosines, but was divided much more evenly across the four nucleobases in the DMS (−) samples (Additional file 1: Figure S1F), with no evidence for any ligation bias (Additional file 1: Figure S1G). Replicate correlation was determined between the three biological repeats for each sample. This ranged from 0.71 to 0.84 for the DMS (−) samples and 0.85 to 0.88 for the DMS (+) samples, across the whole transcriptome (Additional file 1: Figure S2A). To determine a suitable coverage threshold, we plotted the correlation coefficients between replicates for all transcripts after filtering with different coverage thresholds within each replicate (Additional file 1: Figure S2B). We decided that a threshold of one was most suitable, and the correlation matrix table in Additional file 1: Figure S2C shows that transcriptome-wide correlation within each replicate to be above 0.91 for all samples at this coverage threshold. Importantly, control and hipp DMS (−) samples but not the DMS (+) samples were also highly correlated (Additional file 1: Figure S2C), consistent with hipp treatment not leading to any changes in natural reverse transcriptase stops.

Changes in RNA structure following eIF4A inhibition can be inferred by reactivity changes between control and hipp conditions, where decreased reactivity can be interpreted as increased structure and vice versa. In order to confidently measure changes in DMS reactivity, it is essential that the transcriptome used for the bioinformatic pipeline is a true representation of the transcriptome within the cell. This is particularly important given our interest in 5′UTRs and recent findings that true 5′ ends often differ from even manually curated transcripts [35]. We therefore used our sequencing reads to assess the accuracy of 5′ end annotation between manually curated RefSeq transcripts, a transcriptome based on nanoCAGE data from MCF7 cells [35], and a MCF7-specific transcriptome based on long-range sequencing reads from Pacific Biosciences (see the “Methods” section) (Additional file 1: Figure S3A-B). Our analysis showed that the two transcriptomes which were based on sequencing data from MCF7 cells far better reflected the true 5′ ends of our sequencing data, compared to the RefSeq transcriptome. Unsurprisingly, the nanoCAGE data are superior in 5′ end annotation, but as the MCF7-specific transcriptome has sequence information for the whole transcript, we decided to use this transcriptome for our analyses. In addition, we created a 5′ end coverage score to remove transcripts from further analysis if their true 5′ end likely differed from the MCF7-specific transcriptome annotation (Additional file 1: Figure S3B and see the “Methods” section). It should be noted that the 3′ most 125 nt of the 3′UTRs are removed prior to any analysis, due to lack of Structure-seq2 coverage of the 3′ ends of transcripts (Additional file 1: Figure S3C); the remaining region is subsequently referred to as the 3′ region.

### Coding sequences gain in structure more than UTRs following eIF4A inhibition

To assess the changes in RNA structure within the UTRs and CDSs following eIF4A inhibition, we plotted the average reactivity within each region for all transcripts in control and hipp-treated samples (Additional file 1: Figure S4A-C). Interestingly, the biggest difference was seen in the CDS, with the majority of CDSs becoming less reactive to DMS following hipp treatment (Additional file 1: Figure S4B), indicating increased average structure overall. This could implicate a role for eIF4A in unwinding structure within the CDS, but is most likely caused by translational repression leading to reduced ribosome occupancy. Elongating ribosomes are known to unwind RNA secondary structures, and indeed, two recent studies identified a positive correlation between ribosome occupancy and DMS reactivity [36, 37].

There is a statistically significant decrease in the mean average reactivity across all 5′UTRs following hipp treatment, indicating an overall trend to becoming more structured following eIF4A inhibition (Additional file 1: Figure S4A, top panel). However, plotting the change in reactivity of every individual 5′UTR (Additional file 1: Figure S4A, bottom panel) shows that similar numbers of 5′UTRs become more and less structured overall. This is therefore consistent with eIF4A inhibition leading to remodeling of 5′UTR structure rather than increased structure throughout. The decreased reactivity we observe in the 5′UTR is unlikely to be due to 43S accumulation within 5′UTRs, as this would be expected to do the reverse; however, increased reactivity within this region could be explained by paused scanning 43S ribosomal subunits. To further evaluate, we folded 100-nt 5′UTR windows, using the DMS reactivities as structural restraints, and plotted both the minimum and average minimum free energy (MFE), and the maximum and average percentage of base-paired nucleotides (strandedness) for each transcript, from the predicted folds (Additional file 1: Figure S4D-G). Although statistically significant, the differences are very small. This could indicate either very little change in RNA structure following eIF4A inhibition, or refolding of the RNA, so that certain regions become more structured, with adjacent regions becoming less structured, which would not lead to large changes in the MFE.

The mean change in average reactivity was smallest in the 3′UTRs (Additional file 1: Figure S4C top panel), with fewer individual 3′UTRs changing in reactivity following hipp treatment (Additional file 1: Figure S4C bottom panel). As eIF4A is not thought to act within the 3′UTR, it is likely any changes are indirect consequences of general rearrangements in mRNA structure following translational inhibition. We have therefore decided not to focus on these.

To assess localized changes in structure, we calculated the Gini coefficient [20, 38] which is a commonly used measurement of inequality within a set of numbers. A Gini coefficient of one indicates an unequal distribution whereas zero indicates perfect evenness. For example, if a transcript/region had a high Gini coefficient, all the reactivity would be restricted to a small percentage of nucleotides, whereas a low Gini coefficient would indicate evenly shared reactivity among all nucleotides. Overall Gini coefficients increased for the majority of transcripts in both UTRs and the CDS following hipp treatment (Additional file 1: Figure S4H-J). This is consistent with an increase in the stability of localized secondary structures following eIF4A inhibition, which would cause base-paired regions to become less accessible and internal bulges and loops more accessible, resulting in reactivities further towards the extremes of their range.

### 5′UTRs are most structured away from the coding sequence

To visualize reactivity within the transcripts, we plotted the binned reactivity across the length of each UTR and CDS (Fig. 1c) and the reactivity of the first and last 60 nt of each region (Additional file 1: Figure S5A). This showed that 5′UTRs have greater DMS reactivity towards the CDS, i.e., are most structured at their extreme 5′ ends, in both control and hipp conditions. As DMS-sequencing data contains more stops at adenines than cytosines (Additional file 1: Figure S1F) [39, 40], we tested whether this pattern in reactivity was due to differing ratios of adenines to cytosines by plotting the binned reactivity pattern for adenines and cytosines separately (Additional file 1: Figure S5B-C). As the reactivity pattern was present for both nucleotides, this suggests that 5′UTRs become increasingly more accessible to DMS towards the CDS. To test if 5′ end protection is due to structure or to protection by cap-binding cellular machinery, we designed an experiment to measure DMS reactivity within a structure-less 5′UTR (Additional file 1: Figure S5D) in nuclease untreated rabbit reticulocyte lysate, which recapitulates cap-dependent translation [41]. The pattern of reactivity within the 5′UTR was even throughout (Fig. 1d), unlike the reactivity in 5′UTRs globally (Fig. 1c). Furthermore, when we inhibited translation of our reporter mRNA with hipp (Additional file 1: Figure S5E), which would reduce binding of eIF4A and the ribosomal machinery to the reporter mRNA, we saw no change in the reactivity pattern within its 5′UTR (Fig. 1d). We also ruled out the possibility that the ribosome could be protecting from DMS reactivity, by adding harringtonine to this assay. Harringtonine traps the 80S ribosome on the start codon [42]; therefore, if the ribosome could protect from DMS reactivity, we would expect to see increased protection over the start codon following translational repression with harringtonine (Additional file 1: Figure S5F), which we do not observe (Fig. 1e). This supports the interpretation that 5′UTRs are less accessible to DMS at their 5′ ends due to increased structure.

To see if greater structure towards the 5′ end was an innate sequence-driven feature of 5′UTRs, we determined the GC content and MFE of predicted folds for all 50-nt windows, across the length of the 5′UTRs, following a sliding window approach with steps of 10 nt (Fig. 1f, g). This clearly mirrors the pattern we see in reactivity (Fig. 1c), in that 5′UTRs are more GC-rich and structured towards the 5′ end. It therefore seems to be an intrinsic property of 5′UTR sequences to have less structure formation nearer to the CDS, and that this is driven at least in part by GC content.

Although 5′UTRs are more structured at their 5′ ends, it is actually at the 3′ end of 5′UTRs that we see the biggest changes in reactivity following eIF4A inhibition (Fig. 1c and Additional file 1: Figure S5A), indicating that the 5′ ends generally remain structured following eIF4A inhibition while the regions close to the CDS gain in structure the most. This is consistent with a specific inhibition of scanning. An alternative explanation is that increased structure in this region could be due to reduced ribosome occupancy in upstream open reading frames (uORFs). To test this, we made use of global translation initiation sequencing (GTI-seq) data, taken from Lee et al. [43], which maps translation start sites in HEK293 cells. Although these data are from an alternative cell line, no data are currently available for MCF7 cells. Based on these data, we restricted the analysis to only those genes which we can be most confident have no potential for upstream translation initiation, by selecting genes that initiated translation solely from the annotated translation initiation start site (aTIS). If the decreased reactivity at the 3′ end of the 5′UTR following hipp treatment was caused by reduced ribosome occupancy in uORFs, then we would not expect to see this in the aTIS transcripts. As this reduction in reactivity is still observed in these transcripts (Additional file 1: Figure S5G-H), this argues against the increased structure at the 3′ end of the 5′UTRs being caused by reduced ribosome occupancy within uORFs.

The CDS is more reactive across its entire length than both the UTRs (Fig. 1c). This is in agreement with Beaudoin et al. [36] and Mizrahi et al. [37] who claim this is a consequence of ribosome occupancy, leading to the unwinding of CDS secondary structure. Decreased reactivity following hipp treatment is observed across the length of the CDS, but the Δ reactivity diminishes towards the 3′ end (Fig. 1c). If the changes in reactivity in the CDS are being mediated by the elongating ribosome, then this might indicate generally reduced ribosome density towards the 3′ end of CDSs.

### Ribosome occupancy is correlated with DMS reactivity

To investigate the correlation between RNA secondary structure and translation, polysome profiling was carried out in parallel, which quantifies translational efficiency based on the enrichment of mRNA in the polysomal over the sub-polysomal fractions, following separation on a sucrose density gradient (see the “Methods” section). Polysome profiling was chosen over ribosome footprinting as we did not require single-nucleotide resolution of ribosome positioning in the coding sequences of the mRNA, and polysome profiling is a simpler technique that is thought to be more sensitive at identifying less abundant mRNAs with smaller shifts in translation efficiency [44]. The traces acquired during the fractionation for each biological repeat are shown in Fig. 1b and Additional file 1: Figure S1B-C. Fractions 1–5 and 6–11 were each pooled to comprise the sub-polysomal and polysomal RNA respectively, and along with total RNA samples, were analyzed by RNA-Seq (see the “Methods” section).

**Fig. 2:**
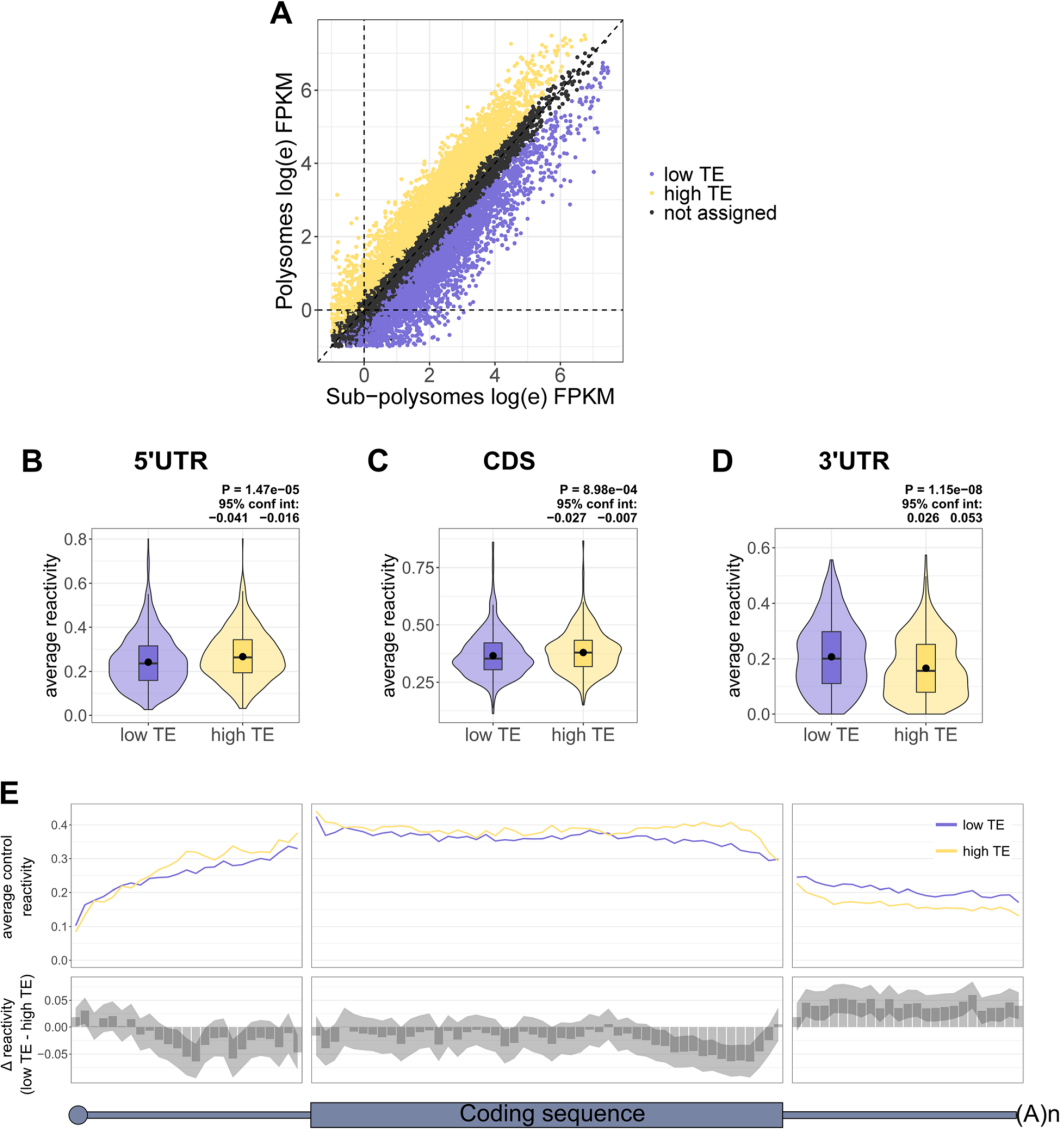
Highly translated mRNAs are more reactive to DMS in the coding region and 3′ end of the 5′UTR. **a** A scatter plot of the log(e) fragments per kilobase million (FPKM) in the sub-polysomal and polysomal fractions, color coded by the top (high TE) and bottom (low TE) third of genes ranked by the translational efficiency (TE), which is calculated as a ratio of polysomal to sub-polysomal RNA. **b**–**d** Violin plots depicting the average reactivity under control conditions in the 5′UTRs, CDSs, and 3′UTRs, for the top and bottom third of mRNAs ranked by TE after filtering by coverage and 5′ end coverage and selecting the most abundant transcript per gene. Violin plots include boxplots, with the mean denoted by a dot. *P* values and 95% confidence intervals were calculated using an un-paired, two-sided Wilcoxon test. Each group contains 627 mRNAs. **e** Binned average reactivity, under control conditions, for the top and bottom third of mRNAs ranked by TE, after removing mRNAs whose 5′UTR, CDS, or 3′UTR is shorter than 100 nt, filtering by coverage and 5′ end coverage and selecting the most abundant mRNA per gene. There are 422 mRNAs in each group. The top panel plots the binned average reactivity under control conditions for all the low TE and high TE mRNAs, across the length of the UTRs (25 bins) and coding sequence (50 bins). The bottom panel plots the Δ reactivity between the low TE and the high TE group, which is calculated by subtracting the high TE from the low TE; therefore, a negative value indicates increased reactivity and therefore less structure in the high TE group, whereas a positive value indicates more structure in the high TE group. Shaded area represents 95% confidence limits for the difference in means between the two groups of mRNAs within each bin, calculated by an un-paired two-sided *t* test

To test for a correlation between ribosome occupancy and DMS reactivity in the CDS, we selected the top and bottom third of mRNAs, ranked by their translational efficiency (TE) under control conditions (Fig. 2a), and plotted the average reactivity for each region (Fig. 2b–d) and the binned reactivity across the transcript (Fig. 2e). This clearly shows that highly translated mRNAs (high TE group) are significantly more reactive in the CDS compared with translationally repressed mRNAs (low TE group) (Fig. 2c, e), and this is most pronounced towards the 3' end of the CDS. This further supports the findings from Beaudoin et al. [36] and Mizrahi et al. [37], suggesting that the elongating ribosome is responsible for unfolding the mRNA within the CDS.

The average 5′UTR reactivity was also significantly higher in the high TE group compared to the low TE mRNAs (Fig. 2b). Interestingly, it is only within the 3′ half of the 5′UTRs (Fig. 2e), particularly within the last 20 nt (Additional file 1: Figure S6A), that the high TE mRNAs are more reactive, and surprisingly, these mRNAs are less reactive at the extreme 5′ ends of their 5′UTRs (Fig. 2e). To test whether the high TE group is enriched in mRNAs that are initiating translation upstream, we again turned to the GTI-seq data [43] to calculate an upstream translation initiation site (uTIS) score for each gene. This is calculated by dividing the number of reads mapped to upstream start sites by the number of reads mapped to both upstream and the annotated start sites. A score of zero would indicate no upstream initiation, whereas a score of one would indicate initiation only at upstream sites. This analysis showed no significant difference in uTIS scores between the two groups of mRNAs (Additional file 1: Figure S6B), suggesting that reduced structure just upstream of CDSs in highly translated mRNAs is not due to upstream translation initiation.

Interestingly, there is increased reactivity throughout the length of the 3′UTR in the low TE mRNAs, compared to the high TE group, which could reflect altered protein binding based on the translational status of the mRNAs.

### eIF4A-dependent 5′UTRs are not enriched in potential G-quadruplex sequences

**Fig. 3:**
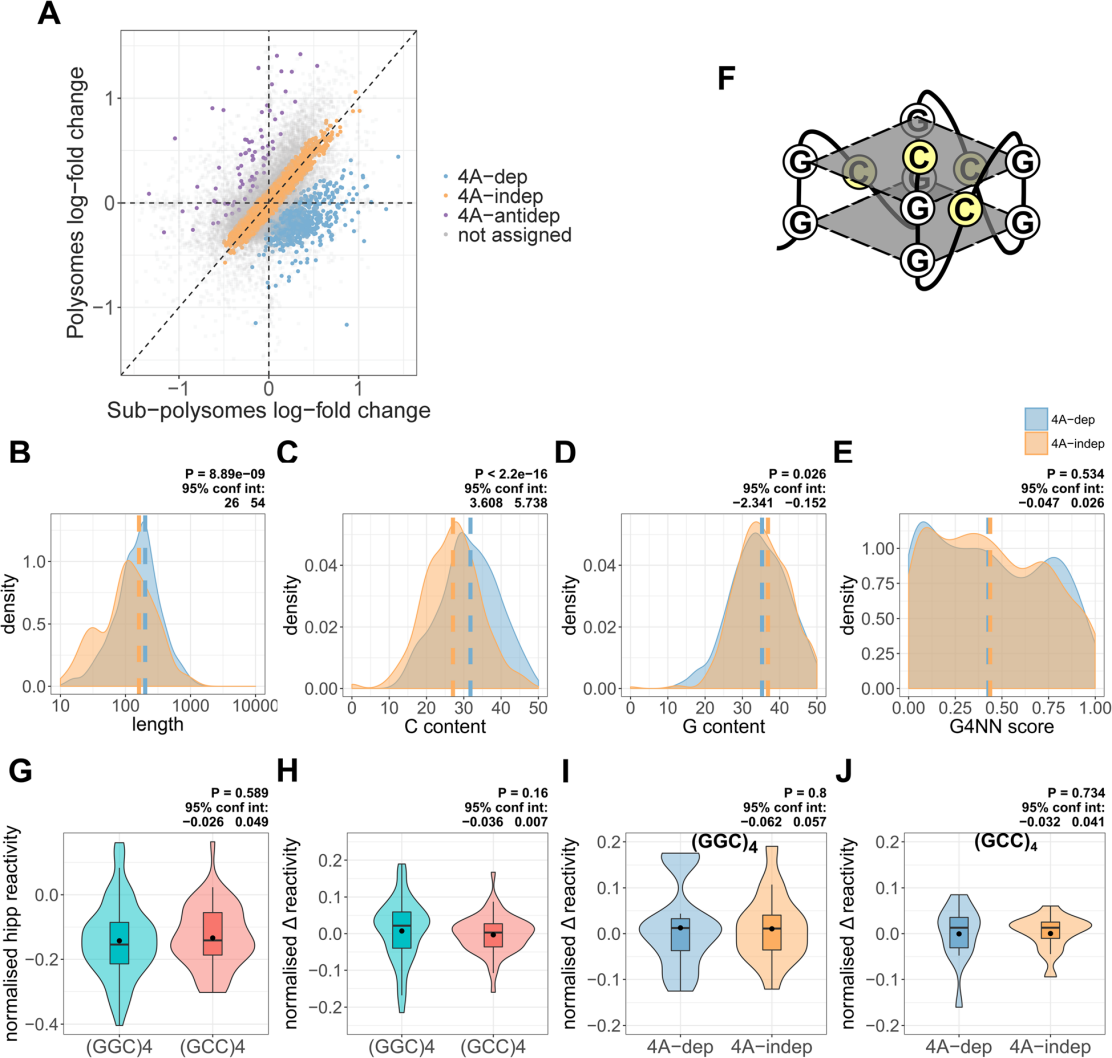
eIF4A-dependent mRNAs are not enriched in G-quadruplexes. **a** A scatter plot depicting the log-fold change in expression between hippuristanol and control, in the polysomal and sub-polysomal fractions, for all 16,868 genes in the MCF7-specific transcriptome. A negative log-fold change indicates reduced expression following hippuristanol treatment and vice versa. The plot is color coded by those mRNAs identified as either eIF4A-dependent (4A-dep) (728), eIF4A-independent (4A-indep) (4587), or eIF4A-antidependent (4A-antidep) (78) or those that were not assigned to each category (see the “Methods” section). **b**–**e** Density plots, showing 5′UTR length, C content, G content, and G4NN scores (see the “Methods” section) for 4A-dep and 4A-indep mRNAs. For those mRNAs which we were able to determine the UTR boundaries (see the “Methods” section), the most abundant transcript per gene was selected. An equal group size of 4A-indep mRNAs was created by selecting the mRNAs with the lowest posterior probability, i.e., those which with most confidence are 4A-indep. This resulted in 441 mRNAs per group. *P* values and 95% confidence intervals were calculated using an un-paired, two-sided Wilcoxon test. **f** A diagrammatic representation of a (GGC)_4_ sequence folded into a G-quadruplex, with the cytosine residues highlighted in yellow, indicating their accessibility to DMS. **g** Normalized average reactivity of all (GGC)_4_ and (GCC)_4_ motifs within 5′UTRs. One motif per 5′UTR was randomly selected, which resulted in 91 (GGC)_4_ and 54 (GCC)_4_ motifs. The reactivity of the motif was normalized by subtracting the average reactivity for the corresponding 5′UTR. *P* values and 95% confidence intervals were calculated using an un-paired, two-sided Wilcoxon test. **h** Normalized average Δ reactivity of the (GGC)_4_ and (GCC)_4_ motifs from panel G. The Δ reactivity of the motif was normalized by subtracting the average Δ reactivity for the corresponding 5′UTR. *P* values and 95% confidence intervals were calculated using an un-paired, two-sided Wilcoxon test. **i**, **j** Normalized Δ reactivity of (GGC)_4_ and (GCC)_4_ motifs, compared between 4A-dep and an equal-sized group of 4A-indep mRNAs. There are 16 (GGC)_4_ and 15 (GCC)_4_ motifs in each group. *P* values and 95% confidence intervals were calculated using an un-paired, two-sided Wilcoxon test

To identify mRNAs that are most translationally repressed following eIF4A inhibition and those that are relatively insensitive, we used a Bayesian model to identify mRNAs that with greatest confidence had shifted from the polysomal into the sub-polysomal fraction, following hipp treatment and those mRNAs that did not change in their polysomal to sub-polysomal ratio, which were termed eIF4A-dependent (4A-dep) and eIF4A-independent (4A-indep) mRNAs respectively (Fig. 3a) (see the “Methods” section). The model also identified those mRNAs that had shifted from the sub-polysomal to polysomal fractions, which were termed eIF4A-antidependent mRNAs (Fig. 3a). However, unsurprisingly, given that very few mRNAs are expected to increase their rate of translation following eIF4A inhibition, this group of mRNAs was too small to use for any downstream analysis. To test for overlap between previously published eIF4A-dependent mRNAs, we plotted a Venn diagram containing the hipp-sensitive mRNAs from Iwasaki et al. [33], using ribosome footprinting following 1 μM hipp treatment in HEK293 cells and the eIF4A1-dependent mRNAs identified by Modelska et al. [4], using polysome profiling following knock-down of eIF4A1 with siRNA (Additional file 1: Figure S7A). We found a better overlap with the eIF4A1-dependent mRNAs identified by Modelska et al. (we identified 33.7% of the eIF4A1-dependent mRNAs from this study), than with the hipp-sensitive mRNAs identified by Iwasaki et al. (we identified 17.3% of the hipp-sensitive mRNAs from this study), suggesting that the use of the same cell line and technique leads to a higher overlap than a similar approach to eIF4A inhibition.

As previous studies have shown that 4A-dep mRNAs have longer more GC rich 5′UTRs than 4A-indep mRNAs [4, 11, 19], we again looked at these properties in our groups of transcripts. Indeed, both 5′UTR length (Fig. 3b) and C content (Fig. 3c), but not G content (Fig. 3d) are increased in 4A-dep mRNAs. It is interesting that G content is not increased, given that the enrichment of a (GGC)_4_ motif in the 5′UTRs of 4A-dep mRNAs had previously been interpreted as implicating eIF4A activity in unwinding G-quadruplexes [11]. To test specifically for an enrichment of G-quadruplex sequences, we used G4RNA screener [45] to predict the likelihood of G-quadruplex folding within the 5′UTRs of these groups of mRNAs. This showed no significant enrichment of potential G-quadruplex sequences in 4A-dep mRNAs compared to 4A-indep mRNAs (Fig. 3e).

The cytosines within a (GGC)_4_ motif that has folded into a G-quadruplex would be within the loop position of the quadruplex (Fig. 3f). We therefore reasoned that the reactivity of these cytosines to DMS should be higher when these sequences are folded into a G-quadruplex than when folded into canonical Watson-Crick based structures, due to increased accessibility, as is seen with the SHAPE reagent NAI [23, 46]. To further evaluate whether 5′UTR (GGC)_4_ sequences were likely folded into G-quadruplexes following eIF4A inhibition in cells, we plotted the normalized reactivity of (GGC)_4_ motifs under hipp conditions. We compared this normalized reactivity to the reverse complement (GCC)_4_ sequence, which has no G-quadruplex folding potential. To normalize the reactivity of each motif, we subtracted the average reactivity of the whole 5′UTR from the average reactivity of the motif. There was no significant difference in normalized reactivity between (GGC)_4_ and (GCC)_4_ motifs (Fig. 3g), further supporting that these (GGC)_4_ motifs fold into canonical Watson-Crick based structures rather than G-quadruplexes [24]. To assess for changes in reactivity following eIF4A inhibition, we compared the Δ reactivity, again normalized to the average Δ reactivity of the whole 5′UTR, which was also not significantly different between the (GGC)_4_ and (GCC)_4_ motifs (Fig. 3h). Finally, as it may be possible that the (GGC)_4_ sequences are folded into G-quadruplexes only in 4A-dep mRNAs, we compared the normalized Δ reactivity between 4A-dep and 4A-indep mRNAs for the (GGC)_4_ (Fig. 3i) and (GCC)_4_ (Fig. 3j) motifs and there was no significant difference between the two groups of mRNAs for either motif. Taken together, these data suggest that enrichment of (GGC)_4_ motifs in 4A-dep mRNAs is not due to their potential to fold into G-quadruplexes.

### Increased structure just upstream of the coding sequences following hippuristanol treatment is most pronounced in eIF4A-dependent mRNAs

**Fig. 4:**
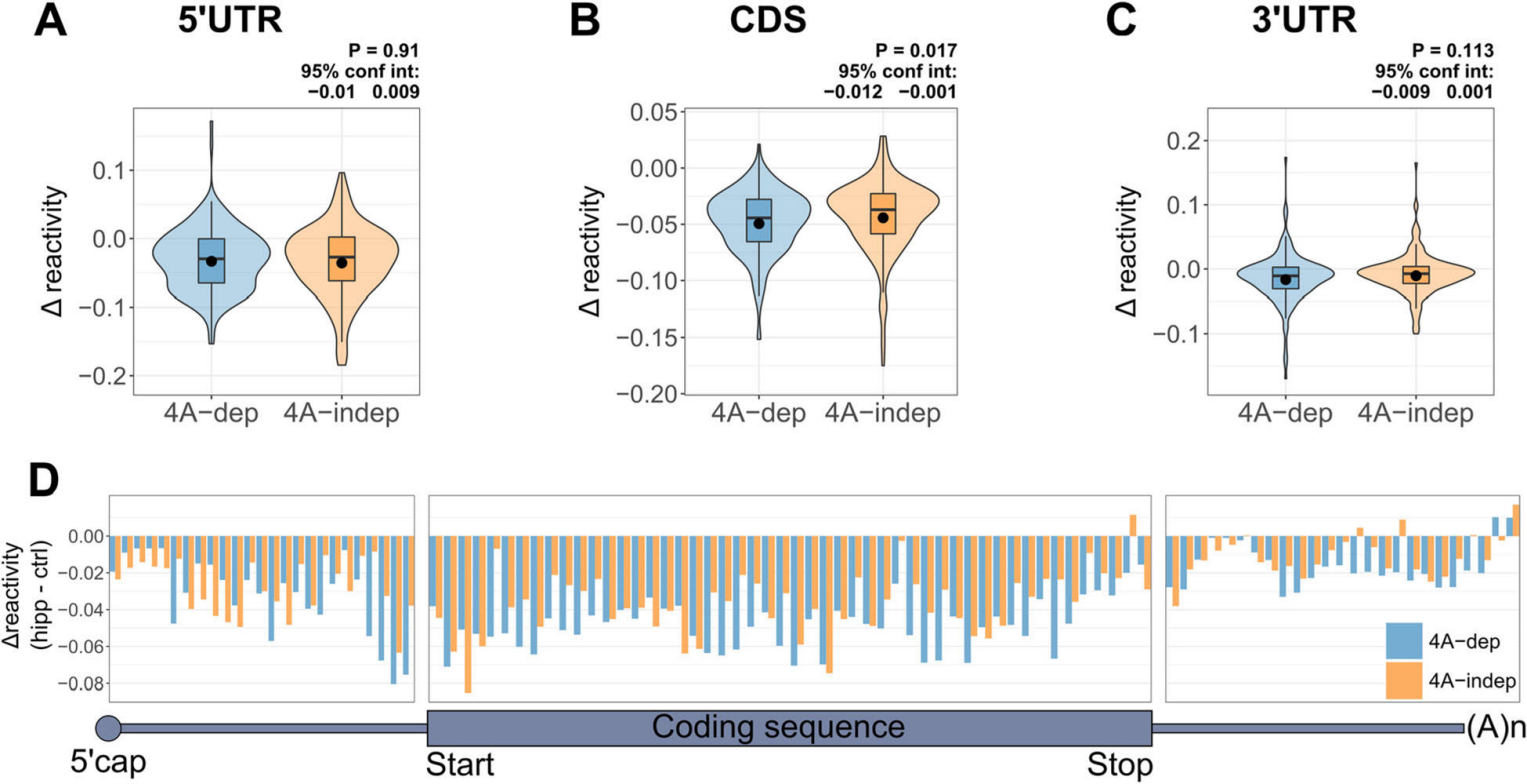
eIF4A-dependent mRNAs become more structured than eIF4A-independent mRNAs at the 3′ end of the 5′UTR. **a**–**c** Violin plots for the average Δ reactivity in the 5′UTR, CDS, and 3′UTR for eIF4A-dependent (4A-dep) and independent transcripts (4A-indep). Transcripts were filtered by coverage and 5′ end coverage and the most abundant transcript per gene was selected, resulting in 192 4A-dep mRNAs. To ensure equal group sizes, of the 663 4A-indep transcripts, the 192 transcripts with the lowest posterior probability were selected, i.e., the 192 mRNAs that with greatest confidence are 4A-indep. Violin plots include boxplots, with the mean denoted by a dot. *P* values and 95% confidence intervals were calculated using an un-paired, two-sided Wilcoxon test. **d** Binned Δ reactivity for all 4A-dep (blue) and 4A-indep (orange) mRNAs across the length of the UTRs (25 bins) and coding sequence (50 bins). Negative values indicate increased structure following hippuristanol treatment, whereas positive values indicate decreased structure. Only those mRNAs from panels **a**–**c** whose 5′UTR, CDS, and 3′UTR are at least 100 nt were included, resulting in 147 4A-dep mRNAs and an equal group size of 4A-indep mRNAs

To compare RNA structural changes in 4A-dep and 4A-indep mRNAs following eIF4A inhibition, we plotted the average Δ reactivities of these groups of transcripts (Fig. 4a–c). To our surprise, there was no significant difference in the Δ reactivity between 4A-dep and 4A-indep 5′UTRs (Fig. 4a). There was also no significant difference in the change in MFE and strandedness of folded 5′UTRs following hipp treatment, between 4A-dep and 4A-indep mRNAs (Additional file 1: Figure S7B-C). There is a small, yet statistically significant difference in the average Δ reactivity between 4A-dep and 4A-indep CDSs (Fig. 4b) but not 3′UTRs (Fig. 4c).

As the largest structural changes in the 5′UTR are occurring close to the CDS, we next plotted the binned Δ reactivity across the transcript for our 4A-dep and 4A-indep mRNAs (Fig. 4d). This clearly shows that following hipp treatment, 4A-dep mRNAs gain in structure the most just upstream of the CDS and that this is the region in which we see the biggest difference in Δ reactivity between 4A-dep and 4A-indep mRNAs. Upon examination of the final 60 nt of the 5′UTR, it seems that the biggest differences in Δ reactivity between the 4A-dep and 4A-indep mRNAs are within the last 20 nt of the 5′UTR (Additional file 1: Figure S7D). Interestingly, this is the same region wherein the translationally repressed mRNAs are more structured than the efficiently translated mRNAs under control conditions (Additional file 1: Figure S6A), suggesting that increased structure within this region following eIF4A inhibition is most inhibitory to translation. There was no significant difference in uTIS scores between 4A-dep and 4A-indep mRNAs (Additional file 1: Figure S7E), or between the high-sensitivity (4A-dep) and low-sensitivity (4A-indep) mRNAs from Iwasaki et al. [33], following hipp treatment in HEK293 cells (Additional file 1: Figure S7F). These results indicate no enrichment of upstream translation in 4A-dep mRNAs, eliminating the possibility that the increased structure just upstream of the CDS in 4A-dep mRNAs is due to reduced ribosome occupancy in uORFs.

On balance, we interpret these findings as evidence that the region immediately upstream of the start codon confers eIF4A dependence upon mRNAs for their efficient translation. If these mRNAs were refolding due to translational inactivity when eIF4A is inhibited, resulting in reduced binding of the 48S initiation complex at the start codon, then we would also expect 4A-dep mRNAs to gain more structure than 4A-indep mRNAs immediately downstream of the start codon within the CDS, which is not observed (Additional file 1: Figure S7D).

### eIF4A-dependent 5′UTRs gain in localized structure more than eIF4A-independent 5′UTRs upon hippuristanol treatment

**Fig. 5:**
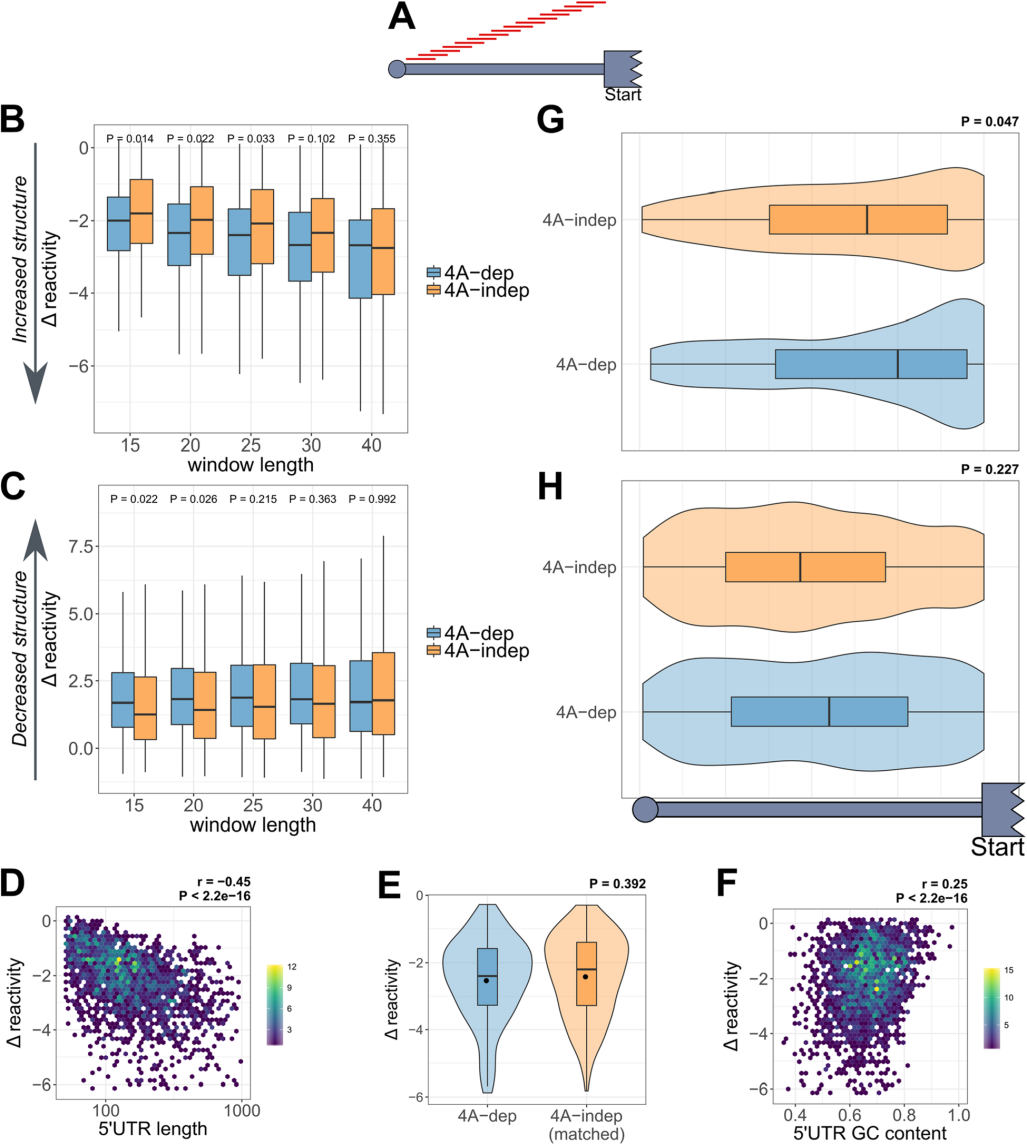
Increased length of eIF4A-dependent 5′UTRs drives increased localized structure potential, which confers increased dependency on eIF4A activity when positioned close to the coding sequence. **a** Diagrammatic representation of the sliding window approach used in this figure. First, the Δ reactivity is calculated for every possible window, after setting the width of the window and the step. Then, these windows can be filtered by certain criteria. **b**, **c** Boxplots depicting the Δ reactivity of the windows with the **b** biggest decrease or **c** biggest increase Δ reactivity per 5′UTR for eIF4A-dependent (4A-dep) and eIF4A-independent (4A-indep) mRNAs with different sized windows. *P* values were calculated by an un-paired, two-sided Wilcoxon test, without correcting for multiple comparisons. **d** Density scatter plot depicting the correlation between the Δ reactivity of each 20-nt window which gained in structure the most per transcript against its 5′UTR length. *P* value and the *r* correlation coefficient were calculated by a Pearson correlation test. The number of transcripts within each hexagon is denoted in the legend. **e** Δ reactivity of the 20-nt windows with the biggest decrease in Δ reactivity per 5′UTR for 4A-dep and a 4A-indep group that is matched by 5′UTR length. *P* value is calculated by an unpaired two-sided Wilcoxon test. **f** Density scatter plot depicting the correlation between the Δ reactivity of each 20-nt window which gained in structure the most per transcript against its GC content. *P* value and the *r* correlation coefficient were calculated by a Pearson correlation test. The number of transcripts within each hexagon is denoted in the legend. **g**, **h** Violin plots showing the binned positions within the 5′UTRs of the 20-nt windows which **g** increase in structure the most per 5′UTR, and **h** decrease in structure the most per 5′UTR for 4A-dep and 4A-indep mRNAs. *P* value is calculated by an un-paired, two-sided Wilcoxon test

To identify the regions that changed in DMS reactivity the most within each 5′UTR, we carried out a sliding window analysis. This approach measures the Δ reactivity of every possible sequence of a given length (Fig. 5a) and identifies the window with the biggest decrease or increase in reactivity per transcript. Figure 5b and c show the Δ reactivities of these windows within 4A-dep and 4A-indep 5′UTRs, with varying window sizes. Interestingly, the Δ reactivity of the windows that decrease in reactivity the most in the presence of hipp is more negative for 4A-dep mRNAs, suggesting that these 5′UTRs gain more in localized structure following eIF4A inhibition. Furthermore, this difference is most statistically significant with windows of 15 nt (Fig. 5b), indicating perhaps the optimal length of secondary structure which eIF4A can efficiently unwind within the 5′UTRs of cellular mRNAs. Interestingly, this is in rough agreement with the hairpin size with which eIF4A has been shown to efficiently unwind in vitro [47], and also the translocation step size of eIF4A in single molecule experiments [48]. The Δ reactivity of the windows that are increasing in reactivity the most, i.e., losing structure with eIF4A inhibition, mirrors the pattern we see for the windows that decrease in reactivity, in that they are increasing in reactivity more for 4A-dep 5′UTRs (Fig. 5c). This explains why there is no difference in the average Δ reactivity across the whole 5′UTR between 4A-dep and 4A-indep 5′UTRs, as certain regions gain in structure, but adjacent regions lose structure. This suggests that following eIF4A inhibition, 5′UTRs are remodeled, undergoing local gains and losses in structure that tend to balance out, rather than gaining in structure throughout. 4A-dep mRNAs are seen to contain more stable localized secondary structures than 4A-indep mRNAs, and we propose that it is these small localized elements that are inhibitory to scanning.

### Increased length of eIF4A-dependent 5′UTRs drives increased localized structure potential

One possible explanation for eIF4A-dependent 5′UTRs gaining more in localized structure could be that 4A-dep 5′UTRs are longer (Fig. 3b), therefore increasing the number of potential intra-molecular RNA interactions and as a result the likelihood of stable local secondary structures forming. We therefore tested for a correlation between the extent of localized gains in structure and 5′UTR length by plotting the most negative Δ reactivity per transcript against its 5′UTR length. Figure 5d shows that there is indeed a strong negative correlation, indicating that the longer the 5′UTR, the more likely it is to have a region that gains in stable secondary structure. To evaluate whether the increased localized structure in 4A-dep 5′UTRs is caused by their increased length, we created a 4A-indep group that was matched by 5′UTR length. Interestingly, there was no significant difference in Δ reactivity between this matched 4A-indep group and 4A-dep mRNAs (Fig. 5e), suggesting that 4A-dep mRNAs gain in localized secondary more than 4A-indep mRNAs because of increased 5′UTR length, which likely explains why 4A-dep mRNAs possess longer 5′UTRs. There was not a strong correlation between 5′UTR GC content and increased localized structure (Fig. 5f).

To assess for any sequence specificity within the regions that are gaining the most in structure following hipp treatment, we carried out motif discovery using MEME [49] on the 20-nt windows that decrease in reactivity the most. However, this did not generate any significantly enriched motifs.

### Localized structures confer increased eIF4A dependence only when positioned at the 3′ end of the 5′UTR

The sliding window analysis suggests that 4A-dep mRNAs have increased localized secondary structure compared to 4A-indep RNAs, and that this is at least partially explained by their having longer 5′UTRs. However, there remain many 4A-indep mRNAs with long 5′UTRs, which also increase in localized secondary structure to a similar extent following eIF4A inhibition (Fig. 5e). We therefore sought to address why these mRNAs remain insensitive to eIF4A inhibition. We hypothesized that, based on the pattern of reactivity changes shown in Fig. 4d, the position of these localized gains in 5′UTR structure is important in determining sensitivity to eIF4A inhibition. We therefore plotted the relative positions of these windows within the 5′UTRs of 4A-dep mRNAs and the 4A-indep group which has been matched by 5′UTR length, which we know have similar average Δ reactivities (Fig. 5e). For 4A-dep mRNAs, we see a much stronger bias in the position of these windows towards the 3′ end of the 5′UTR than in 4A-indep mRNAs (Fig. 5g), whereas importantly for the windows that lose structure, there is no positional bias for either the 4A-dep or 4A-indep mRNAs (Fig. 5h). This therefore suggests that increased structure just upstream of the CDS is most inhibitory to translation following eIF4A inhibition.

**Fig. 6:**
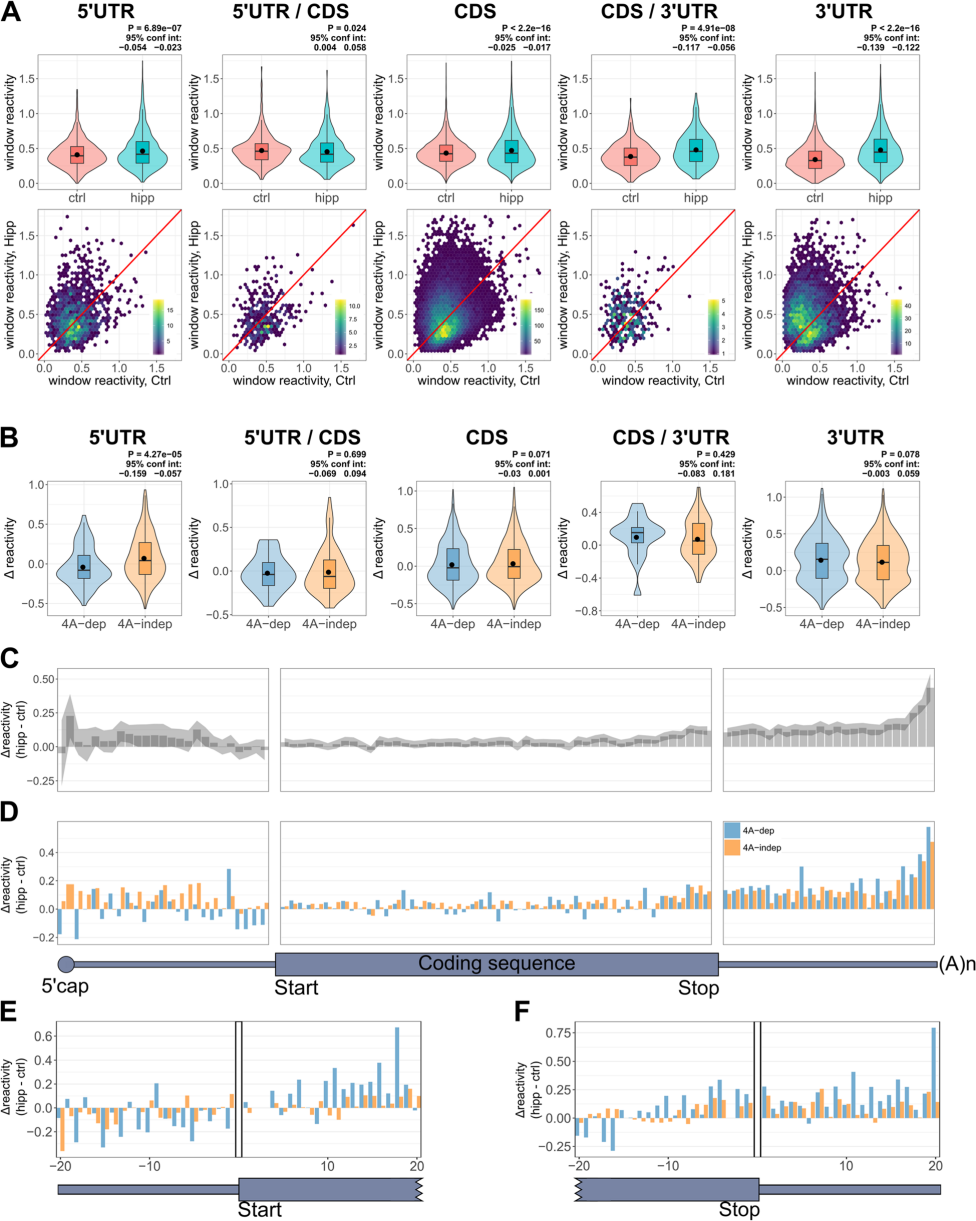
Differentially reactive windows gain in structure in 4A-dep 5′UTRs more than in 4A-indep 5′UTRs. **a** Violin and density scatter plots depicting the average reactivity under control and hippuristanol conditions within all 27,396 windows identified by dStruct with an FDR less than 0.25. There are 1467 windows in the 5′UTRs, 372 windows overlapping the 5′UTR/CDS junction, 19,269 windows in the CDS, 331 windows overlapping the CDS/3′UTR junction and 5957 windows in the 3′UTR. Violin plots include boxplots, with the mean denoted by a dot. *P* values and 95% confidence intervals were calculated using a paired, two-sided Wilcoxon test. **b** Violin plots depicting the Δ reactivities of all windows in 4A-dep and 4A-indep mRNAs. There are 87 and 379 windows in 4A-dep and 4A-indep 5′UTRs respectively, 37 and 125 in 4A-dep and 4A-indep 5′UTR/CDS junctions respectively, 292 and 1508 in 4A-dep and 4A-indep CDSs respectively, 19 and 115 in 4A-dep and 4A-indep CDS/3′UTR junctions respectively and 183 and 924 in 4A-dep and 4A-indep 3′UTRs respectively. *P* values and 95% confidence intervals were calculated using an un-paired, two-sided Wilcoxon test. **c**, **d** Binned average Δ reactivity of **c** all dStruct windows and **d** all windows in 4A-dep and 4A-indep mRNAs. Shaded area in **c** represents 95% confidence limits for the difference in means between control and hippuristanol mRNAs within each bin, calculated by a paired two-sided *t* test. **e**, **f** Average Δ reactivity for each nucleotide either side of **e** the 5′UTR/CDS junction and **f** the CDS/3′UTR junction, for all windows spanning these junctions in 4A-dep and 4A-indep mRNAs

As our findings till now have relied on averaged reactivities between the three replicates, the information within the biological variation is lost. We therefore sought to validate our findings using the dStruct package [50], which identifies differentially reactive regions that differ more in their pattern of reactivity between control and treated samples, than between replicates. As dStruct takes variability between replicates into account, we reduced the coverage threshold to include all transcripts with a combined coverage more than one for all replicates in each condition, thereby including less abundant transcripts into the analysis. We used whole transcripts, rather than spliced regions, so that dStruct could also identify windows that overlap UTR/CDS boundaries. dStruct first identifies windows that appear more similar within replicates than conditions, before applying the Wilcoxon signed-rank test, controlling for false discovery rates (FDRs) using the Benjamini-Hochberg procedure [50]. The FDRs are shown in Additional file 1: Figure S8A, and we used a cutoff of 0.25, which identified 27,396 differentially reactive windows within 4087 transcripts. We then assigned each window into one of five groups, depending on whether they were in the 5′UTR, CDS, or 3′UTR or whether they overlapped either UTR/CDS junction. The lengths of the windows from each group are shown in Additional file 1: Figure S8B. This is in agreement with the optimal length of the windows with the biggest decrease in reactivity from the sliding window analysis in Fig. 5b, in that the most common window length is 15 nt and the median is 21 nt in the 5′UTR. The reactivities under control and hipp conditions for all windows are shown in Fig. 6a, and the Δ reactivities of those windows in 4A-dep and 4A-indep mRNAs are shown in Fig. 6b. The reactivity of the windows in the 3′UTRs and the 3′UTR/CDS junction are changing the most, with a relatively large increase in reactivity following eIF4A inhibition (Fig. 6a). This could indicate reduced protein binding following translational repression with hipp. We also see a slight, yet statistically significant increase in the reactivity of the differentially reactive windows in the 5′UTR and CDS (Fig. 6a). This is slightly surprising given that the average reactivity across the entire lengths of these regions is decreasing following hipp treatment (Additional file 1: Figure S4A-C). This therefore suggests that while overall, reactivity is decreasing in these regions, the average reactivity in the differentially reactive windows is actually increasing. Crucially however, when we compare the Δ reactivity between the differentially reactive windows within the 5′UTRs of 4A-dep and 4A-indep mRNAs, the majority of the windows from 4A-dep 5′UTRs are decreasing in reactivity following hipp treatment and these are significantly more negative than those windows from 4A-indep 5′UTRs, which is not seen in any of the other regions (Fig. 6b). The larger decrease in reactivity observed in 4A-dep 5′UTRs following eIF4A inhibition suggests that these differentially reactive windows are gaining in structure more in the 5′UTRs of 4A-dep mRNAs compared to 4A-indep mRNAs.

To determine whether this analysis also indicated increased structure following eIF4A inhibition at the 3′ end of 5′UTRs, we binned all the windows across the length of the transcript (Fig. 6c) and also just those windows from 4A-dep and 4A-indep mRNAs (Fig. 6d). Crucially, we again see that the biggest difference between 4A-dep and 4A-indep mRNAs to be just upstream of the coding region, with 4A-dep mRNAs gaining more in structure in this region (Fig. 6d). We should note however that we now also see a difference between these mRNAs at the very 5′ of the 5′UTR, which we did not see in our prior analysis (Fig. 4d), which could also indicate increased unwinding of secondary structure by eIF4A in cap proximal regions.

One alternative explanation for our data is that we see increased structure following hippuristanol treatment just upstream of the coding region more in 4A-dep mRNAs, due to reduced ribosome occupancy over the translation start site, during the transition of the 48S initiation complex into the elongation competent 80S complex. However, if this was true, we would also expect there to be increased structure immediately downstream of the start site, which we do not see (Additional file 1: Figure S7D). To confirm this finding, we plotted the Δ reactivity of all dStruct windows that overlap the 5′UTR/CDS junction (Fig. 6e). Again we observe decreased reactivity in 4A-dep transcripts compared to 4A-indep just prior to the start site, but actually increased reactivity just downstream of the start site, supporting our previous conclusions. There was no obvious difference in reactivity patterns between 4A-dep and 4A-indep mRNAs at the CDS/3′UTR junction (Fig. 6f).

We again used MEME [49] to search for any enriched sequences in the windows identified by dStruct in the 5′UTRs of 4A-dep mRNAs, but this did not return any enriched motifs.

The above findings therefore support the following conclusions. Firstly, following eIF4A inhibition, 5′UTRs are remodeled, gaining structure in certain regions and losing it elsewhere. The extent to which 5′UTRs are remodeled is strongly affected by 5′UTR length, with longer 5′UTRs gaining more in localized structure (Fig. 5d). This likely explains why 4A-dep 5′UTRs tend to be longer (Fig. 3b), as this will increase the likelihood of stable localized structure formation. However, increased localized structure alone does not seem to accurately predict eIF4A-dependency as a 5′UTR length-matched 4A-indep group of mRNAs gained in local structure to a similar extent as 4A-dep messages (Fig. 5e). Crucially, in 4A-dep mRNAs, these influential highly structured elements are located predominantly at the 3′ end of the 5′UTR (Figs. 4d and 6d and Additional file 1: Figure S7D). Fitting with our findings that translationally repressed mRNAs are more structured in this region under control conditions (Fig. 2e) and that 5′UTRs are generally more structured at their 5′ ends (Fig. 1c), i.e., away from the CDS, it is therefore those mRNAs that gain the most structure just upstream of the coding region following eIF4A inhibition that are the most translationally repressed.

## Discussion

It is widely accepted that eIF4A is required for both ribosome recruitment and scanning, and it has been assumed that this requirement is due to the helicase activity of eIF4A [13, 14]. Attempts to understand how eIF4A and secondary structure dictate translation efficiency have been limited to single 5′UTR examples [51–54], and these investigations have focused on cap-proximal structures, due in part to eIF4A being a component of the cap binding complex eIF4F. Recent studies in both yeast and mammalian systems have shown that eIF4A enhances ribosome recruitment regardless of RNA structural complexity [16, 17]. This could explain why in yeast, eIF4A is thought to be required globally for the translation of all cellular mRNAs, with Ded1p acting as the main helicase involved in unwinding secondary structures distal from the 5′ cap [55]. However, given that the mRNAs most sensitive to eIF4A inhibition in human cells have longer, more GC-rich 5′UTRs [4, 11, 19], it would be surprising if eIF4A activity was restricted to the cap proximal region. Here we take a global and unbiased approach to probe the roles of eIF4A in translation initiation, with the use of mRNA structure profiling in a human cell line, through the modification of single-stranded adenines and cytosines by DMS.

Our data suggest that elevated eIF4A in human cells is required to unwind secondary structures to aid scanning of the small ribosomal subunit on mRNAs with particularly long and structured 5′UTRs. This could therefore explain why certain mRNAs are more dependent on eIF4A activity than others. It is clear that eIF4A is also required for ribosome recruitment, with recent evidence suggesting a helicase independent role of eIF4A during this step, possibly by remodeling the conformation of the 40S subunit [16, 17]. The absence of major cap proximal structural changes in 4A-dep mRNAs (Fig. 4d) is consistent with a model in which the requirement of mRNAs for eIF4A during ribosome recruitment is equal, as has been suggested previously [16, 17]. It appears that the role of the helicase activity of eIF4A in human cells is more similar to that of Ded1p in yeast, in that mRNAs most dependent on Ded1p, and its paralogue Dbp1, contain longer 5′UTRs with increased propensity for secondary structures [55, 56]. While Ded1p appears to act in a cooperative manner with the eIF4F complex to promote 48S initiation complex assembly in yeast [57, 58], the exact role of the human orthologue of Ded1p, named DDX3, is less clear. DDX3 has been implicated in many aspects of RNA metabolism, including translation [59], where it is thought to unwind cap proximal structures to allow ribosome recruitment in a mRNA-specific manner [60]. Both the sliding window (Fig. 5b) and dStruct analysis (Additional file 1: Figure S8B) support the in vitro data that eIF4A can only efficiently unwind hairpins up to roughly 15–20 nt [47, 48]. DHX29 has been implicated in unwinding more stable hairpin structures [61, 62], which would therefore be consistent with our data.

To confidently measure DMS reactivity, it is essential that the reference transcriptome used for the bioinformatic analysis is a true representation of the cellular transcriptome. For example, within MCF7 cells, roughly 30% of expressed mRNAs possess a 5′UTR less than half the length of that annotated in the RefSeq database [35]. Our data support this finding (Additional file 1: Figure S3), highlighting an important and underappreciated potential problem for transcriptome-wide structure-probing studies. Mapping our data to the RefSeq transcriptome would have resulted in an absence of reads, and therefore an absence of reverse transcriptase stops, in the 5′UTR regions included in the RefSeq database, but not actually present in MCF7 cells. These regions would therefore appear as highly protected and thus highly structured had the RefSeq database been utilized. Furthermore, they would appear equally protected in both control and hipp-treated samples, which would therefore be wrongly interpreted as being equally structured under both conditions.

Currently, it is unclear whether hipp acts equally to repress eIF4A within the eIF4F complex or free eIF4A. Given that we chose the IC_50_ concentration of hipp, and that cellular levels of eIF4A are roughly ten times higher than the eIF4F complex [18], it is possible that we are predominantly targeting one of these populations of eIF4A, which could have important implications for the interpretations of this data.

One explanation for the positional bias of increased localized structures in the 5′UTRs of eIF4A-dependent mRNAs is that structures involving sequence elements on both sides of the 5′UTR and CDS junction could be the most highly repressive to translation. Indeed, a recent study using a reconstituted system purified from yeast found that structures on both sides of the start codon were synergistically repressive to ribosome recruitment [17]. However, the lack of increased structure in 4A-dep mRNAs following eIF4A inhibition immediately 3′ of the start codon (Fig. 6e and Additional file 1: Figure S7D) would not be consistent with this.

## Conclusions

Our structural data support a model in which the helicase activity of eIF4A is required throughout the 5′UTR during scanning. The lack of structural changes at the extreme 5′ end of the 5′UTR is consistent with a global helicase-independent role of eIF4A in ribosome recruitment. We find that localized eIF4A-mediated unwinding of 5′UTR structure is accompanied by the compensatory folding of alternative structures elsewhere in the region. Crucially, however, following eIF4A inhibition the greatest increases in structure occur just upstream of the CDS (Fig. 1c). We show that the increased length of 5′UTRs seen in eIF4A-dependent mRNAs is associated with larger localized gains in structure following eIF4A inhibition, but it is only when these structural elements are located adjacent to the CDS that they confer greater dependence on eIF4A activity (Figs. 4d and 5g). This is further supported by the observation that highly translated mRNAs are less structured than translationally repressed mRNAs in this same region (Fig. 2e), and we eliminate the possibility that these observations are due to translation elongation through uORFs (Additional file 1: Figure S5G-H, S6B and S7E-F). We also demonstrate that the pattern of reactivity changes we observe following hipp treatment are not caused by reduced eIF4A binding (Fig. 1d), and we eliminate the possibility that the ribosome could protect from DMS reactivity (Fig. 1e).

In summary, upon globally mapping changes in RNA structure following eIF4A inhibition, we find that 5′UTRs are generally remodeled, with eIF4A-dependent mRNAs gaining most in localized structure just upstream of the CDS. We propose that increased structure potential at the 3′ end of the 5′UTR is a key determinant of preferential gene expression in conditions of elevated eIF4A activity as seen in cancer cells [4].

## Methods

### Cell culture

MCF7 cells were grown in DMEM, high glucose, GlutaMAX Supplement, pyruvate (ThermoFisher 31966-021), supplemented with 10% FCS. Cells were tested regularly for mycoplasma and were authenticated by Eurofins using PCR-single-locus-technology.

### 35S Protein labeling

2.25 × 10^5^ MCF7 cells were seeded in 12-well plates overnight. Medium was replaced with DMEM lacking methionine and cysteine (ThermoFisher 21013024), supplemented with 1% glutamine and 10% FCS with the relevant concentration of hippuristanol. After 30 min, 4.5 μl EasyTag Express ^35^S Protein Labeling Mix (11 mCi/ml) (PerkinElmer NEG772002MC) was added to each well and incubated for a further 30 min at 37 °C. Cells were washed twice with ice-cold PBS and lysed in the wells with 200 μl passive lysis buffer for 5 min on ice. Cells were scraped and pipetted into 1.5-ml centrifuge tubes. Lysate was centrifuged at 12,000*g* for 5 min and 160 μl supernatant pipetted into new tube. Ten microliters was used in Bradford assay to determine protein concentration, and the remaining 150 μl was precipitated with 150 μl 25% TCA on ice for 30 min. The precipitated lysate was loaded onto glass fiber Whatmann filters, pre-wetted with 500 μl 25% TCA, in a vacuum manifold, and dried by vacuum. Filters were washed twice with ice-cold 70% IMS and twice with ice-cold acetone, before thoroughly drying. Filters were placed into scintillation vials with the addition of 10 ml scintillation cocktail and counts per minute (cpm) measured using a scintillation counter. cpm were normalized by protein concentration.

### DMS treatment

To ensure dimethyl sulphate (DMS) treatment was carried out under single hit kinetics, a range of concentrations of DMS was tested as in [63] (data not shown). Note that DMS is extremely toxic and all work should be carried out under appropriate safety measures [63].

Fifteen-centimeter plates with 70–80% confluent MCF7 cells were treated with 150 nM hippuristanol or an equal concentration of DMSO (0.07%) for 1 h by replacing the medium. Medium was then replaced with PBS with or without 50 mM DMS for 10 min. Cells were washed once with PBS containing 250 mM DTT, to quench the DMS, followed by extraction of the RNA with TRIzol (ThermoFisher 15596026) as per the manufacturer’s instructions and isopropanol precipitation. As poly(A) selection of RNA is sensitive to salt, RNA was then ethanol precipitated with 500 mM ammonium acetate. Integrity of RNA was checked on an Agilent 2100 Bioanalyzer with the Eukaryote Total RNA Nano assay, and RIN scores of 10 were obtained for every sample. To ensure single hit kinetics, 2 μg total RNA was used in a reverse transcription reaction using a 5′ Cy5 labeled primer, specific for the human 18S rRNA: 5′CCAAAGGAACCATAACTGATT3′ and the resulting cDNA run on a sequencing gel (Additional file 1: Figure S1D). Three biological replicates were obtained for each sample.

### Structure-seq2 library preparation

Library preparation was essentially carried out as in [29] (Additional file 1: Figure S1E) with details below.

#### Poly(A) selection

One hundred twenty-microgram total RNA per sample was subjected to two rounds of poly(A) selection with the Poly(A) Purist MAG Kit (AM1922), as per the manufacturer’s instructions. Poly(A) RNA was dissolved in 17 μl TE (10 mM Tris-HCl pH 7.5, 1 mM EDTA). One microliter was used to run on an Agilent 2100 Bioanalyzer with the mRNA Nano assay to confirm removal of rRNA, and 1 μl was used to determine RNA concentration with a nanodrop. The remaining 15 μl (typically slightly more than 1 μg) was used in the following reverse transcription step.

#### Reverse transcription

For each sample, 1 μg of poly(A) RNA was diluted to 15 μl and mixed with 2 μl of the N6 linker oligo: 5′CAGACGTGTGCTCTTCCGATCNNNNNN3′ (100 μM) and 3 μl KCl (1 M) and split between 2 × 10 μl. RNA was denatured in a thermal cycler at 90 °C for 1 min before rapidly cooling to 4 °C and held for 1 min. Temperature was increased to 25 °C, and 4 μl 5X buffer (100 mM Tris-HCl pH 8.4, 25 mM MgCl_2_, 25 mM DTT, 2.5 mM dNTPs), 5 μl nuclease free water, and 1 μl SuperScript III (200 U/μl) (Thermo Fisher 18080085) were added to each 10-μl sample. Samples were incubated for 5 min at 25 °C to promote annealing and to allow slight extension of RT primers, followed by 5 min at 42 °C for further extension and finally 55 °C for 50 min for full extension. Samples were then heated at 85 °C for 5 min to denature the enzyme, followed by addition of 2 μl NaOH (1 M) and incubation at 95 °C for 10 min to hydrolyze the RNA. Samples were purified by gel extraction with an 8% polyacrylamide, 1-mm-thick denaturing gel (see Gel Extraction). To ensure maximal removal of the N6 linker, which can form an unwanted byproduct if not removed, cDNA running above an N78 ssDNA oligo was purified, which should run 50 nt higher than the N6 linker. cDNA was dissolved in 5.5 μl Tris-HCl (pH 8.0).

#### Ligation

To the 5.5 μl cDNA was added 0.5 μl hairpin donor oligo /5Phos/TGAAGAGCCTAGTCGCTGTTCANNNNNNCTGCCCATAGAG/3SpC3/ (400 μM), 2 μl betaine (5 M), and 8 μl 50% PEG 8000 (added last and at room temperature to avoid precipitating DNA). Samples were heated at 95 °C for 90 s and allowed to cool slowly to room temperature. Two microliter 10X T4 DNA ligase buffer and 2 μl T4 DNA ligase (400 U/μl) (NEB M0202S) were added, and the samples were incubated at 16 °C for 6 h followed by 30 °C for 6 h and then 65 °C for 10 min to denature the enzyme. Samples were purified by gel extraction with a 6% polyacrylamide, 1-mm-thick denaturing gel (see Gel Extraction) and cDNA running above an N118 ssDNA oligo was purified; this oligo should run 50 nt above any ligated N6 linker. Ligated cDNA was dissolved in 18 μl Tris-HCl (pH 8.0).

#### PCR

To determine the number of PCR cycles required, 25 μl reactions were set up with 5 μl taken from the samples at cycles 11, 14, 17, 20, and 23 and the amplified DNA was run on a 5% polyacrylamide denaturing gel. Reactions were set up with 5 μl 5X Q5 buffer, 5 μl GC rich enhancer buffer, 0.5 μl dNTPs (10 mM each), 0.25 μl Q5 Hot Start High-Fidelity DNA Polymerase (2000 U/ml) (NEB M0493 L), 1 μl Truseq forward primer: 5′AATGATACGGCGACCACCGAGATCTACACTCTTTCCCTACACGACGCTCTTCCGATCTTGAACAGCGACTAGGCTCTTCA3′ (10 μM), 1 μl relevant Truseq reverse primer: 5′CAAGCAGAAGACGGCATACGAGATBARCODEGTGACTGGAGTTCAGACGTGTGCTCTTCCGATC3′ (10 μM), 4.5 μl ligated cDNA, and 7.75 water.

**Table:** 

Sample	Repeat	BARCODE
Control/DMS(−)	A	5′CGTGAT3′
Control/DMS(+)	A	5′ACATCG3′
Hippuristanol/DMS(−)	A	5′GCCTAA3′
Hippuristanol/DMS(+)	A	5′TGGTCA3′
Control/DMS(−)	B	5′CACTGT3′
Control/DMS(+)	B	5′ATTGGC3′
Hippuristanol/DMS(−)	B	5′GATCTG3′
Hippuristanol/DMS(+)	B	5′TCAAGT3′
Control/DMS(−)	C	5′CTGATC3′
Control/DMS(+)	C	5′AAGCTA3′
Hippuristanol/DMS(−)	C	5′GTAGCC3′
Hippuristanol/DMS(+)	C	5′TACAAG3′

Reactions were activated at 98 °C for 30 s followed by cycling between 98 °C for 10 s and 72 °C for 45 s. It was determined that 17 cycles was optimal, as this was the first cycle where the product was visible on the gel. The remaining samples were amplified in 3 × 25 μl reactions as above, for 17 cycles plus a final extension time of 5 min at 72 °C, and then combined and gel purified on a 5% polyacrylamide, 1.5-mm-thick denaturing gel. Resultant PCR products running between ~ 200 and 600 nt, as determined with ss50 ladder (Simplex Sciences), were purified and dissolved in 20 μl Tris-HCl (pH 8.0). Samples were run on an Agilent 2100 Bioanalyzer with the high sensitivity DNA assay to ensure the size of the libraries was as expected with minimal by-product contamination.

#### Gel extraction

An equal volume of 2X loading buffer (95% formamide, 20 mM Tris HCl (pH 7.5), 20 mM EDTA, 0.025% bromophenol blue and xylene cyanol) was added, and DNA was denatured at 98 °C for 5 min prior to loading onto a 8.3 M urea polyacrylamide gel (22 cm long, pre-run for 2 h at 18 W so that the temperature of gel was between 50 and 60 °C). The gel was run at 18 W for 2 h for the post-RT and post-ligation gels and until the xylene cyanol was close to the bottom for the post-PCR gel. Following the run, the gel was carefully placed onto a piece of Saran wrap and stained with 50 ml 1X TE, 1X SybrGold (S11494) for 10 min in a plastic tray, wrapped in aluminium foil. The staining solution was removed, and another layer of Saran wrap was placed on top of the gel, and the DNA was visualized on a Safe Imager 2.0 Blue Light Transilluminator. The region to cut was drawn on the Saran wrap with a marker pen. The gel was then cut with a clean razor blade and placed in a 5-ml DNA LoBind Eppendorf tube. To break the gel into tiny pieces, a needle was used to make a hole in the bottom of the tube and the gel was forced through the hole into another 5-ml tube by centrifugation at 6000*g* for 5 min. Three milliliter TEN_250_ (1X TE, 250 mM NaCl) was then added and pipetted into a 50-ml DNA LoBind Eppendorf tube. Another 3 ml TEN_250_ was added, and the slurry was incubated in a shaking incubator at 220 rpm at 37 °C for at least 24 h. This crush and soak method was found to be essential for sufficient extraction of the DNA from the gel.

Following incubation, the slurry was briefly spun down and as much of the liquid was pipetted off and filtered through Spin-X Centrifuge Tube Filters (0.22 μm Pore CA Membrane). Samples were then precipitated with the addition of 1 μl GlycoBlue and an equal volume of isopropanol overnight at room temperature in 5 ml DNA LoBind tubes. The DNA was pelleted by centrifugation at 12,000*g* and washed twice with 70% ethanol and dissolved in Tris-HCl pH 8.0.

### Sequencing and bioinformatic pipeline

Libraries were sequenced by the DNA Sequencing Facility at the Dept of Biochemistry, University of Cambridge, on a NextSeq 500. Concentrations of final libraries were determined by the facility using qPCR, and equal concentrations of each sample were pooled together and sequenced on three high output runs of 150 cycles, single-ended. A custom sequencing primer was used: 5′TCTTCCGATCTTGAACAGCGACTAGGCTCTTCA3′ to avoid low diversity at the start of the sequencing run, resulting in the nucleotide directly adjacent to the DMS-modified nucleotide being the first nucleotide sequenced. 1,268,740,434 reads were obtained in total. The raw sequencing reads are available at the Gene Expression Omnibus (GEO) database accession GSE134865 in fastq format.

Sequencing reads were processed and analyzed using the StructureFold2 bioinformatic pipeline [34].

The fastq_trimmer.py script was used to remove 5′ and 3′ adapters, to trim bases from the 3′ end with a NextSeq quality score below 30 and to remove any reads that were less than 20 nt after trimming. This script uses cutadapt (version 1.14) [64]. Ninety-nine percent of reads passed the filtering following trimming.

The fastq_mapper.py script was used to map all trimmed reads to the MCF7-specific transcriptome (see below) using bowtie2 (version 2.3.2) [65]. A summary of mapped reads is in Additional file 1: Table S1. Overall, 89.1% of reads mapped to the transcriptome, of which 86.1% mapped to more than one location and 13.9% mapped uniquely. The high percentage of multi-mapped reads is primarily due to transcript variants, as mapping to a transcriptome created by selecting the longest transcript per gene resulted in 75.4% reads mapping, of which only 20.8% were multi-maps. We therefore allowed multi-mapped reads and all downstream analysis was carried out at the gene level by selecting the most abundant transcript per gene, based on our RNA-Seq data. Although we cannot rule out that some genes with several abundant isoforms may have different folds, this is unlikely to affect the results, especially when looking at reactivity in the 5′UTRs, as the sequence of these regions is less often altered between splice variants, compared to CDSs and 3′UTRs. To test how many genes had more than one relatively abundant transcript, we assessed the percentage of reads predicted to map to the most abundant transcript per gene, based on our total RNA-Seq data. Of the 1266 genes analyzed in Fig. 1c, 391 genes have more than one splice variant. For 75% of these 391 genes, 67.8% of the reads that map to that gene are predicted to arise from the most abundant transcript, and for 25% of these genes, 98.2% of the reads are predicted to arise from the most abundant transcript.

The sam_filter.py script was used to filter any mapped reads that contain more than 4 mismatches or that have a mismatch at position 1, which could have resulted from the addition of a random nt to the 3′ end of the cDNA prior to ligation. Sixty-four percent, 74%, 64%, and 73% of reads were retained after filtering for Control/DMS (−), Control/DMS (+), Hippuristanol/DMS (−), and Hippuristanol/DMS (+) samples, respectively. The script also uses Samtools (version 0.1.19) [66] to remove any unmapped reads or reads that have mapped in the reverse orientation.

The sam_to_rtsc.py script was used to generate <.rtsc> files from each filtered <.sam> file generated in the previous step. Each <.rtsc> file contains the number of reverse transcriptase stops at each position of every transcript. All replicate <.rtsc> files are available as supplemental files for GSE134865. Replicate correlation was calculated using the rtsc_correlation.py script followed by the Replicate_correlation.R script.

The coverage of every transcript was calculated for every replicate from each of the DMS (+) <.rtsc> files, using the rtsc_coverage.py script. Coverage is calculated as the number of stops at every adenine or cytosine within the transcript, divided by (the length of the transcript × AC content of the transcript). For example if a transcript was 2000 nt long and had 50% AC content, it would have a coverage of 1 if there were 1000 stops at all A and C positions within the transcript. Transcripts were filtered by coverage with a threshold of 1 in every replicate. Of the 55,770 transcripts in the MCF7-specific transcriptome, 26,820 had a coverage of 1 or higher in every replicate from the control and hippuristanol DMS (+) samples. 5′ end coverage was calculated with the rtsc_end_coverage.py script, using the equation in Additional file 1: Figure S3B. All transcripts with a 5′ end coverage score less than 1.5, with *n* set to 10, were removed prior to analysis. Of the 55,770 transcripts in the MCF7-specific transcriptome, 26,393 had a 5′ coverage of 1.5 or higher in both the control and hippuristanol DMS (−) samples. One hundred twenty-five nucleotides was trimmed from the 3′ end of transcripts before any analyses. This was determined by the analysis carried out in Additional file 1: Figure S3C using the rtsc_end_coverage.py script.

The specificity and ligation bias of each sample was calculated using the rtsc_specificity.py and check_ligation_bias.py scripts respectively, and the plots were generated using the Specificity_and_ligation_bias.R script.

The rtsc_to_react.py script was used to generate <.react> files for each replicate under each condition. The script uses a DMS(−) and DMS(+) <.rtsc> file to generate a <.react> file which contains the normalized reactivity for every A and C within every transcript, as in [29]. The script either generates a <.scale> file or requires one as input. The <.scale> generated for control A was therefore used for every other replicate and condition so that the scaling was the same for every sample. All replicate <.react> files are available as supplemental files for GSE134865. The reactivity in control and hippuristanol samples was then averaged across the replicates using the react_average.py script. The <.react> files generated were split into 5′UTR, CDS, and 3′UTR regions using the same coordinates calculated to divide the <.FASTA> file (see below).

### dStruct analysis

dStruct [50] analysis was performed with the following options: reps_A = 3, reps_B = 3, min_length = 10, batches = T, check_signal_strength = T, check_nucs = T, check_quality = T in R with the dStruct. R script. Reactivities for full-length transcripts were used, and each window was assigned its location subsequently.

### RNA folding predictions

RNA sequences were folded using the batch_fold.py script which uses RNAstructure (version 6.1) [67]. Default settings were used with control and hippuristanol reactivities as restraints.

### Polysome profiling

Fifteen-centimeter plates with 70–80% confluent MCF7 cells were treated for 1 h with 150 nM hippuristanol or an equal concentration of DMSO (0.07%) by replenishment of medium. Cells were treated for 5 min with 100 μg/ml cycloheximide at 37 °C before being washed with ice-cold PBS containing 100 μg/ml cycloheximide. Cells were collected by gentle scraping and then lysed in 500 μl lysis buffer (15 mM Tris-HCl pH 7.5, 300 mM NaCl, 15 mM MgCl_2_, 2 mM DTT, 100 μg/ml cycloheximide, 1% Triton X and 1000 U/ml SuperaseIn (AM2694)) for 1 min on ice. Lysate was centrifuged at 12,000*g* for 1 min at 4 °C and the supernatant collected. Four hundred microliters was loaded onto a 10–50% sucrose density gradient (15 mM Tris-HCl pH 7.5, 300 mM NaCl, 15 mM MgCl_2_, 2 mM DTT, and 100 μg/ml cycloheximide) and centrifuged in a pre-cooled ultra-centrifuge with the SW40 Ti rotor at 38,000 rpm for 2 h at 4 °C. For total RNA samples, 50 μl lysate was added to 1 ml TRIzol and the RNA extracted as per the manufacturer’s instructions. Gradients were fractionated and 11 × 1 ml fractions were collected and the RNA precipitated overnight at − 20 °C following the addition of 3 ml guanidine HCl (7.7 M) and 4 ml 100% ethanol. Precipitated RNA was dissolved in 350 μl TE buffer and ethanol precipitated with 500 mM ammonium acetate and 1 μl GlycoBlue. RNA was then dissolved in 30 μl Tris-HCl pH 7.5, and concentrations were determined with the nanodrop. Equal volumes of RNA from fractions 1–5 and 6–11 were each pooled to form the sub-polysomal and polysomal RNA respectively. Total RNA, sub-polysomal RNA, and polysomal RNA were run on an Agilent 2100 Bioanalyzer with the Eukaryote Total RNA Nano assay, and RIN values obtained were above 9.9 for all total and polysomal RNA samples and above 8.7 for all sub-polysomal RNA samples. Three biological replicates were obtained for each sample.

Total, sub-polysomal, and polysomal samples were sent to the DNA Sequencing Facility at the Dept of Biochemistry, University of Cambridge, and underwent Illumina TrueSeq Stranded mRNA library preparation and were sequenced on a NextSeq 500 with two high output runs of 75 cycles, single-ended. 866,318,876 reads were obtained in total. The raw sequencing reads are available at the Gene Expression Omnibus (GEO) database, accession GSE134888 in fastq format.

The Bayesian model used to analyze the polysome profiling data has been described previously in [4], with minor modifications as specified below. Sequencing reads were mapped to the MCF7-specific transcriptome as per standard MMSEQ 1.0.10 instructions [68], which uses Bowtie 1.1.1. Reads that mapped to more than one location were kept, and expression levels were estimated using MMSEQ for either individual transcript splice variants or collapsed to gene units. MMDIFF [69] was used to identify mRNAs which change in total RNA expression between control and hippuristanol-treated conditions, using the standard differential expression (DE) analysis as described in [4]. To identify mRNAs for which the log-fold change in expression between control and hippuristanol-treated samples differed within the sub-polysomal and polysomal RNA, MMDIFF was used to perform a difference of difference (DOD) analysis as described in [4]. In the DOD analysis, the baseline model assumes that the log-fold change between hippuristanol and control is the same within sub-polysomal and polysomal RNA, while the alternate model allows the log-fold changes to differ. A prior probability of 0.1 that the alternate model was true was specified, and the posterior probability was thresholded liberally above 0.25 in order to declare a transcript as either eIF4A-dependent or eIF4A-antidependent. To assign mRNAs with a posterior probability above 0.25 to be eIF4A-dependent or eIF4A-antidependent, we determined the sign of the estimated log-fold change in the polysomal RNA minus the estimated log-fold change in the sub-polysomal RNA. mRNAs for which the sign was negative were declared eIF4A-dependent, and mRNAs for which the sign was positive were declared eIF4A-antidependent. mRNAs with a posterior probability less than 0.02 were declared eIF4A-independent. The output of both the DE and DOD analysis, at both the gene and transcript levels can be found in the supplemental files at GSE134888.

### MCF7-specific transcriptome

MCF-7 transcriptome sequence data were generated by Pacific Biosciences, Menlo Park, California, and additional information about the sequencing and assembly is provided at http://datasets.pacb.com.s3.amazonaws.com/2015/IsoSeqHumanMCF7Transcriptome/list.html. IsoSeq_MCF7_2015edition_polished.unimapped.fasta was downloaded, which contains 55,770 transcripts. In order to split each transcript into 5′UTR, CDS, and 3′UTR sequences, the manually curated coding sequences from RefSeq release 85 (NM transcripts only) were blasted against the whole MCF7-specific transcriptome. Blast hits that started at position 1 of the RefSeq CDS were used to identify the translation start site within the MCF7 transcript. Blast hits that extended to the end of the RefSeq CDS were used to identify translation stop sites. Only transcripts whose translation start and stop sites were identified by this method, and that resulted in a CDS which was equally divisible by 3, were included in the final annotation. This resulted in 13,132 fully annotated transcripts. The splicing_MCF7_2015_FASTA.py script was used to generate three separate <.FASTA> files, one for each region.

### G-quadruplex predictions with G4RNA screener

We used G4 RNA screener [45] to predict the likelihood of folded G-quadruplexes within the 5′UTRs of eIF4A-dependent and eIF4A-independent mRNAs. We ran the script with default settings, with a window size of 50 nt and step size of 10 nt to generate G4NN scores. We then selected the highest G4NN score per 5′UTR.

### Reporter-based assays

Reporter RNA was designed to have an unstructured 5′UTR with the following sequence GGGCAAGAA (CAA)_24_CACC. The sequence, including the T7 RNA polymerase binding site, was cloned using annealed oligos, into the pGL3-promoter plasmid (Promega E1761), between the HindIII and NcoI restriction sites, directly upstream of the Fluc open reading frame. An (A)_49_ sequence, followed by a NsiI site, was cloned downstream of the ORF so that following linearization with Nsi1 and blunt ending with Klenow fragment (NEB M0210S), RNA containing an (A)_49_ tail could be transcribed directly from the template.

RNA was transcribed with the TranscriptAid T7 High Yield Transcription Kit (ThermoFisher K0441) as per the manufacturer’s instructions using 7.5 mM ATP/CTP/UTP, 1.5 mM GTP, and 6 mM ARCA (NEB S1411S), followed by acid-phenol chloroform extraction and ethanol precipitation with ammonium acetate.

For sequencing gels, 75 μl nuclease untreated Rabbit Reticulocyte Lysate (Promega L4151), supplemented with 25 μM haemin, 25 μg/ml creatine kinase, 3 mg/ml creatine phosphate, 50 μg/ml liver tRNAs, and 3 mM glucose was added to 3 μl amino acid mix (1 mM), 6 μl KCl (2.5 M), 3 μl MgOAc (25 mM), 1 μl RNaseIn plus Ribonuclease Inhibitor (40 U/μl) (Promega), and 6 μg RNA and up to 150 μl water. The reaction was incubated at 30 °C for 15 min for harringtonine assays and 30 min for hippuristanol assays before addition of DMS to 50 mM and further incubation for 5 min. DMS was quenched with 250 mM DTT, and RNA was extracted with TRIzol LS as per the manufacturer’s instructions. RT reactions were carried out with a ^32^P labeled primer specific for the open reading frame of Fluc: 5′TTCCAGCGGATAGAATGGCG3′. Extracted RNA was mixed with 1 pmol primer and diluted to 6.5 μl. One-microliter 10X buffer (200 mM Tris HCl (pH 8.4), 500 mM KCl) was added, and samples were heated at 95 °C in a heat block for 1 min and then immediately placed into a different heat block at 55 °C for 1 min. Two-microliter 5X buffer (2.5 mM dNTPs, 20 mM MgCl2, 5 mM DTT) was added along with 0.5 μl SuperScript III (200 U/μl), and the sample was incubated for 20 min at 55 °C. One-microliter NaOH was added, and RNA was hydrolyzed and enzyme denatured at 95 °C for 10 min. Eleven-microliter 2X loading buffer (95% formamide, 20 mM Tris HCl (pH 7.5), 20 mM EDTA, 0.025% bromophenol) was added, and the sample was incubated for 3 min at 95 °C to denature the cDNA. Five-microliter samples were loaded onto a pre-run 6% polyacrylamide, 8.3 M urea sequencing gel, and run at 45 W for 1 h. The gel was fixed in 10% methanol and 10% acetic acid and dried for 2 h at 80 °C before overnight exposure to a GE Storage Phosphor Screen followed by visualization on a Typhoon FLA 7000.

## Supplementary information

**Additional file 1: Figure S1.** Structure-seq2 and polysome profiling quality control. **Figure S2.** Replicate correlation and coverage thresholds. **Figure S3.** Assessing 5′ and 3′ end coverage. **Figure S4.** Average reactivity, predicted fold metrics and Gini coefficients. **Figure S5.** mRNAs gain in structure following hippuristanol treatment most within the CDS and the 3′ end of the 5’UTR. **Figure S6.** Highly translated mRNAs are less structured in the 3′ most 20 nt of 5’UTRs. **Figure S7.** eIF4A-dependent mRNAs gain in structure upon hippuristanol treatment immediately upstream of the CDS. **Figure S8.** dStruct FDR adjusted *p*-values and window sizes. **Table S1.** Summary of sequencing reads obtained for Structure-seq2. **Table S2.** Summary of scripts used to create each figure.

**Additional file 2.** Review history.

## Data Availability

The datasets generated and analyzed during the current study are available in the Gene Expression Omnibus (GEO) database accessions GSE134865 [70] and GSE134888 [71] which can be found at https://www.ncbi.nlm.nih.gov/geo/query/acc.cgi?acc=GSE134865 and https://www.ncbi.nlm.nih.gov/geo/query/acc.cgi?acc=GSE134888. All scripts and input data required to replicate the figures are available from GitHub using the following link https://github.com/Bushell-lab/Structure-seq2-with-hippuristanol-treatment-in-MCF7-cells [72]. All StructureFold2 scripts are available from GitHub using the following link https://github.com/StructureFold2/StructureFold2. Additional file 1: Table S2 shows which scripts were used to output which figure panels.

## References

[1] Vaklavas C, Blume SW, Grizzle WE (2017). Translational dysregulation in cancer: molecular insights and potential clinical applications in biomarker development. Front Oncol.

[2] Bhat M, Robichaud N, Hulea L, Sonenberg N, Pelletier J, Topisirovic I (2015). Targeting the translation machinery in cancer. Nat Rev Drug Discov.

[3] Chu Jennifer, Cargnello Marie, Topisirovic Ivan, Pelletier Jerry (2016). Translation Initiation Factors: Reprogramming Protein Synthesis in Cancer. Trends in Cell Biology.

[4] Modelska A, Turro E, Russell R, Beaton J, Sbarrato T, Spriggs K (2015). The malignant phenotype in breast cancer is driven by eIF4A1-mediated changes in the translational landscape. Cell Death Dis.

[5] Heerma van Voss MR, van Diest PJ, Raman V (2017). Targeting RNA helicases in cancer: the translation trap. Biochim Biophys Acta.

[6] Malka-Mahieu H, Newman M, Desaubry L, Robert C, Vagner S (2017). Molecular pathways: the eIF4F translation initiation complex-new opportunities for cancer treatment. Clin Cancer Res.

[7] Pelletier J, Graff J, Ruggero D, Sonenberg N (2015). Targeting the eIF4F translation initiation complex: a critical nexus for cancer development. Cancer Res.

[8] Boussemart L, Malka-Mahieu H, Girault I, Allard D, Hemmingsson O, Tomasic G (2014). eIF4F is a nexus of resistance to anti-BRAF and anti-MEK cancer therapies. Nature..

[9] Malka-Mahieu Hélène, Girault Isabelle, Rubington Margot, Leriche Melissa, Welsch Caroline, Kamsu-Kom Nyam, Zhao Qian, Desaubry Laurent, Vagner Stéphan, Robert Caroline (2016). Synergistic effects of eIF4A and MEK inhibitors on proliferation of NRAS-mutant melanoma cell lines. Cell Cycle.

[10] Robert F, Roman W, Bramoulle A, Fellmann C, Roulston A, Shustik C (2014). Translation initiation factor eIF4F modifies the dexamethasone response in multiple myeloma. Proc Natl Acad Sci U S A.

[11] Wolfe AL, Singh K, Zhong Y, Drewe P, Rajasekhar VK, Sanghvi VR (2014). RNA G-quadruplexes cause eIF4A-dependent oncogene translation in cancer. Nature..

[12] Wiegering A, Uthe FW, Jamieson T, Ruoss Y, Huttenrauch M, Kuspert M (2015). Targeting translation initiation bypasses signaling crosstalk mechanisms that maintain high MYC levels in colorectal cancer. Cancer Discov.

[13] Jackson RJ, Hellen CU, Pestova TV (2010). The mechanism of eukaryotic translation initiation and principles of its regulation. Nat Rev Mol Cell Biol.

[14] Hinnebusch AG (2014). The scanning mechanism of eukaryotic translation initiation. Annu Rev Biochem.

[15] Andreou AZ, Klostermeier D (2013). The DEAD-box helicase eIF4A: paradigm or the odd one out?. RNA Biol.

[16] Sokabe M, Fraser CS (2017). A helicase-independent activity of eIF4A in promoting mRNA recruitment to the human ribosome. Proc Natl Acad Sci U S A.

[17] Yourik P, Aitken CE, Zhou F, Gupta N, Hinnebusch AG, Lorsch JR. Yeast eIF4A enhances recruitment of mRNAs regardless of their structural complexity. eLife. 2017;6:e31476. 10.7554/eLife.31476 PMC5726853 29192585

[18] Duncan R, Hershey JW (1983). Identification and quantitation of levels of protein synthesis initiation factors in crude HeLa cell lysates by two-dimensional polyacrylamide gel electrophoresis. J Biol Chem.

[19] Rubio CA, Weisburd B, Holderfield M, Arias C, Fang E, DeRisi JL (2014). Transcriptome-wide characterization of the eIF4A signature highlights plasticity in translation regulation. Genome Biol.

[20] Rouskin S, Zubradt M, Washietl S, Kellis M, Weissman JS (2014). Genome-wide probing of RNA structure reveals active unfolding of mRNA structures in vivo. Nature..

[21] Biffi G, Di Antonio M, Tannahill D, Balasubramanian S (2014). Visualization and selective chemical targeting of RNA G-quadruplex structures in the cytoplasm of human cells. Nat Chem.

[22] Laguerre A, Hukezalie K, Winckler P, Katranji F, Chanteloup G, Pirrotta M (2015). Visualization of RNA-Quadruplexes in live cells. J Am Chem Soc.

[23] Guo J. U., Bartel D. P. (2016). RNA G-quadruplexes are globally unfolded in eukaryotic cells and depleted in bacteria. Science.

[24] Waldron JA, Raza F, Le Quesne J (2018). eIF4A alleviates the translational repression mediated by classical secondary structures more than by G-quadruplexes. Nucleic Acids Res.

[25] Weldon C, Eperon IC, Dominguez C (2016). Do we know whether potential G-quadruplexes actually form in long functional RNA molecules?. Biochem Soc Trans.

[26] Lee YJ, Wang Q, Rio DC (2018). Coordinate regulation of alternative pre-mRNA splicing events by the human RNA chaperone proteins hnRNPA1 and DDX5. Genes Dev.

[27] Lai YH, Choudhary K, Cloutier SC, Xing Z, Aviran S, Tran EJ (2019). Genome-wide discovery of DEAD-box RNA helicase targets reveals RNA structural remodeling in transcription termination. Genetics..

[28] Guenther UP, Weinberg DE, Zubradt MM, Tedeschi FA, Stawicki BN, Zagore LL (2018). The helicase Ded1p controls use of near-cognate translation initiation codons in 5′ UTRs. Nature..

[29] Ritchey LE, Su Z, Tang Y, Tack DC, Assmann SM, Bevilacqua PC (2017). Structure-seq2: sensitive and accurate genome-wide profiling of RNA structure in vivo. Nucleic Acids Res.

[30] Iwasaki Shintaro, Iwasaki Wakana, Takahashi Mari, Sakamoto Ayako, Watanabe Chiduru, Shichino Yuichi, Floor Stephen N., Fujiwara Koichi, Mito Mari, Dodo Kosuke, Sodeoka Mikiko, Imataka Hiroaki, Honma Teruki, Fukuzawa Kaori, Ito Takuhiro, Ingolia Nicholas T. (2019). The Translation Inhibitor Rocaglamide Targets a Bimolecular Cavity between eIF4A and Polypurine RNA. Molecular Cell.

[31] Wells SE, Hughes JM, Igel AH, Ares M (2000). Use of dimethyl sulfate to probe RNA structure *in vivo*. Methods Enzymol.

[32] Cencic R, Pelletier J (2016). Hippuristanol - a potent steroid inhibitor of eukaryotic initiation factor 4A. Translation (Austin).

[33] Iwasaki S, Floor SN, Ingolia NT (2016). Rocaglates convert DEAD-box protein eIF4A into a sequence-selective translational repressor. Nature..

[34] Tack David C., Tang Yin, Ritchey Laura E., Assmann Sarah M., Bevilacqua Philip C. (2018). StructureFold2: Bringing chemical probing data into the computational fold of RNA structural analysis. Methods.

[35] Gandin Valentina, Masvidal Laia, Hulea Laura, Gravel Simon-Pierre, Cargnello Marie, McLaughlan Shannon, Cai Yutian, Balanathan Preetika, Morita Masahiro, Rajakumar Arjuna, Furic Luc, Pollak Michael, Porco John A., St-Pierre Julie, Pelletier Jerry, Larsson Ola, Topisirovic Ivan (2016). nanoCAGE reveals 5′ UTR features that define specific modes of translation of functionally related MTOR-sensitive mRNAs. Genome Research.

[36] Beaudoin JD, Novoa EM, Vejnar CE, Yartseva V, Takacs CM, Kellis M (2018). Analyses of mRNA structure dynamics identify embryonic gene regulatory programs. Nat Struct Mol Biol.

[37] Mizrahi Orel, Nachshon Aharon, Shitrit Alina, Gelbart Idit A., Dobesova Martina, Brenner Shirly, Kahana Chaim, Stern-Ginossar Noam (2018). Virus-Induced Changes in mRNA Secondary Structure Uncover cis-Regulatory Elements that Directly Control Gene Expression. Molecular Cell.

[38] Wittebolle L, Marzorati M, Clement L, Balloi A, Daffonchio D, Heylen K (2009). Initial community evenness favours functionality under selective stress. Nature..

[39] Zubradt M, Gupta P, Persad S, Lambowitz AM, Weissman JS, Rouskin S (2017). DMS-MaPseq for genome-wide or targeted RNA structure probing in vivo. Nat Methods.

[40] Ding Y, Tang Y, Kwok CK, Zhang Y, Bevilacqua PC, Assmann SM (2014). *In vivo* genome-wide profiling of RNA secondary structure reveals novel regulatory features. Nature..

[41] Soto Rifo R, Ricci EP, Decimo D, Moncorge O, Ohlmann T (2007). Back to basics: the untreated rabbit reticulocyte lysate as a competitive system to recapitulate cap/poly(A) synergy and the selective advantage of IRES-driven translation. Nucleic Acids Res.

[42] Fresno M, Jimenez A, Vazquez D (1977). Inhibition of translation in eukaryotic systems by harringtonine. Eur J Biochem.

[43] Lee S., Liu B., Lee S., Huang S.-X., Shen B., Qian S.-B. (2012). Global mapping of translation initiation sites in mammalian cells at single-nucleotide resolution. Proceedings of the National Academy of Sciences.

[44] Masvidal L, Hulea L, Furic L, Topisirovic I, Larsson O (2017). mTOR-sensitive translation: cleared fog reveals more trees. RNA Biol.

[45] Garant Jean-Michel, Perreault Jean-Pierre, Scott Michelle S (2017). Motif independent identification of potential RNA G-quadruplexes by G4RNA screener. Bioinformatics.

[46] Kwok Chun Kit, Sahakyan Aleksandr B., Balasubramanian Shankar (2016). Structural Analysis using SHALiPE to Reveal RNA G-Quadruplex Formation in Human Precursor MicroRNA. Angewandte Chemie International Edition.

[47] Rogers GW, Richter NJ, Merrick WC (1999). Biochemical and kinetic characterization of the RNA helicase activity of eukaryotic initiation factor 4A. J Biol Chem.

[48] Garcia-Garcia C, Frieda KL, Feoktistova K, Fraser CS, Block SM (2015). Factor-dependent processivity in human eIF4A DEAD-box helicase. Science.

[49] Bailey TL, Elkan C (1994). Fitting a mixture model by expectation maximization to discover motifs in biopolymers. Proc Int Conf Int Syst Mol Biol.

[50] Choudhary K, Lai YH, Tran EJ, Aviran S (2019). dStruct: identifying differentially reactive regions from RNA structurome profiling data. Genome Biol.

[51] Gallie DR, Ling J, Niepel M, Morley SJ, Pain VM (2000). The role of 5′-leader length, secondary structure and PABP concentration on cap and poly(A) tail function during translation in Xenopus oocytes. Nucleic Acids Res.

[52] Svitkin YV, Pause A, Haghighat A, Pyronnet S, Witherell G, Belsham GJ (2001). The requirement for eukaryotic initiation factor 4A (elF4A) in translation is in direct proportion to the degree of mRNA 5′ secondary structure. RNA (New York).

[53] Pestova TV, Kolupaeva VG (2002). The roles of individual eukaryotic translation initiation factors in ribosomal scanning and initiation codon selection. Genes Dev.

[54] Babendure JR, Babendure JL, Ding JH, Tsien RY (2006). Control of mammalian translation by mRNA structure near caps. RNA (New York).

[55] Sen ND, Zhou F, Ingolia NT, Hinnebusch AG (2015). Genome-wide analysis of translational efficiency reveals distinct but overlapping functions of yeast DEAD-box RNA helicases Ded1 and eIF4A. Genome Res.

[56] Sen ND, Gupta N, KA S, Preiss T, Lorsch JR, Hinnebusch AG. Functional interplay between DEAD-box RNA helicases Ded1 and Dbp1 in preinitiation complex attachment and scanning on structured mRNAs in vivo. Nucleic Acids Res. 2019;47(16):8785-8806. 10.1093/nar/gkz595 PMC7145680 31299079

[57] Gao Z, Putnam AA, Bowers HA, Guenther UP, Ye X, Kindsfather A, et al. Coupling between the DEAD-box RNA helicases Ded1p and eIF4A. eLife. 2016;5:e16408. 10.7554/eLife.16408 PMC4990422 27494274

[58] Gupta N, Lorsch JR, Hinnebusch AG. Yeast Ded1 promotes 48S translation pre-initiation complex assembly in an mRNA-specific and eIF4F-dependent manner. eLife. 2018;7:e38892. 10.7554/eLife.38892 PMC6181565 30281017

[59] Soto-Rifo R, Ohlmann T (2013). The role of the DEAD-box RNA helicase DDX3 in mRNA metabolism. Wiley Interdiscip Rev RNA.

[60] Soto-Rifo R, Rubilar PS, Limousin T, de Breyne S, Decimo D, Ohlmann T (2012). DEAD-box protein DDX3 associates with eIF4F to promote translation of selected mRNAs. EMBO J.

[61] Pisareva VP, Pisarev AV, Komar AA, Hellen CU, Pestova TV (2008). Translation initiation on mammalian mRNAs with structured 5'UTRs requires DExH-box protein DHX29. Cell..

[62] Parsyan A, Shahbazian D, Martineau Y, Petroulakis E, Alain T, Larsson O (2009). The helicase protein DHX29 promotes translation initiation, cell proliferation, and tumorigenesis. Proc Natl Acad Sci U S A.

[63] Ding Y, Kwok CK, Tang Y, Bevilacqua PC, Assmann SM (2015). Genome-wide profiling of in vivo RNA structure at single-nucleotide resolution using structure-seq. Nat Protoc.

[64] Martin Marcel (2011). Cutadapt removes adapter sequences from high-throughput sequencing reads. EMBnet.journal.

[65] Langmead B, Salzberg SL (2012). Fast gapped-read alignment with Bowtie 2. Nat Methods.

[66] Li H, Handsaker B, Wysoker A, Fennell T, Ruan J, Homer N (2009). The sequence alignment/map format and SAMtools. Bioinformatics.

[67] Reuter JS, Mathews DH (2010). RNAstructure: software for RNA secondary structure prediction and analysis. BMC bioinformatics.

[68] Turro E, Su SY, Goncalves A, Coin LJ, Richardson S, Lewin A (2011). Haplotype and isoform specific expression estimation using multi-mapping RNA-seq reads. Genome Biol.

[69] Turro E, Astle WJ, Tavare S (2014). Flexible analysis of RNA-seq data using mixed effects models. Bioinformatics.

[70] Waldron JA, Tack DC, Ritchey LE, Gillen SL, Wilczynska A, Turro E, Bevilacqua PC, Assmann SM, Bushell M, Le Quesne J. Transcriptome-wide analysis of RNA structure in MCF7 cells with and without inhibition of eIF4A by hippuristanol treatment. Gene Exp Omnibus. 2019; https://www.ncbi.nlm.nih.gov/geo/query/acc.cgi?acc=GSE134865.

[71] Waldron JA, Tack DC, Ritchey LE, Gillen SL, Wilczynska A, Turro E, Bevilacqua PC, Assmann SM, Bushell M, Le Quesne J. Transcriptome wide analysis of translation efficiency in MCF7 cells using polysome profiling with and without eIF4A inhibition by hippuristanol treatment. Gene Exp Omnibus. 2019; https://www.ncbi.nlm.nih.gov/geo/query/acc.cgi?acc=GSE134888.

[72] Waldron JA, Tack DC, Ritchey LE, Gillen SL, Wilczynska A, Turro E, Bevilacqua PC, Assmann SM, Bushell M, Le Quesne J. Transcriptome-wide analysis of RNA structure in MCF7 cells with and without inhibition of eIF4A by hippuristanol treatment. Scripts and data. Github repository. 2019; https://github.com/Bushell-lab/Structure-seq2-with-hippuristanol-treatment-in-MCF7-cells.

