# Supplementary material for: mRNA structural elements immediately upstream of the start codon dictate dependence upon eIF4A helicase activity
Source: Genome Biol. 2019 Dec 30;20:300. doi: 10.1186/s13059-019-1901-2 (PMC6936103; doi:10.1186/s13059-019-1901-2)
Supplement: Supplementary file 1 — Additional file 1: Figure S1. Structure-seq2 and polysome profiling quality control. Figure S2. Replicate correlation and coverage thresholds. Figure S3. Assessing 5′ and 3′ end coverage. Figure S4. Average reactivity, predicted fold metrics and Gini coefficients. Figure S5. mRNAs gain in structure following hippuristanol treatment most within the CDS and the 3′ end of the 5’UTR. Figure S6. Highly translated mRNAs are less structured in the 3′ most 20 nt of 5’UTRs. Figure S7. eIF4A-dependent mRNAs gain in structure upon hippuristanol treatment immediately upstream of the CDS. Figure S8. dStruct FDR adjusted p-values and window sizes. Table S1. Summary of sequencing reads obtained for Structure-seq2. Table S2. Summary of scripts used to create each figure. [file 13059_2019_1901_MOESM1_ESM.pdf]

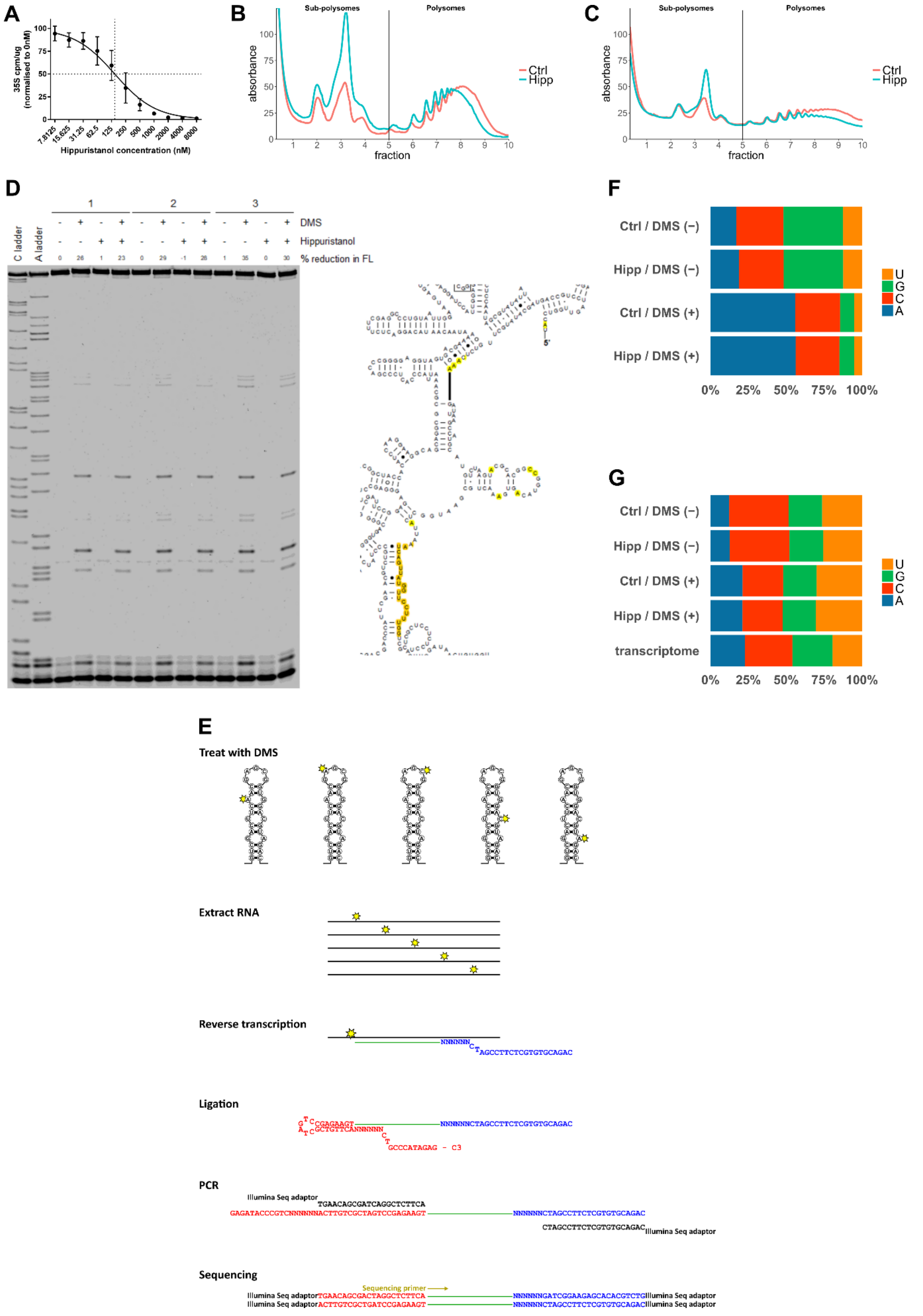

Figure S1. Structure-seq2 and polysome profiling quality control. (A) Rates of protein synthesis across a range of hippuristanol concentrations in MCF7 cells as measured by ^{35}S protein labelling. n=3 biological replicates and error bars represent SEM. **(B-C)** Polysome traces from the second and third biological replicate with and without hippuristanol. **(D)** Sequencing gel showing that the conditions of DMS treatment were under single-hit

kinetics. The gel shows the cDNA from reverse transcription reactions of the RNA from all three replicates, with and without hippuristanol, with and without DMS, with a primer which binds roughly 100nt from the 5' end of the 18S rRNA (highlighted in orange on 18S rRNA structure). Percentage decrease in full length (FL) band was calculated and shown to be between 20-30% for all DMS treated samples, indicating single-hit kinetics. The aborted products indicate stalling at single stranded adenosines and cytosines (highlighted in yellow on 18S rRNA structure) as expected. **(E)** Diagrammatic representation of the Structure-seq2 library preparation steps undertaken. Firstly, cells are treated with DMS under single-hit kinetics and the RNA is extracted. Using random hexamers with a tail of known sequence, reverse transcription is carried out on poly(A) selected RNA. A 3' hairpin adaptor is ligated onto the 3' ends of the cDNA library using T4 DNA ligase. PCR is then used to incorporate the Illumina sequencing adaptors and the libraries are sequenced using a custom primer so that the first nucleotide that is sequenced is the nucleotide directly 5' of the DMS modified nucleotide. **(F)** Bar chart representing the percentage of each nucleotide responsible for each stalling event in each sample. This is over 85% in the DMS (+) samples, indicating good quality libraries. **(G)** To assess for any ligation bias, the complement of the first nucleotide sequenced is determined for every read, as this is the nucleotide which was ligated to the 3' hairpin adaptor. The percentage of each nucleotide for each sample is plotted along with the weighted transcriptome, which is the percentage of each nucleotide within all sequencing reads. There is little difference between the DMS (+) samples and the transcriptome, indicating minimal ligation bias. The deviation in the DMS (-) samples can likely be explained by the fact that these are hard stops, so are occurring in highly structured regions or at modified nucleotides.

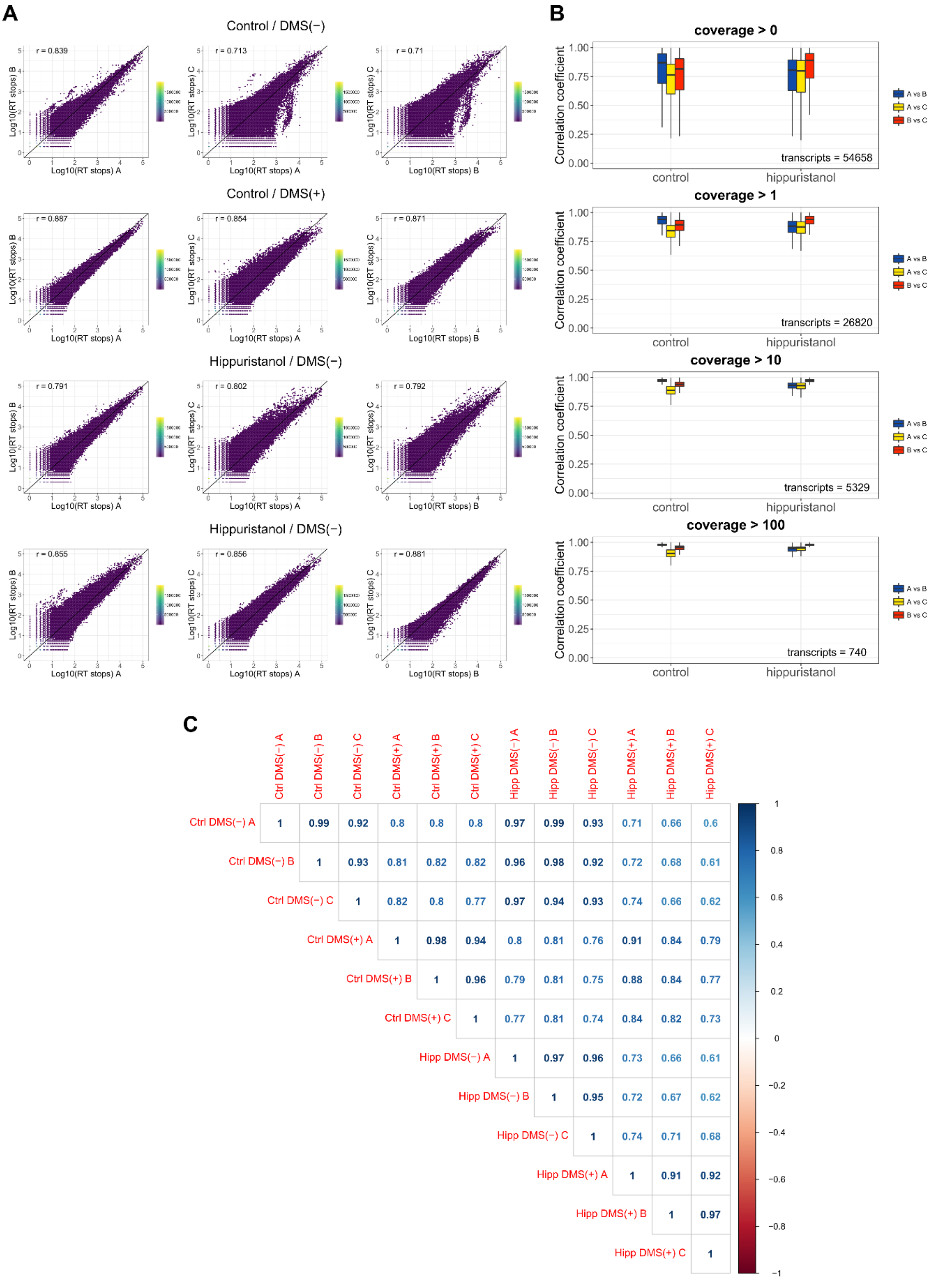

Figure S2. Replicate correlation and coverage thresholds. (A) Replicate correlation was calculated between all three pairs of replicates, across the whole transcriptome. Correlation coefficients were calculated with a Pearson test. **(B)** Boxplots depict the replicate correlation between DMS (+) samples for each transcript with a coverage above increasing thresholds. **(C)** Correlation matrix showing the transcriptome replicate correlation between all replicates and all samples, at a coverage threshold of 1. This is the coverage threshold used for the analysis throughout the rest of this paper.

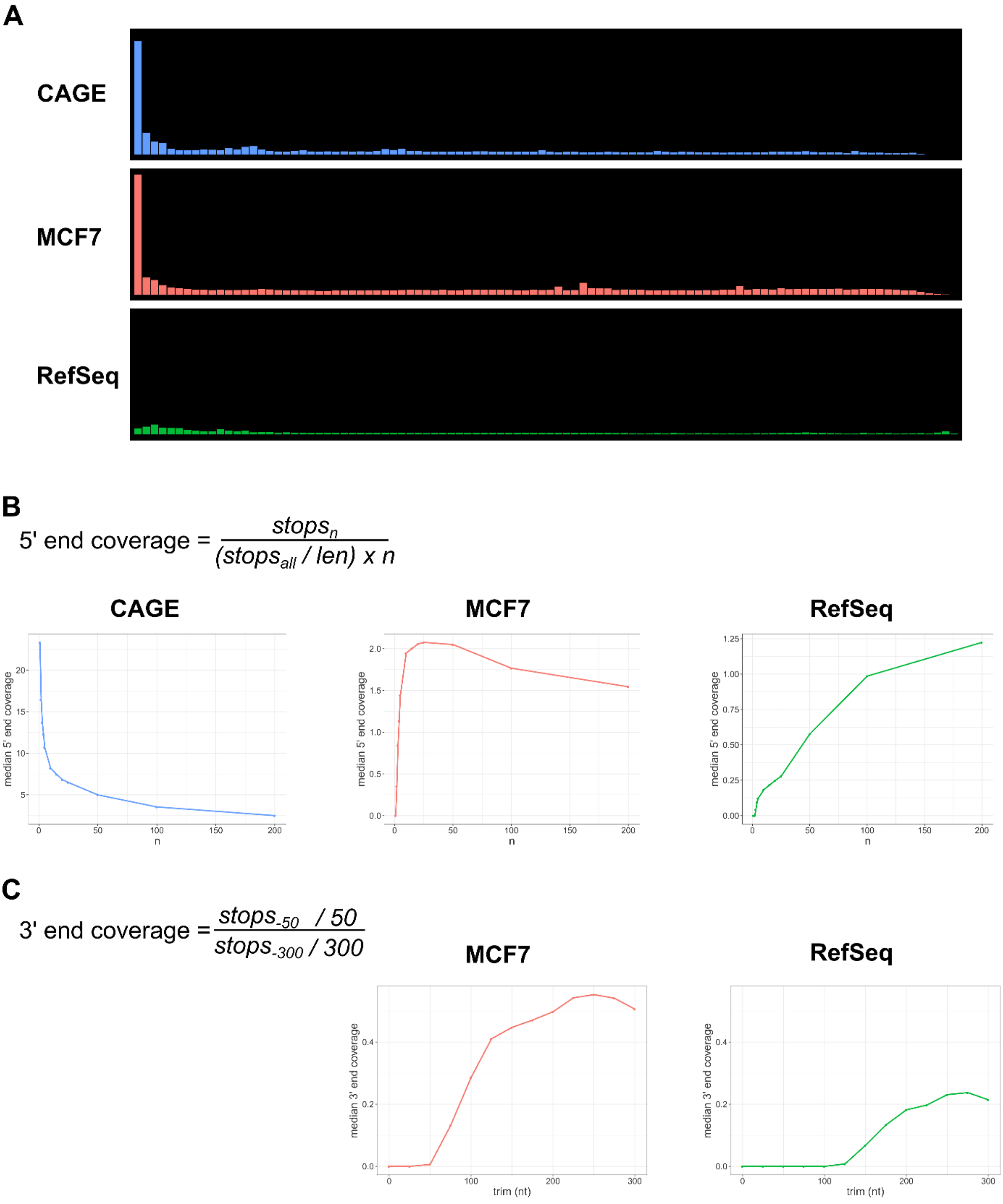

Figure S3. Assessing 5' and 3' end coverage. (A) The accuracy of 5' end annotation in three different transcriptomes was assessed by plotting the distribution of stops in the DMS (-) libraries after mapping to the respective transcriptome. As these libraries were randomly primed, the distribution of stops across the transcript should be even, except there should be a peak of reads at the 5' end of the transcript, as this should be the only positionally biased hard stop among all transcripts. We compared manually curated RefSeq transcripts with a transcriptome based on the nanoCAGE data from Gandin *et al.* (2016) (35) and also to a MCF7 specific transcriptome that was created by Pacific Biosciences (see the "Methods" section), based on long range sequencing reads. A peak of stops at the 5' end is only observed in the two transcriptomes based on sequencing data from MCF7 cells, demonstrating that these transcriptomes have significantly better 5' end annotation, compared to the RefSeq transcriptome. **(B)** 5' end coverage scores were calculated to filter transcripts based on how likely the annotated 5' end reflects its true 5' end. This is calculated using the formula shown, which measures the enrichment of hard stops at the 5' end of each transcript in the DMS (-) libraries. The number of stops in the first *n* number of nucleotides is divided by the average number of stops for an equal sized region of the transcript,

where *len* is the length of the transcript and *n* is the number of nucleotides. 5' end coverage was calculated for all transcripts within each transcriptome, from the DMS (-) libraries, while varying the size of n. If the true 5' end is annotated correctly, increasing the size of n should decrease the 5' end coverage score. Plotting the median 5' end coverage scores for all transcripts against *n*, demonstrates that the nanaCAGE data is most precise, while the RefSeq annotation is very inaccurate. While the MCF7 specific transcriptome is less accurate than the nanoCAGE transcriptome, it is still significantly more accurate than the RefSeq annotation and has sequence information for the whole transcript, which is lacking in the nanoCAGE data. Also, there were only 5,945 transcripts within the nanoCAGE based transcriptome but 55,770 transcripts within the MCF7 specific transcriptome. For these reasons we decided that the MCF7 specific transcriptome would be preferable, but that we should filter transcripts based on their 5' end coverage. These data also further support the quality of our Structure-seq2 libraries, in being in exact agreement with the nanoCAGE data on the 5' ends of transcripts. **(C)** As the Structure-seq2 libraries are randomly rather than oligo(dT) primed, coverage at the extreme 3' end will be poor. To empirically determine how many nucleotides to trim from the 3' end, we calculated 3' end coverage, by dividing the average number of stops in the 3' most 50nt by the average number of stops in the 3' most 300nt, in the DMS (-) libraries. The median 3' end coverage score is plotted for the MCF7 specific and the RefSeq transcriptomes, after trimming differing numbers of nucleotides from the 3' end. This analysis shows that 125nt should be trimmed from the 3' end prior to any analysis. It also further supports the superiority of the MCF7 specific transcriptome compared to the RefSeq annotation. The 3' end coverage analysis was not carried out on the nanoCAGE data as this data only has information for the 5' end.

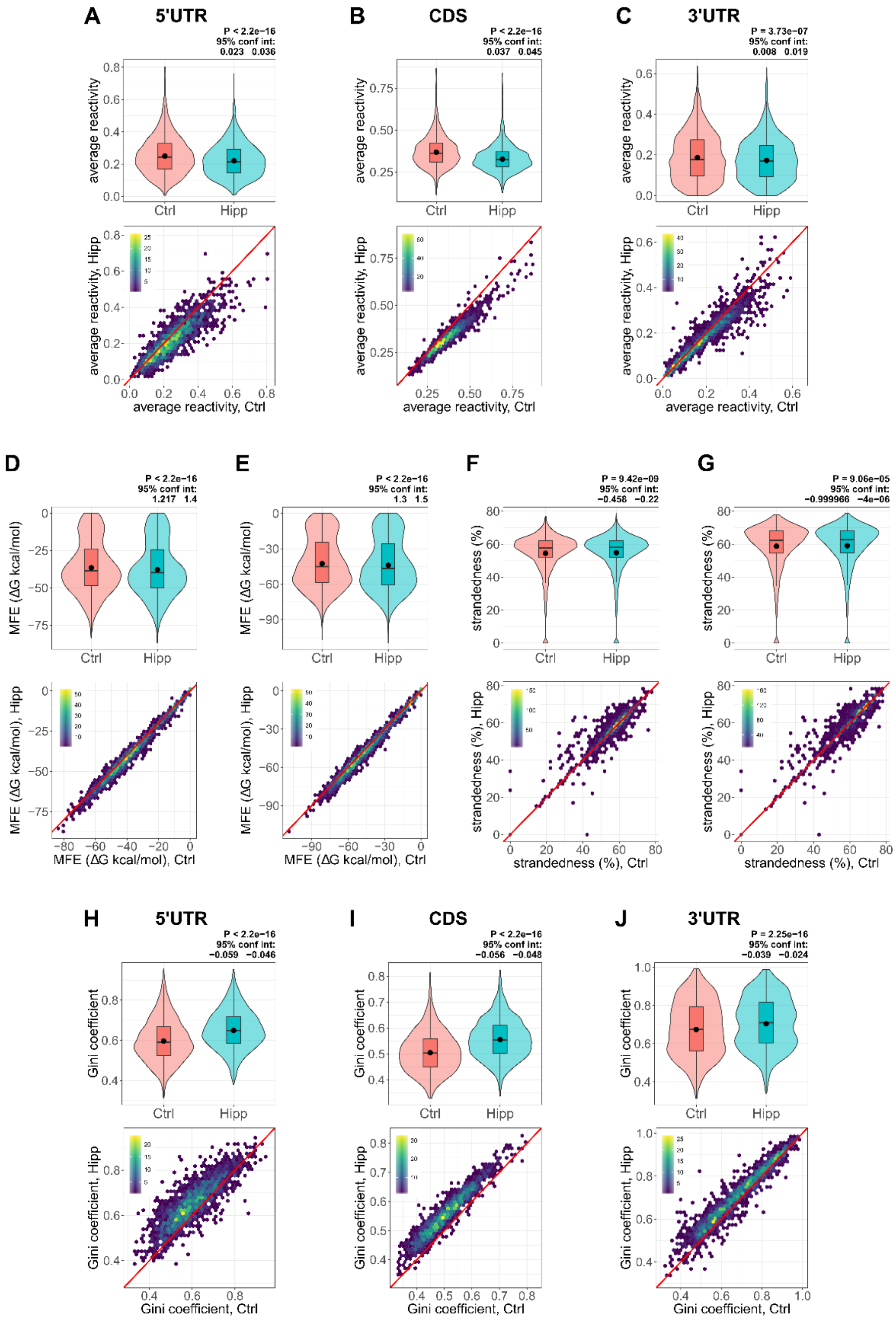

Figure S4. Average reactivity, predicted fold metrics and Gini coefficients. (A-C) Violin and density scatter plots depicting the average DMS reactivity with and without hippuristanol, within the 5'UTRs, CDSs and 3'UTRs. **(D-G)** Violin and density scatter plots showing **(D)** the average minimum free energy (MFE), **(E)** the minimum MFE, **(F)** the average percentage of base-paired nucleotides (strandedness) and **(G)** the maximum strandedness, per 5'UTR of the predicted folds from all 100nt windows, using control or hippuristanol reactivities as restraints. Full length 5'UTRs were folded for all 5'UTRs less than 100nt in length. **(H-J)** Gini coefficients of the DMS reactivity within the 5'UTR, CDS or 3'UTR. Violin plots include boxplots, with the mean denoted by a dot. Scatter plots are colour coded by density, with the number of transcripts per hexagon denoted in the legend. P values and 95% confidence intervals were calculated using a paired, two-sided Wilcoxon test. Plots show all 1,883 mRNAs after filtering by coverage and 5' end coverage and selecting the most abundant transcript per gene.

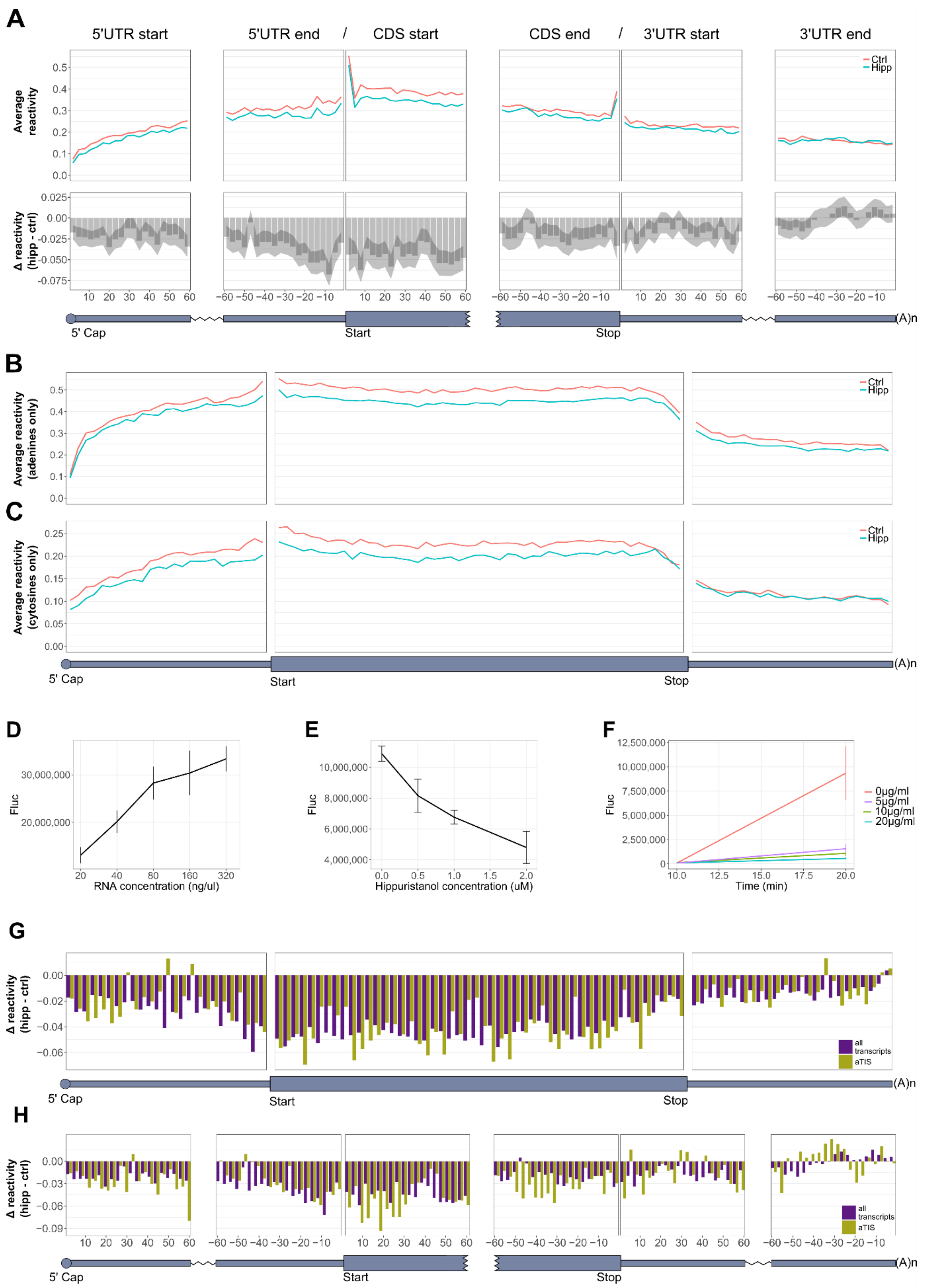

Figure S5. mRNAs gain in structure following hippuristanol treatment most within the CDS and the 3' end of the 5'UTR. (A) Average reactivities (top panel) and average Δ reactivities (hipp – control) (bottom panel) for the first and last 60nt of UTRs and CDS for all transcripts included in Figure 1C. Each data point is the average of 3nts. Shaded area represents 95% confidence limits for the difference in means between control and hippuristanol mRNAs within each 3nt, calculated by a paired two-sided t-test. **(B-C)** Average reactivities of specifically (B)

adenines and (C) cytosines, binned across the length of all transcripts included in Figure 1C. **(D)** Fluc activity in nuclease untreated rabbit reticulocyte lysate after 30min with the structure-less $(CAA)_{24}$ 5'UTR reporter (see the "Methods" section) with varying concentrations of RNA. Data show that 40ng/µl is within the linear range, demonstrating that the majority of reporter RNA used in our DMS reactivity experiments in Figures 1D-E is being translated in the lysate. n=3 and error bars represent SEM. **(E)** Fluc activity in nuclease untreated rabbit reticulocyte lysate after 30min with the structure-less $(CAA)_{24}$ 5'UTR reporter with varying concentrations of hippuristanol. n=3 and error bars represent SEM. **(F)** Fluc activity in nuclease untreated rabbit reticulocyte lysate after 10 and 20min with the structure-less $(CAA)_{24}$ 5'UTR reporter with varying concentrations of Harringtonine. n=3 and error bars represent SEM. **(G)** Binned Δ reactivity across the length of the UTRs (25 bins) and coding sequence (50 bins) for mRNAs that were found to only initiate translation from the annotated start site (aTIS) (182 mRNAs), based on data from Lee *et al.* (2012) (43), compared to all other mRNAs from Figure 1C and panel A. **(H)** Average Δ reactivity for the first and last 60nts of the UTRs and CDS for the same groups of mRNAs as in panel G. Each bar represents 3nt.

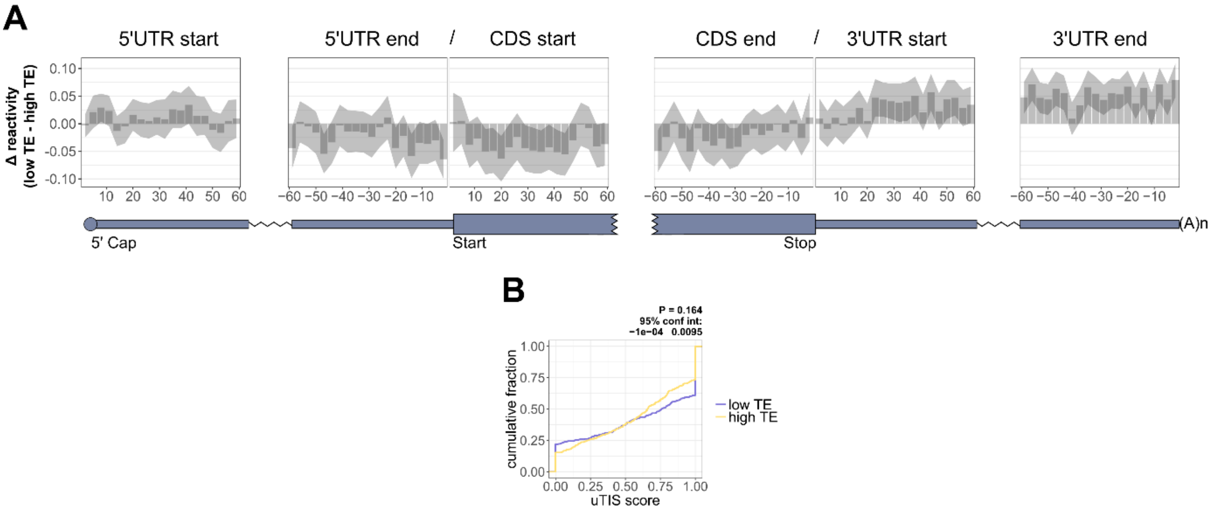

Figure S6. Highly translated mRNAs are less structured in the 3' most 20nt of 5'UTRs. (A) Average Δ reactivity between the low TE and high TE mRNAs under control conditions for the first and last 60nt of UTRs and CDS for all transcripts included in Figure 2E. Shaded area represents 95% confidence limits for the difference in means between the two groups of mRNAs within each 3nt, calculated by an un-paired two-sided t-test. **(B)** Cumulative distribution function plots of upstream translation initiation sites (uTIS) scores for high TE and low TE mRNAs. uTIS scores were calculated by dividing the number of reads mapped to upstream start sites by the number of reads mapped to both upstream and the annotated start sites based on data from Lee *et al.* (2012) (43). P values and 95% confidence intervals were calculated using an un-paired, two-sided Wilcoxon test.

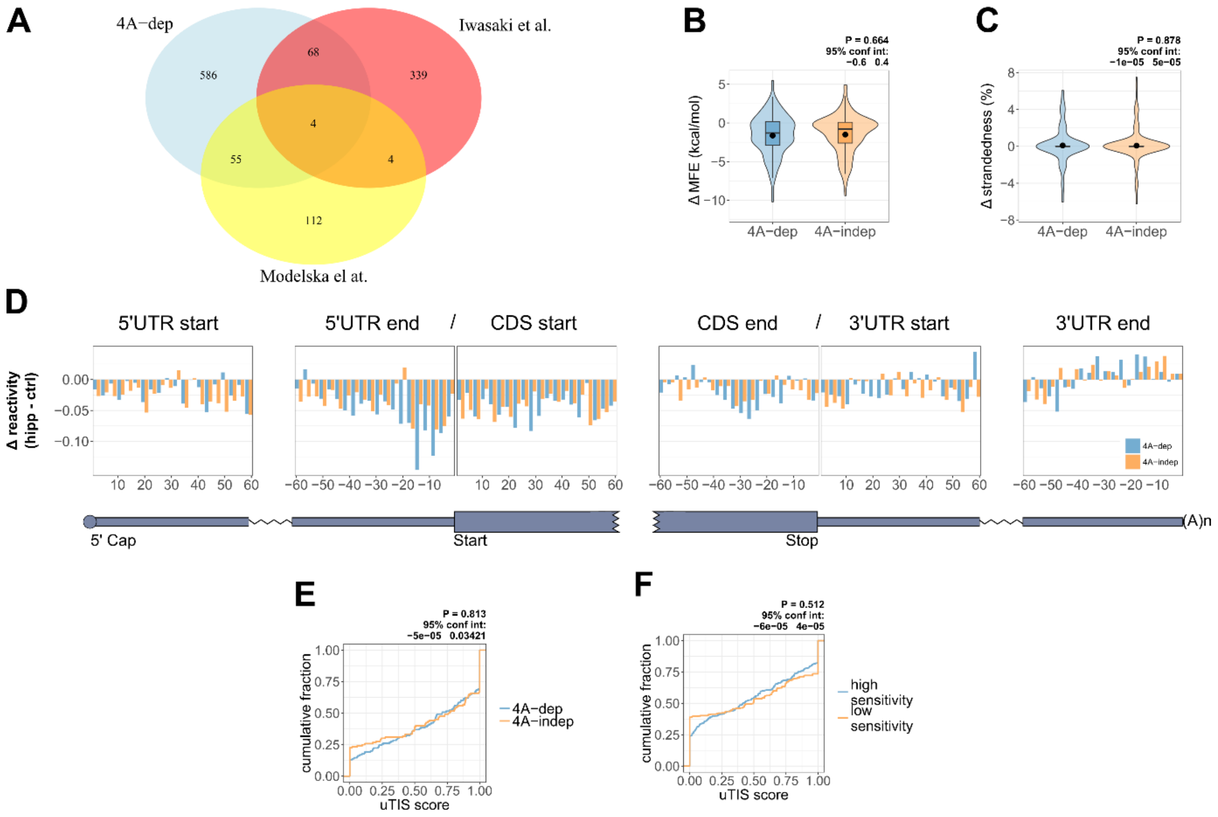

Figure S7. eIF4A-dependent mRNAs gain in structure upon hippuristanol treatment immediately upstream of the CDS. (A) Venn diagram depicting the overlap in eIF4A-dependent mRNAs identified in this study and hipp-sensitive mRNAs identified by Iwasaki *et al.* (2016) (33) and eIF4A1-dependent mRNAs identified by Modelska *et al.* (2015) (4). **(B-C)** Violin plot depicting Δ MFE for all 100nt windows from Figure S4E, and Δ strandedness, for all 100nt windows from Figure S4G, for the same eIF4A-dependent and independent mRNAs included in Figures 4A-C. P values and 95% confidence intervals were calculated using an un-paired, two-sided Wilcoxon test. **(D)** Average Δ reactivity (hipp – control) for the first and last 60nt of UTRs and CDS for all eIF4A-dependent and independent mRNAs included in Figure 4D. **(E)** Cumulative distribution function plot of upstream translation initiation sites (uTIS) scores for the eIF4A-dependent and independent mRNAs from panel D. **(F)** Cumulative distribution function plot of uTIS scores for the high sensitivity (eIF4A-dependent) (n = 949) and low sensitivity (eIF4A-independent) (n = 606) mRNAs following 1 μM hippuristanol treatment taken from Iwasaki *et al.* (2016) (33). uTIS scores were calculated by dividing the number of reads mapped to upstream start sites by the number of reads mapped to both upstream and the annotated start sites based on data from Lee *et al.* (2012) (43). P values and 95% confidence intervals were calculated using an un-paired, two-sided Wilcoxon test.

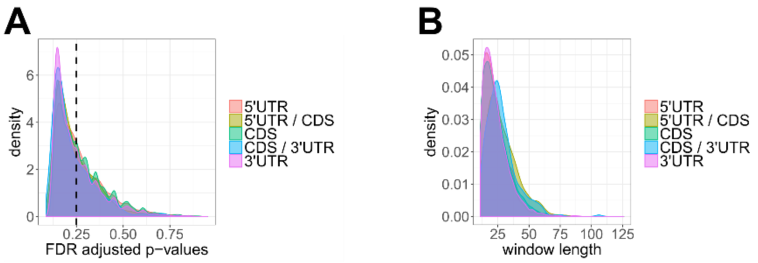

Figure S8. dStruct FDR adjusted p-values and window sizes. (A) False discovery rate (FDR) adjusted p-values of all windows analysed by dStruct (50). **(B)** Window sizes of all windows with a FDR adjusted p-value less than 0.25, identified by dStruct.

Sample	Raw reads	Trimmed reads	% passing filter	Mapped reads	% mapped	Unique reads	% unique	multi-map reads	% multi-map
Control/DMS(-)	289943349	286967310	99.0	251576930	87.7	38678168	15.4	212898762	84.6
Control/DMS(+)	320135544	317327469	99.1	287458000	90.6	35213924	12.3	252244076	87.7
Hippuristanol/DMS(-)	310243439	306762445	98.9	268963086	87.7	41676969	15.5	227286117	84.5
Hippuristanol/DMS(+)	348418102	344944909	99.0	312188481	90.5	38767835	12.4	273420646	87.6
**Total**	**1268740434**	**1256002133**	**99.0**	**1120186497**	**89.1**	**154336896**	**13.9**	**965849601**	**86.1**

Table S1. Summary of sequencing reads obtained for Structure-seq2

Figure	Panel	StructureFold2 Script	R Script
1	C	react_to_csv.py	Positional_changes.R
	F-G	react_windows.py<br>batch_fold_RNA.py<br>fasta_composition.py<br>structure_statistics.py	Sliding_windows_folded.R
2	A	-	Polysome_scatter_plots.R
	B-D	react_statistics.py	Stats_plots.R
	E	react_to_csv.py	Positional_changes.R
3	A	-	Polysome_scatter_plots.R
	B-E	fasta_compositions.py	Polysome_feature_properties.R
	G-J	react_static_motif.py<br>react_statistics.py	GGC4.R
4	A-C	react_statistics.py	Stats_plots.R
	D	react_to_csv.py	Positional_changes.R
5	B-H	react_windows.py	Sliding_windows_reactivity.R
6	A-F	-	dStruct.R
S1	F-G	rtsc_specificity.py<br>check_ligation_bias.py	Specificity_and_ligation_bias.R
S2	A-C	rtsc_correlation.py	Replicate_correlation.R
S3	A-C	rtsc_end_coverage.py	End_coverage.R
S4	A-J	react_statistics.py	Stats_plots.R
S5	A-C, G-H	react_to_csv.py	Positional_changes.R
S6	A	react_to_csv.py	Positional_changes.R
	B	-	uTIS_scores.R
S7	A	-	Overlaps.R
	B-C	react_statistics.py	Stats_plots.R
	D	react_to_csv.py	Positional_changes.R
	E-F	-	uTIS_scores.R
S8	A-B	-	dStruct.R

Table S2. Summary of scripts used to create each figure. All R scripts are available from GitHub using the following link https://github.com/Bushell-lab/Structure-seq2-with-hippuristanol-treatment-in-MCF7-cells. All StructureFold2 scripts are available from GitHub using the following link https://github.com/StructureFold2/StructureFold2.
