# Supplementary material for: mRNA structural elements immediately upstream of the start codon dictate dependence upon eIF4A helicase activity
Source: Genome Biol. 2019 Dec 30;20:300. doi: 10.1186/s13059-019-1901-2 (PMC6936103; doi:10.1186/s13059-019-1901-2)
Supplement: Supplementary file 2 — Additional file 2. Review history. [file 13059_2019_1901_MOESM2_ESM.docx]

Review History

**First round of review**

**Reviewer 1**

**Are you able to assess all statistics in the manuscript, including the appropriateness of statistical tests used?**

Yes, and I have assessed the statistics in my report.

**Comments to author:**

Waldron et al. report a transcriptome-wide study aiming to elucidate the role of the eIF4A1 DEAD box RNA helicase in translation control. They focus on eIF4A1's interactions with 5' UTRs of mRNAs it regulates, testing a hypothesis that these UTRs are more structured than those in eIF4A1-independent mRNAs and therefore require helicase activity to facilitate translation. To this end, they use high-throughput RNA structure probing experiments, which gauge structures in vivo, to perform a comparative meta-gene structural analysis between the active and inhibited states of the helicase. The findings are compared to polysome profiling experiments in order to link structure to function (i.e., translation enhancement). These analyses establish an association between 5' UTR structure remodeling and eIF4A1-RNA interactions, particularly at the 3' end of the UTRs.

The manuscript is well written and the work is well presented and motivated. My concerns center on the analysis, which could and should be improved. Briefly, since datasets of the type analyzed here are very noisy, more stringent coverage criteria should have been used and replicate information should have been utilized better. The search for reactivity differences could also be simplified and better performed by using more refined available analysis methods. Currently, I am not fully convinced that some of the results reflect real biological effects. Below, I summarized my main concerns and a few related questions.

Major Comments

- Coverage: this is a major issue in transcriptome-wide structure analysis and is especially important in the context of comparative studies. If I understand correctly, mRNAs were analyzed if they have 1 or more stops per A/C on average. I think this threshold is way too low for any reactivity analysis but especially when considering very noisy in vivo probing data. One can see in Fig. 1SH the poor reproducibility of stop counts at low coverages. For comparison, Mostoe et al. (Cell, 2018) use thresholds near 1,000. In my experience, thresholds in the range 100-300 often suffice for reasonable-to-good quality data and reproducible analysis. The authors could/should also test the reproducibility of their analyses across the 3 replicates to determine a satisfactory threshold. They may also want to consider averaging reactivities across the 3 replicates to reduce the noise prior to any analysis.

- Replicates: three replicates were obtained for each condition but were merged into one dataset. This means that information on biological variation within a condition was lost, which is fundamental to differential analysis. Here, the authors used meta-gene analyses to gain statistical confidence from large samples, but in several comparisons they make between conditions, it is unclear to me if the observed differences represent a differential signal or just noise because I don't know what the noise level is. See more comments below.

Additionally, replicates can be used to smooth the noise by averaging the reactivities prior to any meta-analysis, which was not done. I understand there is a tradeoff between replicates and coverage, but to me, the results would be more convincing if fewer transcripts were analyzed but noise had been quantified and reduced via more stringent coverage filtering and usage of replicate information.

- The authors present preliminary differential reactivity analysis by averaging reactivities across very long regions (Fig. 1C-E, CDS + UTRs). As they note, differences are small and it seems that their averaging strategy masks the differential signal by smoothing over local "up" and "down" changes. I have several concerns regarding this analysis and suggest moving it to the Supp.

First, I disagree with the authors that their results are surprising (page 7). It is well accepted that local perturbations to structure may propagate into nearby regions to form new local MFE conformations. Second, it makes more sense to average over very small regions (e.g., Smola et al., Biochemistry, 2015, Wan et al., Nature, 2014) because protein-induced changes are often local and will cancel out when averaged. Indeed, most differential DMS/SHAPE analysis methods test for local changes over short windows. Third, and most importantly, the differences in Fig. 1C-E seem really small and given that in vivo structure data are noisy, they might manifest noise or biological variation. Since the authors generated 3 replicates, I suggest they repeat their analysis for each pair of replicates. For example, if one compares 5' UTRs between replicates 1 and 2 instead of control and hipp, is the difference statistically significant? If yes, then we're detecting noise. Fourth, computationally folding full length 5' UTRs might be unreliable because UTRs are often too long to be accurately predicted by MFE algorithms. What is the length distribution of these UTRs?

- The differences shown for the Gini index analysis (Fig. S3C-E) seem more convincing than in the averaging analysis (Fig. 1C-E). However, I still recommend using the 3 replicates to repeat the same test as suggested above to validate what the test is picking up. Note also that Gini index is susceptible to outliers and given the low coverage used in this analysis, these differences might represent analysis artifacts.

- As a general comment regarding the entire differential DMS analysis, I think a simpler and more robust way to go about it is to search for local changes in well-covered transcripts using an existing diff reactivity analysis method and also make use of the 3 replicates in this search instead of merging them. Here, the authors used a meta-gene approach to gain statistical confidence, but 3 replicates and sufficiently deep coverage should also provide reasonable confidence for analysis at the transcript level. I also expect methods that do not average reactivities to outperform those that do.

- CLIP data: one component that is missing is CLIP data for eIF4A1. It could, for example, reveal if eIF4A1 binds primarily near the 3' end of 5' UTRs or not. It is also natural to seek correlation between binding sites and sites of structural remodeling, as has been done in prior studies of DEAD-box helicases (see comment on prior literature below). As another example, the authors discuss potential causes of structural changes in the CDS (Fig. 1D), including eIF4A1 binding in this region. This is something that CLIP experiments can potentially clarify. Is there a reason why CLIP experiments were not analyzed? While I don't expect the authors to add experiments, it may be worth looking into existing data, e.g., uvCLIP by Maticzka et al., Nature Comm, 2018, although for a different cell line.

Minor Comments

- Prior literature: there are several recent papers that report similar transcriptome-wide in vivo studies of DEAD-box helicases using DMS/SHAPE to elucidate their roles. For example, Lee et al., Genes & Development, 2018 (DDX5 splicing targets), Lai et al., Genetics, 2019 (Dbp2 targets and transcription control), and particularly Guenther et al., Nature, 2018 (Ded1p role in translation control). Similarly to this manuscript, these studies performed integrated analyses of structure and/or translation datasets but also analyzed protein-binding data.

- In the Introduction, the authors point out an important limitation of in vivo structure probing, i.e., low reactivity might result from double-strandedness or from protein protection, but it's generally impossible to tell the true cause. Since this is the case for some protein-reagent pairs, I am wondering if information is available on the eIF4A1-RNA binding interface. For example, is it known whether unpaired nucleotides in the binding site are accessible to DMS? Is it known if the binding involves the backbone or the WC face of participating nucleotides? Such knowledge often guides the choice of probing reagent and may also increase our confidence that at least the DMS differences observed with/without eIF4A1 have some structural basis. Of course, the (very modest) decrease in avg. reactivity observed upon eIF4A1 inhibition supports that eIF4A1 does not block DMS, but I still think it would be good to clarify this point, at least from the perspective of study/experiment design.

- Fig. 2A: results look convincing, but I still wonder how a meta-analysis of the "delta reactivity" looks like upon replacement of "control" and "hipp" by replicates i and j. The 95% confidence intervals may also shrink once stricter coverage criteria are applied, which can strengthen the results. Note that I am only referring to the diff analysis panel and not the trend itself, which seems robust.

- Fig. 2E: units of y axis?

- Fig. 3 and 4: quality is poor.

- Fig. 3E: similar comments as in Fig. 2A, especially when it comes to coverage.

- 5' UTR analysis on p. 8-9: a 25 nt window seems too small for computational MFE predictions. Genomic screens that employ local folding typically use window sizes in the 100-150 range. See, for example, RNAz by the Vienna group.

- Page 11: I couldn't find the details of the Bayesian model in Methods.

- Fig. 6B-C: is the y axis scaled logarithmically?

- Page 14 and Fig. 6: as mentioned above, averaging reactivities over regions essentially loses the differential signal. While it should work better for very small regions, it could still easily miss changes in signal pattern that are characteristic of structure remodeling. I think this analysis demonstrates this point. Performing it with a more refined approach, on a transcript-by-transcript basis, and using replicates to account for biological variation could strengthen these results. Also, how were the "sequences with the biggest decrease or increase in reactivity" determined? Do they need to exceed a certain threshold or does one choose a pre-set percentile?

- Fig. 6F and p. 15: I don't consider r = 0.27 to be indicative of correlation, but this may improve following stricter coverage screening.

- Sequencing and bioinformatic pipeline, p. 25: the authors deal with multiple alignments to transcript variants by mapping reads to a gene's most abundant transcript. However, when several variants are abundant, they could easily adopt different structures, at least within the CDS, and this would result in reactivity misestimation due to misalignment. If the fraction of such transcripts is not too large, I suggest excluding them from analysis. Otherwise, this potential issue should be noted in the text.

- Correlation of GC content with DMS reactivity: this is an interesting analysis but a potential caveat is a known A/C bias in stop-based DMS data, where modifications are more detectable in A than in C. This bias is protocol-dependent and can be indirectly deduced from Fig. 1SF but it is not straightforward to quantify because C is also more likely to pair than A. A possible way to account for this is to limit reactivity analysis to C only and compare C reactivity distributions between different UTR locations or at the least point out this issue.

**Reviewer 2**

**Are you able to assess all statistics in the manuscript, including the appropriateness of statistical tests used?**

No, I do not feel adequately qualified to assess the statistics.

**Comments to author:**

This study assesses the role of eIF4A in translation initiation and 5'UTR structure in the human breast cancer cell line MCF7. This is an absolutely key question in the field of translation control; where the current dogma is the helicase function of eIF4A acts to promote both recruitment of the small ribosomal subunit to the mRNA and subsequent scanning by this subunit. Both functions have been proposed to rely upon the unwinding activity of eIF4A as part of translation initiation complex. eIF4A can also serve as a regulated step in the translation process and targeting eIF4A is a potential therapeutic strategy for various human conditions.

In this paper, RNA structure has been assessed by mapping DMS reactive regions of RNA across the transcriptome. These results have then been correlated with various other datasets including a polysome enrichment dataset in the same cells. The authors conclude that eIF4A is involved in remodelling 5'UTR structures such that inhibiting its function with hippuristanol leads to local decreases and increases in DMS reactivity. Intriguingly inhibition of eIF4A leads to decreased DMS reactivity in a region just upstream of the Start codon, and mRNAs that are most translationally sensitive to eIF4A inhibition also have decreased DMS reactivity in this region. This supports a model where the helicase function of eIF4A is particularly important in remodelling the region upstream of mRNA start codon during the scanning process. I feel this paper makes a highly valuable contribution to an area of literature by taking a global approach to the study of question that has in the past been dominated by single mRNA reporter gene style studies.

1. I have a couple a very minor comments on the introduction:-

a. I feel the authors should state somewhere that eIF4A1 is an ATPase and while it can function as an helicase - relative to other RNA helicases this activity is weak. So other possibilities exist for instance eIF4A could remodel protein/ RNA complexes and has been suggested to influence the 40S complexfacilitating mRNA interaction (e.g. Sokabe and Fraser, 2017: Yourik et al., 2017)

b. The authors state the excess of eIF4A over other translation factors and assume that this 'excess' is 'free' eIF4A. It could be that many eIF4A molecules are involved in the translation process and therefore the stoichiometry of eIF4A involvement may not permit 'free' eIF4A?

2. For the polysome gradients, I have never seen the 48S peak separated from the 40S peak on standard sucrose gradients. In Figure 1B the authors label a peak as 48S. I suspect this is the 60S peak? This is often observed as a shoulder on the 80S peak. So I would interpret this gradient as 40S/48S, then 60S as a shoulder on the 80S. To support their interpretation the authors would need to conduct western/ northern blots investigating the ribosomal proteins or rRNA present in different fractions taken from the gradient.

Critically in my view, where 48S accumulation has been observed by others in the field, the most obvious feature on polysomes is halfmers, because the 40S and 48S do not separate well and most stalled 43S ribosomes are on mRNAs that already carry polysomes. So if the authors' interpretation is correct and 43S complexes are in some way accumulating on the mRNA- why do they not see halfmers?

3. P5 line 22 - the authors need to explain in more detail why the cited Figures support the interpretation that their libraries are high quality.

a. Related to this - in the analysis shown in Figure S1H the authors show plots comparing biological replicates to each other. Ideally they should report the r-value for comparison across non-replicates i.e. are the r-values reported better between replicates than across samples generally and how much better are they?

b. Again related to data quality - the authors state in the methods that 'the reads from each replicate were pooled'. This begs the immediate question- what is the point of doing the replicates as this information is now lost in the pooled analysis?

4. P6 lines 14-17- given what is stated, the authors should probably not refer to the region lacking the final 125n as the 3'end, maybe 3' region would be a better term?

5. P6 and 7. The authors conclude that the reduction in DMS reactivity over the CDS after eIF4A inhibition is due to the absence of the ribosomes which would normally unwind secondary structure. They should explain more clearly that the decreased reactivity in the 5'UTR is unlikely due to the observed accumulation of 43S complexes, and indeed this may explain increased reactivity in certain areas of the 5'UTR

6. P8 line 16-18. I wonder if there is a precedent that the authors could cite for DMS reactivity being almost immune to protein or ribosome binding?

7. P9 line 11. It seems very odd to use a HEK293 dataset here - especially when later in the paper the authors state that transcripts vary significantly between HEKs and MCF7 cells. Perhaps the authors could add a sentence here explaining why the approach is a valid one.

8. P9 line 16 'strongly argues' should be toned down just 'argues' is sufficient.

9. P10 lines 1-8, is quite confusingly written - initially it was not clear whether the authors had performed a ribosome density mapping technique (ribosome profiling), or taken a polysome enrichment approach. They should make this clearer and justify their approach in the text. How do these data compare with the data from Iwasaki et al 2016 using HEK293 cells and Rocaglates. There should be some correlation?

10. P10- In the last paragraph the logic is difficult to follow. The authors present two possible explanations but then how the experiments relate to these possibilities is difficult to understand. Plus they us the phrase "To rule out the possible explanation"- this should be something like to test, assess or distinguish.

11. P11- Line 4 "excludes the possibility" is too strong here - there are too many caveats - for instance if I understand correctly the data being compared are from different cell lines- so the conclusion can never be unequivocal.

12. P11- Line 19 typo- should be that instead of than?

13. In the discussion I would like to see the authors place their observations into a more holistic view of the role of RNA helicases in translation initiation - key things to add would be:-

1. the role of other helicases - for example DHX29, Ded1/DDX3, Vasa/DDX4, RNA helicase A (RHA/DHX9), and Dhh1/RCK/DDX6

2. Potential roles of eIF4A in ribosome recruitment - possibly by remodelling the 40S subunit?

**Authors Response**

**Point-by-point responses to the reviewers’ comments:**

Reviewer #1:

Waldron et al. report a transcriptome-wide study aiming to elucidate the role of the eIF4A1 DEAD box RNA helicase in translation control. They focus on eIF4A1's interactions with 5' UTRs of mRNAs it regulates, testing a hypothesis that these UTRs are more structured than those in eIF4A1-independent mRNAs and therefore require helicase activity to facilitate translation. To this end, they use high-throughput RNA structure probing experiments, which gauge structures in vivo, to perform a comparative meta-gene structural analysis between the active and inhibited states of the helicase. The findings are compared to polysome profiling experiments in order to link structure to function (i.e., translation enhancement). These analyses establish an association between 5' UTR structure remodeling and eIF4A1-RNA interactions, particularly at the 3' end of the UTRs.

The manuscript is well written and the work is well presented and motivated. My concerns center on the analysis, which could and should be improved. Briefly, since datasets of the type analyzed here are very noisy, more stringent coverage criteria should have been used and replicate information should have been utilized better. The search for reactivity differences could also be simplified and better performed by using more refined available analysis methods. Currently, I am not fully convinced that some of the results reflect real biological effects. Below, I summarized my main concerns and a few related questions.

*We thank this reviewer for their careful reading of the manuscript and for the important points they’ve raised and their suggestions to improve the manuscript. We felt that the main overriding issue, which was also brought up by Reviewer #2, was the fact that we have pooled the reads from the replicates and hence lost potentially important information that can be obtained from the variation between replicates. To address this issue we have used the dStruct package (1), which identifies differentially reactive windows, taking into account the information from biological replicates. The data obtained from this analysis strongly supports our take home message, that increased structure in the 3’ end of 5’UTRs confers increased dependency on eIF4A activity for translation. We address each individual comment in detail below.*

Major Comments

1) Coverage: this is a major issue in transcriptome-wide structure analysis and is especially important in the context of comparative studies. If I understand correctly, mRNAs were analyzed if they have 1 or more stops per A/C on average. I think this threshold is way too low for any reactivity analysis but especially when considering very noisy in vivo probing data. One can see in Fig. 1SH the poor reproducibility of stop counts at low coverages. For comparison, Mostoe et al. (Cell, 2018) use thresholds near 1,000. In my experience, thresholds in the range 100-300 often suffice for reasonable-to-good quality data and reproducible analysis. The authors could/should also test the reproducibility of their analyses across the 3 replicates to determine a satisfactory threshold.

*We agree with the reviewer that increasing the coverage would reduce noise and increase replicate correlation and we have made every effort to do so. To address this issue we have calculated coverage for each individual replicate, as opposed to for the pooled reads, therefore ensuring that transcripts are well covered in each replicate. We determined a suitable coverage threshold by assessing the reproducibility across the 3 replicates, as requested by the reviewer, by plotting the distribution of correlation coefficients for every transcript, following filtering at coverage thresholds of 0, 1, 10 and 100 within every replicate (Figure S2B). We believe this shows that a threshold of one within each replicate is a suitable trade-off between increased accuracy and the number of transcripts, as increasing coverage beyond this dramatically reduces the number of transcripts, without significantly improving the correlation coefficients. While we realise this is below the thresholds requested by the reviewer, we argue that we have increased it to the viable limit within these data and we feel the comparison with Mustoe et al. is not directly applicable for the following reasons.*

*In Mustoe et al. the reactivity is measured by mutational profiling, where the reverse transcriptase reads through the sites of modification, inserting a random nucleotide, as opposed to our data where we measure reactivity by detecting reverse transcriptase stops. The way that coverage is calculated is therefore entirely different. For example, a 1KB mRNA with 50% AC content and 1000 reads would have a coverage of 2 within our analysis (number of reads / (length of mRNA x AC content)). However, for mutational profiling, coverage is calculated for each nucleotide. The average coverage of every nucleotide within a transcript would therefore be (read length x number of reads) / length of mRNA. Therefore, with read lengths of 150 the same mRNA would have an average coverage of 150 for each nucleotide (75 x higher). Note that DMS/SHAPE treatment carried out at single-hit kinetics should have only one modification for every 100-200nt, so although the calculated coverage would be much higher, there would not be more DMS/SHAPE reagent derived modifications within this mRNA. Our new coverage threshold of one within every replicate is more than three times what we were using previously and considering that the coverage obtained from mutational profiling would be roughly 75x higher, this would be comparable to a mutational profiling coverage threshold of 225 (3 x 75). Also, the data from Mustoe et al. is obtained from bacteria, which obviously has a much smaller transcriptome than human, meaning the number of reads per transcript will be much higher, for the same amount of total reads.*

*We are also restricted in the amount by which we can increase the coverage threshold, due to the additional stringent filtering that we have imposed on our analysis. For example, in Figure S3 (previously S2) we highlight the poor annotation of 5’ ends within the commonly used RefSeq transcriptome, which would be a big concern for us as we are particularly interested in 5’UTRs. We therefore have used an MCF7 specific transcriptome, which we show in Figure S3 is significantly more accurate. However, this transcriptome does not have translation start and stop sites annotated, meaning that we had to manually curate these ourselves, by blasting the manually curated RefSeq coding sequences against the MCF7 specific transcriptome. Of the 55,770 transcripts in the MCF7 transcriptome, we were only able to confidently annotate both the translation start and stop sites on 13,132 transcripts. Furthermore, we further filtered these transcripts by a 5’ end coverage score (Figure S3), which we developed specifically for this purpose, but which results in a further loss of roughly 50% of transcripts. (Note that the transcript numbers in Figure S2B are prior to these additional filtering steps.)*

*To summarise, we are now filtering by coverage of one within every replicate. By doing this we have effectively increased our coverage threshold by a factor of more than three, as well as ensuring every transcript used is well covered in every replicate. We show that at this coverage threshold we have good reproducibility between replicates, both at the transcript level (Figure S2B) and also across the whole transcriptome (Figure S2C). To compare transcript numbers, for Figure 1C (previously 2A) we now have 1,266 mRNAs, compared to the 2,157 previously, and for Figure 4C (previously 5C) we now have 147 eIF4A-dependent mRNAs, compared to the 208 previously. As the main aspect of this paper is to compare changes in DMS reactivity between eIF4A-dependent and eIF4A-independent mRNAs, increasing coverage beyond this new limit would not leave enough transcripts for any analysis to be carried out between these two groups of transcripts. We therefore feel we have increased the threshold to its viable limit within these data.*

2) They may also want to consider averaging reactivities across the 3 replicates to reduce the noise prior to any analysis.

*We are thankful for the suggestion to average reactivities across the 3 replicates, which we have now done instead of calculating reactivity after pooling the reads. All R scripts used to generate the figures have been re-written to use averaged reactivities.*

3) Replicates: three replicates were obtained for each condition but were merged into one dataset. This means that information on biological variation within a condition was lost, which is fundamental to differential analysis. Here, the authors used meta-gene analyses to gain statistical confidence from large samples, but in several comparisons they make between conditions, it is unclear to me if the observed differences represent a differential signal or just noise because I don't know what the noise level is. See more comments below.

*We agree that using the information from the three replicates would make the analysis more robust. We have therefore made use of the dStruct package (1) to address this, described in more detail to comment 7 below.*

4) Additionally, replicates can be used to smooth the noise by averaging the reactivities prior to any meta-analysis, which was not done. I understand there is a tradeoff between replicates and coverage, but to me, the results would be more convincing if fewer transcripts were analyzed but noise had been quantified and reduced via more stringent coverage filtering and usage of replicate information.

*We have averaged the reactivities prior to any meta-analysis as requested and detailed our response to increasing coverage thresholds above (comment 1).*

5) The authors present preliminary differential reactivity analysis by averaging reactivities across very long regions (Fig. 1C-E, CDS + UTRs). As they note, differences are small and it seems that their averaging strategy masks the differential signal by smoothing over local "up" and "down" changes. I have several concerns regarding this analysis and suggest moving it to the Supp.

a) First, I disagree with the authors that their results are surprising (page 7). It is well accepted that local perturbations to structure may propagate into nearby regions to form new local MFE conformations.

*While we agree that local changes in structure may well cause alterations in nearby regions, which our data strongly support, the reason we plotted the average changes in the 5’UTRs/CDSs/3’UTRs was to calculate whether overall these alterations in structure balanced out, or whether on average certain regions became more or less structured. Also, while many in the RNA structure field would agree with this point, many in the translation field will find it surprising that 5’UTRs don’t become significantly more globally structured following eIF4A inhibition. We have altered the text on p7 lines 23-26, so that this comes across better and removed the word “surprising” from the manuscript.*

b) Second, it makes more sense to average over very small regions (e.g., Smola et al., Biochemistry, 2015, Wan et al., Nature, 2014) because protein-induced changes are often local and will cancel out when averaged. Indeed, most differential DMS/SHAPE analysis methods test for local changes over short windows.

*This is a good point. In addition to the sliding window analysis already in the manuscript (Figure 5), we have added our analysis using the dStruct package (Figures 6 and S8), which identifies differentially reactive windows and is discussed in more detail to comment 7 below.*

c) Third, and most importantly, the differences in Fig. 1C-E seem really small and given that in vivo structure data are noisy, they might manifest noise or biological variation. Since the authors generated 3 replicates, I suggest they repeat their analysis for each pair of replicates. For example, if one compares 5' UTRs between replicates 1 and 2 instead of control and hipp, is the difference statistically significant? If yes, then we're detecting noise.

*To address the issue of biological variation we have used the dStruct package, which takes variation between replicates into account when identifying differentially reactive windows, as outlined in more detail to our response to comment 7 below. Upon averaging the reactivities and increasing the coverage threshold as requested, the 95% confidence intervals become larger, whereas you would expect the opposite if the differences reflected noise. We would like to keep these plots in the manuscript, as they show that the overall trend within three replicates is for the CDSs to gain in average structure the most, followed by the 5’UTR, and then the 3’UTR, which we think is an important finding. However we accept that they may not merit such prominence and so we have moved them to the supplemental information (Figure S4A-C).*

d) Fourth, computationally folding full length 5' UTRs might be unreliable because UTRs are often too long to be accurately predicted by MFE algorithms. What is the length distribution of these UTRs?

*This is a very good point. Therefore instead of folding full length 5’UTRs we have folded windows of 100nt with 10nt steps for all transcripts with 5’UTRs more than 100nt in length. For all transcripts with 5’UTRs shorter than 100nt we have folded the full length 5’UTR. We have then plotted the minimum and average MFE for all windows per transcript (Figures S4D-E). The 5’UTR length distribution is already included in Figure 3B.*

6) The differences shown for the Gini index analysis (Fig. S3C-E) seem more convincing than in the averaging analysis (Fig. 1C-E). However, I still recommend using the 3 replicates to repeat the same test as suggested above to validate what the test is picking up. Note also that Gini index is susceptible to outliers and given the low coverage used in this analysis, these differences might represent analysis artifacts.

*Similar to the average reactivity changes (see comment 5C), the differences in Gini coefficients between control and hippuristanol increased following more stringent coverage thresholds and averaging reactivities (Figure S4H-J).*

7) As a general comment regarding the entire differential DMS analysis, I think a simpler and more robust way to go about it is to search for local changes in well-covered transcripts using an existing diff reactivity analysis method and also make use of the 3 replicates in this search instead of merging them. Here, the authors used a meta-gene approach to gain statistical confidence, but 3 replicates and sufficiently deep coverage should also provide reasonable confidence for analysis at the transcript level. I also expect methods that do not average reactivities to outperform those that do.

*To search for differentially reactive windows, we have used the dStruct package (1), which identifies windows that change in reactivity pattern more between control and hippuristanol samples than between replicates. This package searches for local changes in reactivity and applies statistical testing to ensure the changes between control and hippuristanol are robust. In brief it calculates dissimilarity (d) scores, and identifies windows which have higher d-between scores (between control and treated samples), than d-within scores (between replicates). The analysis is not restricted to identifying changes in average reactivity within regions, as it instead identifies windows that have changed in reactivity pattern, such as would occur during the re-folding of a hairpin, with certain nucleotides increasing and others decreasing in reactivity.*

*This analysis strongly supports the main findings of this manuscript, in that only in the 5’UTR do we see a statistically significant difference in the Δ reactivity of these windows between eIF4A-dependent and independent transcripts (Figure 6B) and crucially, when we bin the Δ reactivities across the length of the transcript, we again see a strong decrease in reactivity at the 3’ end of the 5’UTRs, only in eIF4A-dependent transcripts (Figure 6D). The complete data from these analyses are included in Figure 6 and Figure S8 of the revised manuscript and text is added on p18 and 19. Many thanks for this suggestion as we really think this has strengthened the manuscript.*

8) CLIP data: one component that is missing is CLIP data for eIF4A1. It could, for example, reveal if eIF4A1 binds primarily near the 3' end of 5' UTRs or not. It is also natural to seek correlation between binding sites and sites of structural remodeling, as has been done in prior studies of DEAD-box helicases (see comment on prior literature below). As another example, the authors discuss potential causes of structural changes in the CDS (Fig. 1D), including eIF4A1 binding in this region. This is something that CLIP experiments can potentially clarify. Is there a reason why CLIP experiments were not analyzed? While I don't expect the authors to add experiments, it may be worth looking into existing data, e.g., uvCLIP by Maticzka et al., Nature Comm, 2018, although for a different cell line.

*We thank the reviewer for pointing us towards the uvCLAP data from Maticzka et al. (Nature Comm, 2018), which we have now analysed. Unfortunately the coverage of the data is very poor. For eIF4A1 there are only 26 peaks found in the 5'UTR (of a total of 42 across the mRNA) and this corresponds to peaks on just 10 mRNAs. When we look at these mRNAs in HEK293 total RNA-seq data from our lab we see these 10 mRNAs are also highly abundant mRNAs (4/10 in the top 1% of expressed mRNAs, 7/10 in the top 10%), again showing the uvCLAP data lacks sequence coverage. Unfortunately, this dataset is therefore not of sufficient quality for us to use in any analysis to be able to make global conclusions about the precise position of eIF4A1 in relation to the Structure-seq data.*

*However, in support of our findings, we would like to mention that unpublished data recently presented at scientific meetings from the Tollervey (Edinburgh) and Willis (Cambridge) labs, have identified highly enriched eIF4A and eIF4B binding sites close to the start codon, through cross-linking IP sequencing techniques in both yeast and mammalian cells, respectively.*

Minor Comments

9) Prior literature: there are several recent papers that report similar transcriptome-wide in vivo studies of DEAD-box helicases using DMS/SHAPE to elucidate their roles. For example, Lee et al., Genes & Development, 2018 (DDX5 splicing targets), Lai et al., Genetics, 2019 (Dbp2 targets and transcription control), and particularly Guenther et al., Nature, 2018 (Ded1p role in translation control). Similarly to this manuscript, these studies performed integrated analyses of structure and/or translation datasets but also analyzed protein-binding data.

*We thank the reviewer for pointing out these papers. We were well aware of the Guenther et al. study and apologise for not citing this in the original submission as it was our intention to do so. All three papers have now been acknowledged in the text on p4 lines 11-12.*

10) In the Introduction, the authors point out an important limitation of in vivo structure probing, i.e., low reactivity might result from double-strandedness or from protein protection, but it's generally impossible to tell the true cause. Since this is the case for some protein-reagent pairs, I am wondering if information is available on the eIF4A1-RNA binding interface. For example, is it known whether unpaired nucleotides in the binding site are accessible to DMS? Is it known if the binding involves the backbone or the WC face of participating nucleotides? Such knowledge often guides the choice of probing reagent and may also increase our confidence that at least the DMS differences observed with/without eIF4A1 have some structural basis. Of course, the (very modest) decrease in avg. reactivity observed upon eIF4A1 inhibition supports that eIF4A1 does not block DMS, but I still think it would be good to clarify this point, at least from the perspective of study/experiment design.

*This is an important point, and one that did guide our choice in using DMS rather than SHAPE reagents as eIF4A is known to bind the RNA backbone. We have now added this to the manuscript on p4 lines 18/19.*

11) Fig. 2A: results look convincing, but I still wonder how a meta-analysis of the "delta reactivity" looks like upon replacement of "control" and "hipp" by replicates i and j. The 95% confidence intervals may also shrink once stricter coverage criteria are applied, which can strengthen the results. Note that I am only referring to the diff analysis panel and not the trend itself, which seems robust.

*We believe the dStruct analysis strongly supports panel 2A (now 1C) and particularly panel 4D (previously 5D) that the biggest increases in structure in eIF4A-dependent mRNAs following eIF4A inhibition are towards the 3’ of the 5’UTR. Also, the revised figure (Figure 1C) is plotted with the increased coverage thresholds and averaged reactivities, which has slightly improved the overall pattern and 95% confidence intervals, as predicted by the reviewer. The only difference in the pattern is that we no longer see the strong increase in reactivity at the 3’ end of the 3’UTRs, so we have removed lines 1-4 from p11 to reflect this.*

12) Fig. 2E: units of y axis?

*The units of y axis is kcal/mol and has been added to the figure along with Figures S4D-E and S7B. We would like to thank the reviewer in pointing out this mistake to us.*

13) Fig. 3 and 4: quality is poor.

*We apologise for the poor quality of these figures. The original images are of good quality but appear to have been distorted during submission. These should now be of good quality in the revised upload.*

14) Fig. 3E: similar comments as in Fig. 2A, especially when it comes to coverage.

*These plots have also been re-made using the increased coverage and averaged reactivities, although due to reduced numbers we have now selected the top and bottom third of mRNAs as opposed to 20% used previously. The overall findings remains consistent, although out of all the figures, this is the one that has changed the most following the increased coverage and averaging of reactivities. Whereas before it was only in the last 20-30% of the 5’UTR that the high TE mRNAs became more reactive, this now occurs further 5’ in the amended figures. Also within the CDS the biggest difference is towards the 3’ end, where the high TE group is more reactive and there is a robust difference across the length of the 3’UTR, with the low TE mRNAs being more reactive in this region. The average changes in the violin plots have also changed slightly. We have modified the text to reflect these changes on p11 and 12.*

15) 5' UTR analysis on p. 8-9: a 25 nt window seems too small for computational MFE predictions. Genomic screens that employ local folding typically use window sizes in the 100-150 range. See, for example, RNAz by the Vienna group.

*While we appreciate the reviewer’s comments here, it would not be possible to bin the data with windows of 100nt within the 5’UTR due to 5’UTR length constraints. However, we take the point and have repeated the analysis using 50nt windows, which does not change the message (Figure 1G), and which we hope the reviewer finds satisfactory. Also, this is not an issue for GC content (Figure 1F), which also supports this data in that it mirrors the pattern we see in MFE.*

16) Page 11: I couldn't find the details of the Bayesian model in Methods.

*We had described the model in the methods but had not described it as a Bayesian model, so have amended the text accordingly on p33 line 9.*

17) Fig. 6B-C: is the y axis scaled logarithmically?

*The y axis shows the Δ reactivity, which is the difference between two logged values*

18) Page 14 and Fig. 6: as mentioned above, averaging reactivities over regions essentially loses the differential signal. While it should work better for very small regions, it could still easily miss changes in signal pattern that are characteristic of structure remodeling. I think this analysis demonstrates this point. Performing it with a more refined approach, on a transcript-by-transcript basis, and using replicates to account for biological variation could strengthen these results. Also, how were the "sequences with the biggest decrease or increase in reactivity" determined? Do they need to exceed a certain threshold or does one choose a pre-set percentile?

*We again refer to the dStruct analysis which we think nicely addresses the reviewer’s point here, as this searches for changes in reactivity pattern i.e. the average reactivity of the windows do not necessarily have to change, as certain nucleotides could increase and others decrease in reactivity, which would be suggestive of the re-folding of a hairpin. This analysis also takes biological variation into account.*

*The "sequences with the biggest decrease or increase in reactivity" were determined by selecting the window with the biggest decrease or increase in reactivity per transcript. We apologise for not stating that directly in the text and we have now added this to the manuscript on p16 lines 5-6.*

19) Fig. 6F and p. 15: I don't consider r = 0.27 to be indicative of correlation, but this may improve following stricter coverage screening.

*We accept the reviewer’s comment here and as the stricter coverage did not improve the correlation we have amended the text accordingly at the end of p17.*

20) Sequencing and bioinformatic pipeline, p. 25: the authors deal with multiple alignments to transcript variants by mapping reads to a gene's most abundant transcript. However, when several variants are abundant, they could easily adopt different structures, at least within the CDS, and this would result in reactivity misestimation due to misalignment. If the fraction of such transcripts is not too large, I suggest excluding them from analysis. Otherwise, this potential issue should be noted in the text.

*We appreciate the potential problem raised by the reviewer but do not believe that it would alter any of our findings, particularly as our analysis is focused on 5’UTRs, which vary much less between splice variants than coding sequences and 3’UTRs, as they point out. We therefore think that doing this wouldn’t justify the number of transcripts which would be lost. For example, in Figure 1C there are 1266 genes, from which there are 875 genes with just one transcript and 391 genes with more than one transcript. Within these 391 genes there are 1086 transcripts in total and when we calculate the relative abundance of the most abundant transcript within each gene, the data can be summarised by the statistics below;*

*Min. 1st Qu. Median Mean 3rd Qu. Max.*

*33.33 67.75 85.70 81.41 98.20 100.00*

*This therefore shows that for 75% of the genes, 67.75% of the reads for that gene were predicted to originate from the most abundant transcript. We note this issue in methods section on p29 lines 18-27*

21) Correlation of GC content with DMS reactivity: this is an interesting analysis but a potential caveat is a known A/C bias in stop-based DMS data, where modifications are more detectable in A than in C. This bias is protocol-dependent and can be indirectly deduced from Fig. 1SF but it is not straightforward to quantify because C is also more likely to pair than A. A possible way to account for this is to limit reactivity analysis to C only and compare C reactivity distributions between different UTR locations or at the least point out this issue.

*We would like to thank the reviewer for pointing out this important caveat. We have since repeated the analyses but plotting the reactivities of either just the As or just Cs. We still see the same pattern of reactivity across the length of the 5’UTR (Figures S5B-C), when looking at both nucleotides individually, demonstrating that this pattern is not due to a difference in the ratio of As to Cs across the length of the 5’UTR. We have added test to the manuscript to explain this result on p9, lines 6-10.*

Reviewer #2:

This study assesses the role of eIF4A in translation initiation and 5'UTR structure in the human breast cancer cell line MCF7. This is an absolutely key question in the field of translation control; where the current dogma is the helicase function of eIF4A acts to promote both recruitment of the small ribosomal subunit to the mRNA and subsequent scanning by this subunit. Both functions have been proposed to rely upon the unwinding activity of eIF4A as part of translation initiation complex. eIF4A can also serve as a regulated step in the translation process and targeting eIF4A is a potential therapeutic strategy for various human conditions.

In this paper, RNA structure has been assessed by mapping DMS reactive regions of RNA across the transcriptome. These results have then been correlated with various other datasets including a polysome enrichment dataset in the same cells. The authors conclude that eIF4A is involved in remodelling 5'UTR structures such that inhibiting its function with hippuristanol leads to local decreases and increases in DMS reactivity. Intriguingly inhibition of eIF4A leads to decreased DMS reactivity in a region just upstream of the Start codon, and mRNAs that are most translationally sensitive to eIF4A inhibition also have decreased DMS reactivity in this region. This supports a model where the helicase function of eIF4A is particularly important in remodelling the region upstream of mRNA start codon during the scanning process. I feel this paper makes a highly valuable contribution to an area of literature by taking a global approach to the study of question that has in the past been dominated by single mRNA reporter gene style studies.

*We thank the reviewer for their careful reading of our manuscript and are very pleased that they value our findings so highly.*

1) I have a couple a very minor comments on the introduction:-

a) I feel the authors should state somewhere that eIF4A1 is an ATPase and while it can function as an helicase - relative to other RNA helicases this activity is weak. So other possibilities exist for instance eIF4A could remodel protein/ RNA complexes and has been suggested to influence the 40S complex facilitating mRNA interaction (e.g. Sokabe and Fraser, 2017: Yourik et al., 2017)

b) The authors state the excess of eIF4A over other translation factors and assume that this 'excess' is 'free' eIF4A. It could be that many eIF4A molecules are involved in the translation process and therefore the stoichiometry of eIF4A involvement may not permit 'free' eIF4A?

*We thank the reviewer for these suggestions to the text and have amended the manuscript accordingly on p3 lines 10-12 and 18-19.*

2) For the polysome gradients, I have never seen the 48S peak separated from the 40S peak on standard sucrose gradients. In Figure 1B the authors label a peak as 48S. I suspect this is the 60S peak? This is often observed as a shoulder on the 80S peak. So I would interpret this gradient as 40S/48S, then 60S as a shoulder on the 80S. To support their interpretation the authors would need to conduct western/ northern blots investigating the ribosomal proteins or rRNA present in different fractions taken from the gradient.

Critically in my view, where 48S accumulation has been observed by others in the field, the most obvious feature on polysomes is halfmers, because the 40S and 48S do not separate well and most stalled 43S ribosomes are on mRNAs that already carry polysomes. So if the authors' interpretation is correct and 43S complexes are in some way accumulating on the mRNA- why do they not see halfmers?

*We thank the reviewer for their careful observations, but would like to point out that these traces were obtained with 300 mM NaCl, which disaggregates the salt-labile monosomes, whereas many labs use lower salt concentrations, which result in higher 80S peaks. We are therefore confident that the 40/60/80S peaks are correctly labelled but we agree that we don’t currently have the resolution to confidently infer the 48S peak. We have therefore re-labelled the plots with the labels “sub-polysomes” and “polysomes” and removed the sentences from the text which relied on our interpretation of an increase in a 48S peak following eIF4A inhibition (p5, lines 13-15, p10 lines 8-9 and in the first paragraph of the conclusions).*

3) P5 line 22 - the authors need to explain in more detail why the cited Figures support the interpretation that their libraries are high quality.

*We have moved this section from the methods into the results and elaborated on the justification of the coverage thresholds, as described above in response to reviewer #1 comment 1. The text can be found on p6, lines 1-7.*

a) Related to this - in the analysis shown in Figure S1H the authors show plots comparing biological replicates to each other. Ideally they should report the r-value for comparison across non-replicates i.e. are the r-values reported better between replicates than across samples generally and how much better are they?

*We would like to thank the reviewer for this suggestion and have added a correlation matrix showing r-values between all possible comparisons (Figure S2C), which nicely shows that replicates within the same sample are more highly correlated than between samples, except for the control and hippuristanol DMS (-) samples, which are also highly correlated. This makes sense, given that hippuristanol treatment shouldn’t affect the stop profile in DMS (-) samples, and we have added text to the manuscript to explain this (p6 lines 8-15).*

b) Again related to data quality - the authors state in the methods that 'the reads from each replicate were pooled'. This begs the immediate question- what is the point of doing the replicates as this information is now lost in the pooled analysis?

*This is an important point, also raised by reviewer #1. In order to assess the between replicate variation we have used the dStruct package as detailed in response to reviewer #1 comment 7.*

4) P6 lines 14-17- given what is stated, the authors should probably not refer to the region lacking the final 125n as the 3'end, maybe 3' region would be a better term?

*We agree that the recommended terminology would improve the clarity of the manuscript and have changed it within the text*

5) P6 and 7. The authors conclude that the reduction in DMS reactivity over the CDS after eIF4A inhibition is due to the absence of the ribosomes which would normally unwind secondary structure. They should explain more clearly that the decreased reactivity in the 5'UTR is unlikely due to the observed accumulation of 43S complexes, and indeed this may explain increased reactivity in certain areas of the 5'UTR

*This is an important suggestion and we have added text to this accord on p7 lines 26-27 and lines 1-2 of p8.*

6) P8 line 16-18. I wonder if there is a precedent that the authors could cite for DMS reactivity being almost immune to protein or ribosome binding?

*We were unable to find a reference for this, which is why we wanted to assess this ourselves*

7) P9 line 11. It seems very odd to use a HEK293 dataset here - especially when later in the paper the authors state that transcripts vary significantly between HEKs and MCF7 cells. Perhaps the authors could add a sentence here explaining why the approach is a valid one.

*Although data from MCF7 cells would obviously be more reliable, there are currently none available. We do however show that the hippuristanol sensitive mRNAs in HEK293 cells from Iwasaki et al. are also not enriched with upstream initiation potential (Figure S7F). We do not state that transcripts vary significantly between HEKs and MCF7 cells, rather that the standard annotation of the human transcriptome has very poor 5’ end annotation. As well as being the only approach available, we think this is valid, as we only choose genes which have no detected alternative translation start sites, suggesting no potential for upstream initiation. This is different from identifying genes that do undergo upstream initiation events in one cell type, and expecting the same to be true in an alternative cell type. We have added a sentence explaining this to the manuscript on p10 lines 13-16.*

8) P9 line 16 'strongly argues' should be toned down just 'argues' is sufficient.

*We have modified the text accordingly.*

9) P10 lines 1-8, is quite confusingly written - initially it was not clear whether the authors had performed a ribosome density mapping technique (ribosome profiling), or taken a polysome enrichment approach. They should make this clearer and justify their approach in the text. How do these data compare with the data from Iwasaki et al 2016 using HEK293 cells and Rocaglates. There should be some correlation?

*We apologise that it was not clearer in the text that we have carried out polysome profiling, rather than ribosome footprinting. We have modified the text to make this clearer on p11 lines 7-8. We have added justification of the use of polysome profiling over ribosome footprinting on p11 lines 10-14.*

*To show the overlaps between previously published eIF4A-dependent mRNAs, we have also added a Venn diagram to the supplemental information (Figure S7A), to show the overlap between our data and the Hipp-sensitive mRNAs from Iwasaki et al. (2016) (2) and the eIF4A1-dependent mRNAs identified following knock-down of eIF4A1 with siRNA in MCF7 cells, from Modelska et al. (2015) (3). This shows a higher degree of overlap between the eIF4A1-dependent mRNAs from Modelska et al. (we identified 33.7% of those from Modelska et al.) compared with the Hipp-sensitive mRNAs from Iwasaki et al. (we identified 17.3% of those from Iwasaki et al.). There are likely two reasons for the better overlap with the eIF4A1-dependent mRNAs than the Hipp-sensitive mRNAs, the first being that the same cell line was used and the second that the same technique (polysome profiling rather than ribosome footprinting) was used. Note also that Iwasaki et al. used 1 µM hipp, compared to our use of 150 nM. We have added text to the manuscript explain this data on p13 lines 8-17.*

10) P10- In the last paragraph the logic is difficult to follow. The authors present two possible explanations but then how the experiments relate to these possibilities is difficult to understand. Plus they use the phrase "To rule out the possible explanation"- this should be something like to test, assess or distinguish.

*We agree that that the uTIS analysis does not really address which of the two possibilities are correct and have removed that portion of text, which he hope clarifies the passage considerably. We also changed "To rule out the possible explanation" to “to test whether”.*

11) P11- Line 4 "excludes the possibility" is too strong here - there are too many caveats - for instance if I understand correctly the data being compared are from different cell lines- so the conclusion can never be unequivocal.

*We have changed the text to “suggesting that reduced structure just upstream of CDSs in highly translated mRNAs is not due to upstream translation initiation”.*

12) P11- Line 19 typo- should be that instead of than?

*This is now corrected in the text and we thank the reviewer for noticing the error*

13) In the discussion I would like to see the authors place their observations into a more holistic view of the role of RNA helicases in translation initiation - key things to add would be:-

a) the role of other helicases - for example DHX29, Ded1/DDX3, Vasa/DDX4, RNA helicase A (RHA/DHX9), and Dhh1/RCK/DDX6

b) Potential roles of eIF4A in ribosome recruitment - possibly by remodelling the 40S subunit?

*We have added text to the discussion (2nd paragraph) and thank the reviewer for noticing these omissions. We have included text on the roles of Ded1p, DDX3 and DHX29, but feel discussion of the other helicases in the list above might be better suited to a review article.*

*References:*

*1. Choudhary K, Lai YH, Tran EJ, Aviran S. dStruct: identifying differentially reactive regions from RNA structurome profiling data. Genome biology. 2019;20(1):40.*

*2. Iwasaki S, Floor SN, Ingolia NT. Rocaglates convert DEAD-box protein eIF4A into a sequence-selective translational repressor. Nature. 2016;534(7608):558-61.*

*3. Modelska A, Turro E, Russell R, Beaton J, Sbarrato T, Spriggs K, et al. The malignant phenotype in breast cancer is driven by eIF4A1-mediated changes in the translational landscape. Cell death & disease. 2015;6:e1603.*

**Second round of review**

**Reviewer 1**

The authors nicely addressed most of my concerns. The comments below pertain mostly to the newly added analysis.

* The results of the two analyses (new and old) need to be reconciled. Fig. S4 and its discussion on page 7 show that the control is more reactive (less structured) than hipp in the CDS and 5’ UTR. Furthermore, for the CDS, this seems to hold for most transcripts (see scatter plot in panel B). Similarly, the meta analysis in Fig. 1 supports this trend. On the other hand, Fig. 6 shows that dStruct analysis implies the opposite trend, i.e., hipp is more reactive than control for all regions but the 5’ UTR/CDS. However, the discussion on page 19 is limited to the 3’ UTR or at least I did not find any reference to these results for the 5’ UTR and CDS. I think the authors need to address these discrepancies in the main text, possibly by explicitly referring to the scatter plots in Fig. 6 and/or reminding the reader that the two analyses were not performed on the same dataset due to different filtering strategies and because of dStruct’s prior selection of differentially reactive windows. It is also possible that upon applying same coverage filtering, the trends would become more similar.

Note that this issue also relates to my prior comment no. 5 that these small differences are unlikely to be meaningful because local differential effects appear to be masked by the global averaging. If reconciling the differences turns out to be too difficult or too demanding, then perhaps reconsider moving this discussion to the Supp.

* Page 19 and Fig. 6: I am not sure why the coverage constraint was relaxed in the dStruct analysis. The motivation may have been to include more transcripts, but this creates some inconsistency with the authors’ earlier analysis, as I commented above. Additionally, stricter filtering could potentially improve the results shown in Figure 6, and in particular, the main result of this work (panel B). Although fewer differential regions will be detected, the p-values in panel B may change in favor of the 5’ UTR and/or the top differentially reactive regions may be more enriched in 5’ UTR sequences from 4A-dependent transcripts than previously. Did the authors try different filtering strategies in their analysis?

* Page 19 and Fig. S8: following the dStruct analysis, an FDR cutoff of 0.25 was used. This is quite liberal as on average, one of 4 discoveries, i.e., regions deemed differential, is false. FDRs of 0.05 and 0.1 are more commonly applied, but I can see from Fig. S8 that not many differential regions will remain. Did the authors consider using more conservative FDRs, such as 0.125 or 0.15, in their analysis? Similarly to my previous comment, this could potentially strengthen some of the results, e.g., when assessing the enrichment in 4A-dependent transcripts.

* Typo on page 15: “There a small, yet…”

* Typo on Fig. 2E axis label (contorl)

* Figure 5B: the listed p values indicate that the most statistically significant change is in fact for windows of size 15 nts and not 20nt as stated in the manuscript (page 16). Also note that this optimal length is also very close to the modes/peaks of most dStruct window length distributions (Fig. S8B).

* Page 19: when reporting results of multiple hypothesis tests (after p-values have been modified to control the FDR), the output values are commonly called FDR adjusted p-values, or q values. To avoid confusion, I suggest replacing FDRs with FDR adjusted p-values.

* Page 19: “assigned each window into one of five groups.”

* Typo on page 20: “… did not return and enriched motifs.”

* Figure S4: legend with line colors is missing.

* Page 8, last paragraph: my interpretation of the hipp-induced increase in Gini index is that structures are more stable or that transcripts are more “structured” because reactivities are overall closer to the extremes of their range. This makes sense if a helicase is acting on these regions. I believe that localized changes in structure could easily manifest as arbitrary changes in Gini indices as opposed to their increase. For example, why should the refolding of a hairpin increase the Gini index? I therefore recommend rewording the end of the paragraph.

**Reviewer 2**

The authors have addressed my comments

**Authors Response**

**Point-by-point responses to the reviewers’ comments:**

Reviewer #1: The authors nicely addressed most of my concerns. My comments below pertain mostly to the newly added analysis.

* The results of the two analyses (new and old) need to be reconciled. Fig. S4 and its discussion on page 7 show that the control is more reactive (less structured) than hipp in the CDS and 5' UTR. Furthermore, for the CDS, this seems to hold for most transcripts (see scatter plot in panel B). Similarly, the meta analysis in Fig. 1 supports this trend. On the other hand, Fig. 6 shows that dStruct analysis implies the opposite trend, i.e., hipp is more reactive than control for all regions but the 5' UTR/CDS. However, the discussion on page 19 is limited to the 3' UTR or at least I did not find any reference to these results for the 5' UTR and CDS. I think the authors need to address these discrepancies in the main text, possibly by explicitly referring to the scatter plots in Fig. 6 and/or reminding the reader that the two analyses were not performed on the same dataset due to different filtering strategies and because of dStruct's prior selection of differentially reactive windows. It is also possible that upon applying same coverage filtering, the trends would become more similar.

Note that this issue also relates to my prior comment no. 5 that these small differences are unlikely to be meaningful because local differential effects appear to be masked by the global averaging. If reconciling the differences turns out to be too difficult or too demanding, then perhaps reconsider moving this discussion to the Supp.

*We agree that the inconsistency between these Figures could cause some confusion to the reader and we have added text to elaborate on this issue (this is included in the tracked changed version of the manuscript emailed separately). We do not feel however that this is an issue that should cause any concern as Fig S4 is plotting the average reactivities across the entire lengths of the UTRs/CDS, while Figure 6A is plotting the average reactivity of just the differentially reactive windows identified by dStruct. Due to these differences we weren’t too surprised to see that these two analyses do not show the same trend, and we were greatly reassured by the data showing that in 4A-dep 5’UTRs both the mean and median delta reactivity of the windows is negative, and importantly more negative than 4A-indep 5’UTRs (Figure 6B), which therefore shows that the differential windows identified in 4A-dep mRNAs are gaining in overall structure.*

* Page 19 and Fig. 6: I am not sure why the coverage constraint was relaxed in the dStruct analysis. The motivation may have been to include more transcripts, but this creates some inconsistency with the authors' earlier analysis, as I commented above. Additionally, stricter filtering could potentially improve the results shown in Figure 6, and in particular, the main result of this work (panel B). Although fewer differential regions will be detected, the p-values in panel B may change in favor of the 5' UTR and/or the top differentially reactive regions may be more enriched in 5' UTR sequences from 4A-dependent transcripts than previously. Did the authors try different filtering strategies in their analysis?

*We appreciate the reviewers concerns that using a lower coverage constraint creates some inconsistency with the earlier analysis. However, we feel this actually strengthens the data for the following reasons. The coverage constraints used for Figures 1-5 ensures that there is sufficient correlation in reactivities between replicates, to reduce noise, thereby ensuring changes in reactivity are more likely to occur as a result of hippuristanol treatment, rather than a change within just one of the replicates in either condition. The dStruct package tests this directly, by measuring differential reactivity patterns within the replicates and between samples, ensuring that the differential windows identified have higher variation between control and treated samples than between replicates. By lowering the coverage threshold for the input, this allows the analysis of less abundant transcripts, which will strengthen the data and increase the numbers of differentially reactive windows for downstream analysis. We therefore feel that the current analysis is the most appropriate and at the viable limit of our data, given that there are currently only 87 differentially reactive windows in 4A-dependent 5’UTRs.*

* Page 19 and Fig. S8: following the dStruct analysis, an FDR cutoff of 0.25 was used. This is quite liberal as on average, one of 4 discoveries, i.e., regions deemed differential, is false. FDRs of 0.05 and 0.1 are more commonly applied, but I can see from Fig. S8 that not many differential regions will remain. Did the authors consider using more conservative FDRs, such as 0.125 or 0.15, in their analysis? Similarly to my previous comment, this could potentially strengthen some of the results, e.g., when assessing the enrichment in 4A-dependent transcripts.

*We agree that the FDR threshold of 0.25 is quite liberal and appreciate the reviewer’s suggestion that lowering this could improve some of the results. We also plotted panel B from Figure 6 using an FDR of 0.15 (this not possible with 0.125) and this did not alter the differential between eIF4A-dependent and independent 5’UTRs, which remained statistically significant. However, as there are only 23 eIF4A-dependent windows in the 5’UTR with an FDR less than 0.15, we did not feel this to be enough to bin the data accurately. We therefore feel that keeping the figure in its current form is the best option.*

* Typo on page 15: "There a small, yet…"

W*e thank the reviewer for noticing this error and this has been rectified in the text*

* Typo on Fig. 2E axis label (contorl)

*We thank the reviewer for noticing this error and have amended the figure accordingly*

* Figure 5B: the listed p values indicate that the most statistically significant change is in fact for windows of size 15 nts and not 20nt as stated in the manuscript (page 16). Also note that this optimal length is also very close to the modes/peaks of most dStruct window length distributions (Fig. S8B).

*We would like to thank the reviewer for pointing out this discrepancy, which we have corrected in the text. We have also added a sentence to mention the agreement with the dStruct analysis to the manuscript and thank the reviewer for this suggestion (both changes are shown in the attached tracked change manuscript).*

* Page 19: when reporting results of multiple hypothesis tests (after p-values have been modified to control the FDR), the output values are commonly called FDR adjusted p-values, or q values. To avoid confusion, I suggest replacing FDRs with FDR adjusted p-values.

*We agree with the reviewer’s suggestion and have altered the figure accordingly*

* Page 19: "assigned each window into one of five groups."

*We thank the reviewer for noticing this error and this has been rectified in the text*

* Typo on page 20: "… did not return and enriched motifs."

*We again thank the reviewer for noticing this error and have rectified the text*

* Figure S4: legend with line colors is missing.

*While we couldn’t find any missing line colour legends in Figure S4, there were missing ones in Figure S5, which we have presumed is what the reviewer had noticed, and we have added these to the amended Figure.*

* Page 8, last paragraph: my interpretation of the hipp-induced increase in Gini index is that structures are more stable or that transcripts are more "structured" because reactivities are overall closer to the extremes of their range. This makes sense if a helicase is acting on these regions. I believe that localized changes in structure could easily manifest as arbitrary changes in Gini indices as opposed to their increase. For example, why should the refolding of a hairpin increase the Gini index? I therefore recommend rewording the end of the paragraph.

*We appreciate this insight and agree with the interpretation. We have altered the text accordingly.*
